# Expedient single-round selection of hyper-modified aptamer targeting insulin receptor from over-represented dually nucleobase-modified DNA libraries

**DOI:** 10.1038/s41467-026-73676-y

**Published:** 2026-05-27

**Authors:** Pablo Alberto Franco-Urquijo, Marek Ondruš, Jaroslav Kurfürst, Jana Škerlová, Irena Selicharová, Lucie Mužíková Čechová, Hana Šváchová, Alena Semerádtová, Anatolij Filimoněnko, Adéla Fejfarová, Jiří Homola, Tomáš Kouba, Michal Hocek

**Affiliations:** 1 Institute of Organic Chemistry and Biochemistry, Czech Academy of Sciences, Flemingovo nam. 542/2, Prague 6, Czech Republic; 2 Institute of Photonics and Electronics, Czech Academy of Sciences, Chaberská 1014/57, Prague 8, Czech Republic; 3 Department of Organic Chemistry, Faculty of Science, Charles University in Prague, Hlavova 8, Prague 2, Czech Republic

**Keywords:** Nucleic acids, DNA, Nucleic-acid therapeutics

## Abstract

Discovery of functional nucleic acids from randomized libraries typically relies on multiple, time-consuming iterative rounds of in vitro selection with low success rate. Here, we present a single-round selection strategy for rapid screening of multiple over-represented nucleobase-modified DNA libraries and various selection conditions, capable of identifying high-affinity modified aptamers. Double partition followed by amplification of eluted sequences, NGS analysis and clustering provides fast identification of aptamer candidates. Screening of modified DNA libraries containing modified adenine and uracil nucleotides bearing hydrophobic aromatic phenyl and indole moieties results in development of an aptamer binding human insulin receptor with sub-nanomolar affinity and exquisite specificity. Cryo-EM structure reveals the importance of each aromatic modification, either in stabilizing the secondary structure or facilitating interactions with the protein surface. This approach addresses the main drawbacks of aptamer selection and has potential for high-throughput screening and accelerating the development of next-generation aptamers for diagnostics or therapeutics.

## Abstract (web summary)

The discovery of aptamers generally relies on laborious multiple-round selections with low success rate. Here, authors report the rapid, single-round selection of highly specific base-modified aptamers from overrepresented modified DNA libraries containing two different modified nucleotides.

## Introduction

Nucleic acid sequences that bind with high affinity and specificity to their targets are named aptamers and are typically selected by Systematic Evolution of Ligands by Exponential enrichment (SELEX)^1,2^. This general methodology led to the development of specific aptamers against a wide range of targets – from small molecules^3^ to proteins^4,5^ or whole cells^6,7^, as well as other functional nucleic acids, i.e. DNAzymes^8–11^. This powerful strategy has produced novel catalytic and binding motifs by combining two of its main components: i) an extensive sequence space from diverse nucleic acid pools and ii) multiple rounds of selection. However, the iterative selection process is very tedious and time-consuming. Moreover, despite the very high sequence and secondary structure diversity of the natural DNA or RNA libraries, the diversity of functional groups capable of specific interactions with target, in particular hydrophobic interactions with proteins, is rather limited. To increase chemical diversity in aptamer selections, an introduction of base-modified nucleotides began in the 1990s with the application of 5-(1-pentynyl)−2’-deoxyuridines^12^ and continued to increase in the last decade with quite a large portfolio of functional groups typically linked to position 5 of 2’-deoxyuridines^13^. This approach led to development of slow-off-rate modified aptamers (SOMAmers) which demonstrated that the introduction of one^14,15^ or even two^16,17^ different modifications resulted in the selection of aptamers with improved affinity and epitope coverage. Aptamers with hydrophobic aromatic^5^, cyclooctatetraene^18^, or cyclic aliphatic^19^ modifications suggest that an enhanced chemical diversity and interaction with hydrophobic protein patches can allow access to a larger fraction of epitopes compared with non-modified aptamers^14^. Moreover, a hydrophobic naphthyl moiety connected through a flexible methyl-carboxamide linker allowed the selection of a highly compact aptamer^20^, suggesting that even a short modified sequence space might be enough to obtain high-quality aptamers.

While the classical multiple-round SELEX is analogous to Darwinian evolution^21^, screening of libraries by single-round selections (SRSs) is also feasible, although technically challenging and with a low success rate. The main problems include a low frequency of binders in the initial pools^22^ and their insufficient separation from non-binders. Hence, there were only a few scattered examples of successful SRSs of aptamers that overcame the separation problem by applying high-partition-efficiency methods, such as capillary electrophoresis^23–25^ or microfluidics^26,27^. Despite this, these systems face technical limitations, such as a low amount of library to be used, specialized equipment, limited buffer conditions, or reliance on pre-enriched libraries, therefore, the laborious multiple-round SELEX remained the preferred approach. More recently, high partition efficiency was alternatively achieved using a non-fouling PEG hydrogel that minimizes nonspecific adsorption^28^. In conventional SELEX, libraries with a random region of about 30-60 nucleotides are typically used to allow folding of complex secondary structures that may be needed for the formation of functional domains^29^. This size generates an extensive sequence space (~10^24^ unique sequences), but only ~10^15^ can be experimentally tested in SELEX. Although these types of libraries are commonly used, the use of shorter randomized regions offers the over-representation of sequences when less than 24 randomized positions are used. Because these libraries contain thousands of copies of each sequence, it enables monitoring of individual sequence enrichment without the need for in vitro evolution. However, the successful application of over-represented libraries composed of canonical nucleotides has been limited to a few works, highlighting their major drawbacks–a reduced sequence space^30^ (e.g. ~10^9^ for N15) and structural diversity^8,31^. We hypothesized that the use of short randomized libraries functionalized with a combination of two modified nucleotides can compensate for the structural benefits of longer canonical libraries, increase the number of binders by expanding the diversity of functional groups and thus overcome the inherent limitations of SRSs and improve the success rate of the SRS of aptamers against protein targets.

In this work, we design and synthesize modified over-represented libraries displaying two different (hetero)aromatic hydrophobic moieties, one connected through a rigid and the other via a flexible linker, and test their effectiveness for SRSs. As a proof-of-concept, we implement an SRS approach targeting the Human Insulin Receptor (HIR) apo form and identify a highly modified aptamer. HIR belongs to the tyrosine kinase receptor family composed of two extracellular α-subunits and two transmembrane β-subunits that mediate various metabolic and mitogenic functions, which make HIR a relevant target for therapeutic applications^32–37^. Several examples of highly-potent HIR aptamers have been previously reported containing naphthyl-linked SOMAmer-type nucleotide selected via classical 8-round SELEX, exerting agonist activity on the receptor^38–40^ and two cryo-EM structures of the aptamers with the target are available^41^. This provides a solid background for our study, where we want to demonstrate the power of the combination of SRS and over-represented libraries with the use of two base-modified nucleotides in expedient selection of functional nucleic acid ligands that involves two consecutive partition steps. We also aim at the full characterization of the resulting dually modified aptamer, validation of its affinity, specificity, and insulin antagonistic activity, verification of the role of the aromatic modifications and linkers in the binding to the target, as well as determination of the structure of the aptamer-protein complex by cryo-EM and comparison with the previously reported singly-modified HIR aptamers^38–40^.

## Results

### Modified libraries significantly changed distributions of sequence abundances

**Fig. 1:**
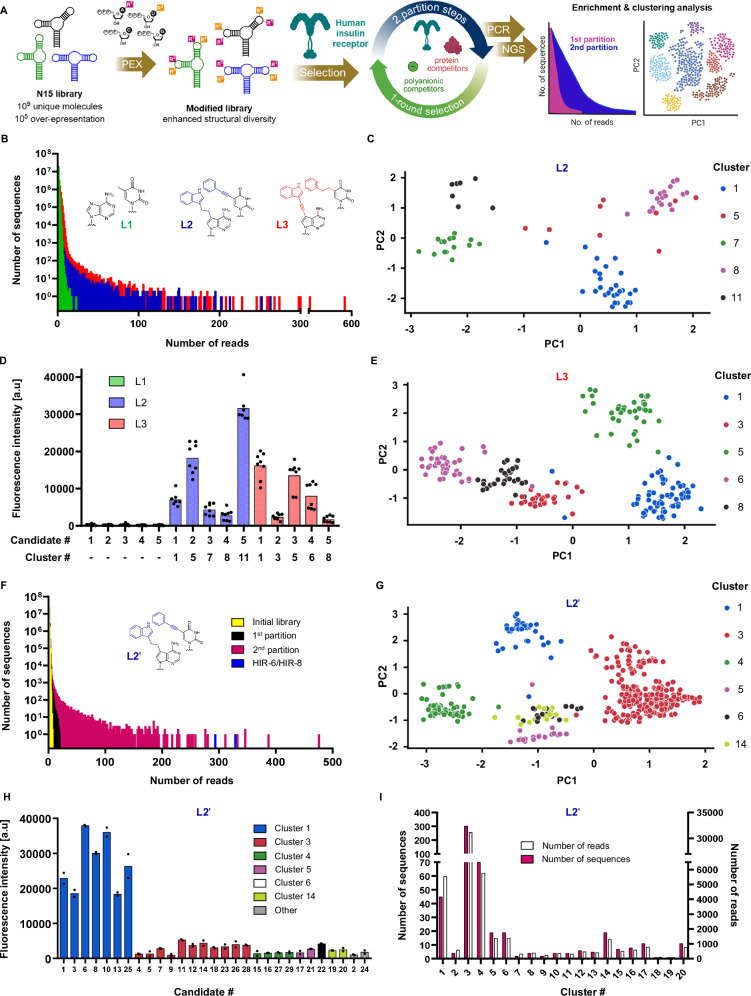
Single-round performance of over-represented libraries. **A** Single-round aptamer selection scheme. Created in BioRender. Ondrus, M. (https://BioRender.com/myesdz8). **B** Distributions of sequence abundances within 2nd partition step of natural L1 and modified L2 and L3 over-represented N15 libraries. **C** K-mer PCA plot of L2-identified clusters. **D** FNBA assay of top clustered sequences from L1, L2 and L3; *n* = 2, each performed as technical quadruplicate. **E** K-mer PCA plot of L3-identified clusters. **F** Distributions of sequence abundances within both partition steps of modified L2’ library (independent SRS from L2). **G** K-mer PCA plot of L2’-identified clusters. **H** FNBA assay of top candidates identified from various L2’ selection clusters; *n *= 1, performed as technical duplicate. **I** Quantification of NGS reads and sequences of L2’-identified clusters. Source data are provided as a Source Data file.

SRSs generally can be performed in short time and easily parallelized and scaled-up. However, the inherent problems are a limited number of binders in the initial pools and their insufficient enrichment after one selection round. To overcome this problem and significantly increase the chemical and structural diversity of the pool, we engineered dually modified over-represented libraries. The libraries were constructed by Primer EXtension reaction (PEX) using two hydrophobic aromatic nucleotides (indole-modified dATP and phenyl-modified dUTP) and two non-modified nucleotides (dGTP and dCTP) (Fig. 1A). The indole and phenyl groups were attached either via flexible ethyl or rigid ethynyl linkers. Although alkyl linkers might allow a higher degree of rotational freedom compared to ethynyl or SOMAmer carboxamide linkers, we previously showed that using two or more flexibly attached modified nucleotides makes difficulties in enzymatic synthesis of hypermodified DNA^42^. Therefore, we aimed for a balanced combination using one flexible and one rigid linker. The G-C base pairs, typically involved in stem formation, were left unmodified to promote higher structural stability of secondary structures.

To investigate the landscape of enriched binding sequences and the effect of hydrophobic modifications compared with the unmodified ssDNA library (L1), we prepared two modified libraries with a 15nt randomized region, generating ~10^9^ unique sequences, and two constant 20nt primer regions to allow PCR amplification. The first modified library (L2) contained 5-(Phenyl-ethynyl)−2’-deoxyuridine (dU^EPh^) and 7-(Indole-ethyl)−2’-deoxyadenosine (dA^AIn^), while the second modified library (L3) contained 5-(Phenyl-ethyl)−2’-deoxyuridine (dU^APh^) and 7-(Indole-ethynyl)−2’-deoxyadenosine (dA^EIn^) (Fig. 1B). Modified libraries were produced by PEX using a 55nt library template and a 5’-Cy5-labeled reverse primer that allowed their precise HPLC purification and analysis on denaturing polyacrylamide gel (dPAGE) (Supplementary Fig. 2). Both nucleotide combinations presented a slower electrophoretic mobility compared with the unmodified DNA library (L1) due to the introduction of modifications and a higher mass-to-charge ratio.

Having the three libraries in hand, we proceeded with the selection for aptamers binding to HIR. The selections were performed under consistent conditions across all three libraries. They involved two consecutive partitioning steps with decreasing concentration of His-tagged HIR-functionalized magnetic beads (Supplementary Table 3). Each SRS was conducted with 300 pmol of library (~168,500 copies of each sequence) and incubated for 30 min with 1 µM HIR, followed by a second partition step with a sixfold lower concentration. Additionally, protein and polyanionic competitors were introduced to isolate specifically-binding sequences. Aptamer complexes were separated using a magnet and bound sequences were thermally eluted and amplified by qPCR to determine the optimal number of cycles. The eluted sequences were amplified using PCR with adapters and indexes and purified through agarose gel extraction (Supplementary Fig. 6).

Pooled samples were sequenced using next-generation sequencing (NGS) and bioinformatic analysis revealed a significant difference in the distribution of sequence abundances between the standard DNA library (L1) and the two modified libraries (L2 and L3). The modified libraries showed a substantial increase in the number of enriched sequences compared to both the unmodified library (Fig. 1B) and their initial libraries (Supplementary Figs. 10–12). The initial sequence over-representation allowed us to identify enriched target-binding sequences without requiring multiple iterative selection rounds, despite employing a shorter sequence space compared to conventional SELEX. Additionally, cluster analysis of the selected modified pools allowed for the identification of sequence families, simplifying the analysis since clusters often behave as quasi-species in response to the local fitness landscape^43^. Analyses of the top enriched sequences led to the identification of several clusters of sequences for the selected modified pools (Fig. 1C, E, and Supplementary Tables 6, 7). Interestingly, the different linker flexibility between the L2 and L3 libraries resulted in unrelated enriched sequences generated from the same sequence space, similar to previous observations where different modified dNTPs produced different aptamers^14,16^. Due to the insufficient enrichment of sequences within the selected unmodified L1 pool, clustering did not yield well-defined clusters. Seed sequences (Supplementary Table 2) from five clusters of each L2 and L3 library were synthesized individually (Supplementary Figs. 20–21) and screened together with top five (by count) sequences from L1 for affinity to HIR using fluorescent Ni-plate binding assay (FNBA) - in which the HIR was immobilized onto the plate surface and where the capability of the Cy5-labeled aptamers to remain bound to HIR after several washing steps is indicated by their fluorescence intensities (Fig. 1D). The L2 library contained higher-affinity sequences compared to those from L3, while sequences from the unmodified library (L1) showed negligible binding.

### Second partition step allowed the identification of aptamer sequences independently of primers

Due to relatively short N15 randomized region, there was a high probability that the primer regions will be involved in formation of aptamer structure. To validate the effect of hydrophobic modifications independently of primer design, another SRS with a new library (L2’) using a new set of primers was performed. In addition, we investigated the impact of each partition step on the number of enriched sequences. Changes in the distribution of enriched sequences drastically increased after the second partition step, while the first partition did not produce a significant change (Fig. 1F). Although the use of several partition steps has been previously described in non-SELEX protocols by using high-partition-efficiency methods^44^, their application in SRSs with modified over-represented libraries has not been previously reported. In our protocol, this combination of SRS and two partition steps enabled a significant enrichment of binding sequences without consecutive cycles of amplification. Further analysis revealed the presence of three main clusters (1, 3 and 4) containing the sequences with the highest number of reads and three other less defined clusters (5, 6 and 14) (Fig. 1G,I).

To confirm the binding affinity relatedness obtained from clustering results, we enzymatically synthesized twenty-nine individual representative sequences from the main L2’ clusters (Supplementary Tables 2 and 8). Modified aptamer candidates were produced using a PEX with 5’-biotinylated reverse complementary template, 5’-Cy5-labeled reverse primer, and strand separated (Supplementary Figs. 22–26) as mentioned before, and screened by FNBA assay to evaluate their binding affinity to HIR. Screening of the candidates revealed different affinities between the clusters, explained by the different conserved motifs. Sequences from cluster 1 with a UUUAACGUAANA consensus motif exhibited the highest binding affinity compared to other clusters (Fig. 1H). Interestingly, cluster 1 from L2’ selection shared UUUAACGUAA motif with cluster 11—the highest affinity cluster from the previous L2 selection (Supplementary Fig. 19). Two separate selections successfully yielded enriched sequences regardless of primer design, although primers may cause some variations in the distribution of sequence abundances and cluster formation. From this screening, two candidates (HIR-6 and HIR-8) from cluster 1 of L2’ selection were chosen for further characterization and validation as HIR aptamers.

### Number of randomized positions matters – higher sequence over-representation determines single-round performance of modified libraries

To investigate the influence of the random (N) region length and the impact of sequence over-representation for aptamer selections, we performed additional SRSs with N13, N17, N19 and N22 variations of the L2’ library (Supplementary Fig. 3). These libraries cover an over-representation range from millions of copies of each sequence (in the case of N13) to only 10 copies (in the case of N22). After NGS sequencing, all data were analyzed for their distribution of sequence abundances as described previously. While N13 showed a distribution similar to N15 for most enriched sequences, N17 already showed limited enrichment, and N19 and N22 selection libraries exhibited a similar number of reads as their initial libraries (Supplementary Figs. 13–17). In other words, as the sequence space increases to N19 and beyond and the over-representation significantly drops, the probability of identifying binding motifs by clustering the top sequences drastically decreases and relies solely on large-scale affinity testing. On the other hand, the identification of the HIR-6-like motif (GUUUAACGUAACA) in the top-count sequences of both N13 and N17 libraries demonstrates the feasibility of rapid identification of another HIR-6-like aptamer (Supplementary Tables 9–10). To validate N13 and N17 selections, the seed sequences of top five clusters were synthesized and screened for their binding affinity by FNBA (Supplementary Figs. 27–28 and 34). Interestingly, the seed sequences containing the previously identified HIR-6-like motif exhibited the highest binding affinity compared with other sequences obtained from the same sequence space, providing evidence that N17 and shorter modified randomized regions can be applied for our rapid SRS approach and repeatedly generate high-affinity aptamers.

### Modified aptamers showed high affinity and specificity for HIR target

**Fig. 2:**
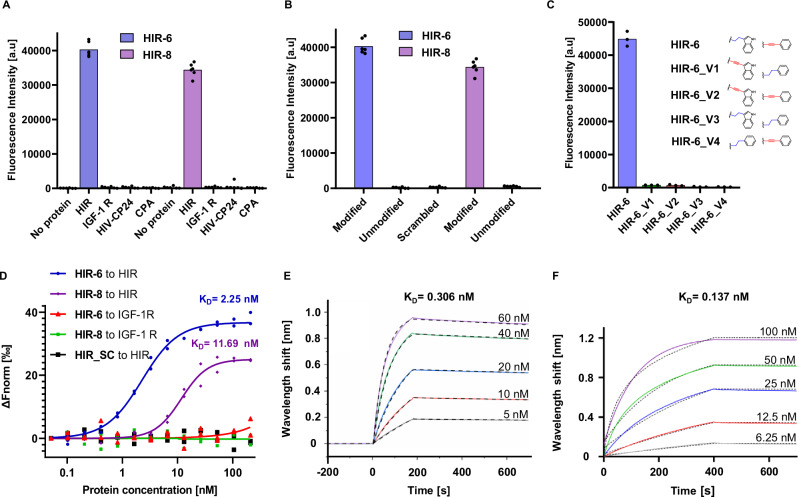
Validation of aptamer candidates. **A** FNBA assay showing target specificity of HIR-6 and HIR-8 to HIR; *n* = 2, each performed as technical triplicate. **B** HIR-6 and HIR-8 sequence specificity to HIR compared to scrambled sequence (HIR_SC, shared for both aptamers) and unmodified sequences (HIR-6_N and HIR-8_N) using FNBA assay; *n* = 2, each performed as technical triplicate.** C** HIR-6 linker and indole moiety dependency tested via FNBA assay; *n* = 1 performed as technical triplicate. **D** MST analysis of HIR-6, HIR-8 and HIR_SC to HIR and structure-related IGF-1 R, obtained by titrating increasing protein concentrations to 5’-Cy5-labeled aptamer sequences; *n* = 2 independent experiments. **E** Representative SPR sensorgram of HIR-HIR-6 interaction obtained by titrating different HIR concentrations to immobilized 5’-biotinylated HIR-6. Indicated K_D_ value is the average of *n* = 3 independent experiments. **F** BLI sensorgram showing HIR-HIR-6 interaction at different HIR concentrations, *n* = 1. Source data are provided as a Source Data file.

The selected sequences HIR-6 and HIR-8 were further evaluated using a FNBA assay to assess their specific binding to HIR. Several selected related and unrelated proteins in combination with HIR-6 and HIR-8 were tested and have not shown any increase in fluorescence intensity (Fig. 2A). Both HIR-6 and HIR-8 aptamers have shown remarkable specificity as they did not bind to Insulin-like Growth Factor 1 Receptor (IGF-1 R), which shares more than 50% sequence identity with HIR^45^. We have also tested modified scrambled sequence (HIR_SC) containing the same proportion of modified nucleotides, as well as unmodified sequences (HIR-6_N and HIR-8_N) and also none of them showed any binding to HIR (Fig. 2B). Aptamers HIR-6 and HIR-8 were also examined by microscale thermophoresis (MST), revealing binding affinities of 2.25 and 11.69 nM, respectively (Fig. 2D). Additionally, the higher-affinity HIR-6 aptamer was analyzed using surface plasmon resonance (SPR) and biolayer interferometry (BLI), both confirming sub-nanomolar affinities of 306 pM and 137 pM, respectively (Figs. 2E, F). The binding affinities of HIR-6 and HIR-8 are of similar or better level compared to the previously reported singly-modified HIR aptamers^38,39^ selected by conventional multi-round SELEX and to monoclonal antibodies^46^. This clearly demonstrates that the single-round selection combined with two modified dNTPs enables an expedient selection of (sub)nanomolar affinity aptamers avoiding the time-consuming multi-round SELEX or high-throughput testing of hundreds of individual candidates.

### HIR-6 binding affinity is dependent on aromatic moieties and the linkers

Although it has been repeatedly shown that the binding properties of modified aptamers obtained through in vitro evolution rely on their modifications for binding^14^, the impact of the linkers tethering these modifications to the nucleobase has never been specifically explored. In our study, the results from the SRSs showed that different linkers connecting the aromatic moieties produced different aptamer candidates from the same library sequence space. Therefore, we decided to investigate the importance of modifications and the rigidity or flexibility of their linkers for binding affinity of HIR-6 sequence. To achieve this, three additional variants of HIR-6 sequence (V1-V3, Fig. 2C), each with a different combination of linkers, were enzymatically synthesized (Supplementary Fig. 31, Supplementary Table 12). Additionally, phenyl-modified dATP (dA^APh^TP), with a phenyl ring tethered via a flexible ethyl linker, was synthesized (Supplementary Fig. 1, Supplementary Figs. 45–51) and used for the enzymatic production of variant HIR-6_V4 in which the indole moiety was replaced by a phenyl group. All four HIR-6 variants (HIR-6_V1-V4) were tested using the FNBA assay (Fig. 2C), and the results were confirmed by MST (Supplementary Fig. 36). The plate assay revealed that the only strong fluorescence signal was from parent HIR-6, with no signal from the variants. Notably, swapping the linkers (HIR-6_V1) or using either two rigid or two flexible linkers (HIR-6_V2, HIR-6_V3) led to a complete loss of binding ability, highlighting the essential importance of not only the nature of the aromatic modifications but also of the linkers for the binding affinity to HIR.

### HIR-6 aptamer inhibits insulin signaling by competing for the insulin receptor

**Fig. 3:**
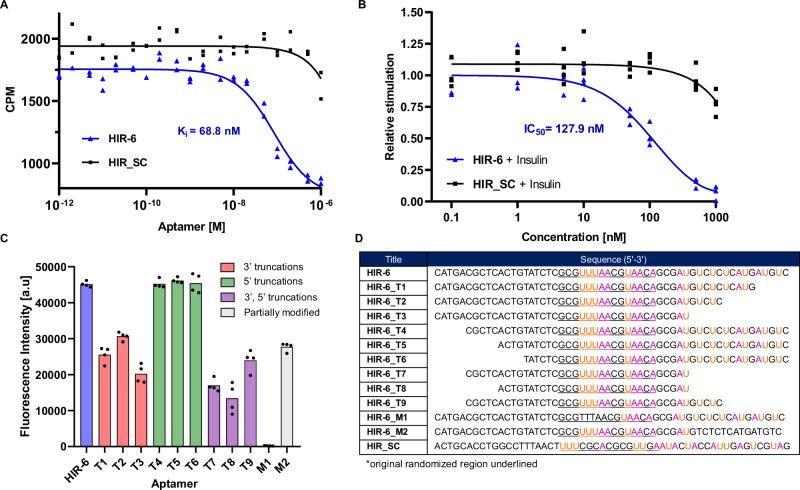
HIR-6 antagonism activity and truncation study. **A** HIR-6 binding competition with [^125^I]monoiodotyrosyl-A14-insulin for HIR-A isoform in human IM-9 lymphocyte cell membranes using increasing concentrations of HIR-6 and HIR_SC. Decrease in radioactively labeled insulin bound to HIR was measured (CPM) and representative plot is shown. K_i_ was calculated from *n* = 2, each performed as technical duplicate (Supplementary Fig. 38). **B** Antagonistic activity of HIR-6 to insulin-stimulated receptor phosphorylation. Mouse fibroblast cells expressing HIR-A were stimulated with 10 nM insulin and exposed to increasing concentrations of HIR-6 and HIR_SC. The level of phosphorylation relative to insulin alone (Supplementary Fig. 41) was used to determine IC_50_ value of HIR-6 from combined data of *n* = 2, each performed as technical duplicate. **C** FNBA assay for HIR-6 truncated and mutated sequences; *n* = 1, performed as technical quadruplicate. **D** Sequences of HIR-6 truncations and mutants evaluated in the truncation study. Source data are provided as a Source Data file.

The previous examples of nanomolar HIR aptamers have been reported to enhance or modulate HIR autophosphorylation activity^38–40^. Therefore, we tested HIR-6 for its ability to bind and modulate the native form of HIR. We performed a binding competition assay with radiolabeled [^125^I]monoiodotyrosyl-A14-insulin using human IM-9 lymphocytes expressing HIR-A isoform and increasing concentrations of HIR-6 and HIR_SC as control. Counts per minute (CPM) were measured to determine changes in insulin binding and to calculate the inhibition constant (K_i_). Increasing concentrations of HIR-6 reduced the binding of the radiolabeled insulin, with an estimated K_i_ of 68.8 nM (Fig. 3A). The control sequence HIR_SC did not force insulin to dissociate within the tested concentration range. Although HIR-6 showed a double-digit nanomolar K_i_ in this assay, this still clearly underlines the capability of the aptamer to bind the native form of HIR even under specific buffer conditions optimized for insulin binding. Following the ability of HIR-6 to compete with insulin binding, we also tested its effect on the activation of HIR. HIR-6 did not stimulate autophosphorylation of the receptor (Supplementary Figs. 39–40), but inhibited insulin-stimulated autophosphorylation of HIR. The inhibition was expressed as a decrease in relative stimulation, providing evidence of antagonistic effect with an IC_50_ of 128 nM (Fig. 3B). This shows that the SRS method identified an aptamer with a reverse effect compared to most of the previous HIR aptamers^38–40^.

### HIR-6 binding affinity is highly influenced by its (hyper)modified sequence

Aptamers obtained by conventional selections are usually up to 100 nucleotides long. However, not all nucleotide positions are essential for target recognition, and some may cause steric hindrance, reducing the binding potential of a shorter functional motif^47^. Although HIR-6 is already relatively short, we performed a truncation study to investigate the impact of HIR-6 length and aromatic modifications on its binding performance. We systematically removed several nucleotides from one or the other sequence end by combining differently long templates and primers (Fig. 3D) using our recently reported methodology^48^ (Supplementary Figs. 32, 33). Truncating the 3’-end nucleotides resulted in decreased fluorescence intensities when 5, 10 and 15 nucleotides were removed for HIR-6_T1, HIR-6_T2 and HIR-6_T3, respectively (Fig. 3C). Interestingly, removing most of the unmodified 5’-end region did not affect the affinity, with HIR-6_T4, HIR-6_T5 and HIR-6_T6 showing comparable fluorescence despite deletions of 5, 10 and 14 bases at the 5’-end, respectively. Further sequences truncated from both ends showed lower but still significant affinity. As previously shown by cluster analysis and initial candidate screening, the core UUUAACGUAANA motif shared in cluster 1 appears essential for interaction with HIR. We prepared two mutant sequences^48^ to investigate the importance of modified nucleotides at the central (HIR-6_M1) and 3´-end (HIR-6_M2) regions. Results showed that HIR-6_M1 completely lost its binding affinity, while HIR-6_M2 was still a moderate binder. This shows that the modifications within the central region are crucial for binding affinity. Removing modifications from the 3’-end decreased affinity comparably to HIR-6_T3, indicating that either deleting the entire region or substituting the modified positions produces nearly the same effect. Overall, truncation and mutation analyses of HIR-6 highlight the importance of the 15nt hypermodified central region, with additional contributions from the modified 3’-end constant region, similar to how aptamers extend their effective length by introducing the primer sequences to form longer, more complex structures^29,49–51^.

### Covariation analysis predicted a stem-loop aptamer structure

**Fig. 4:**
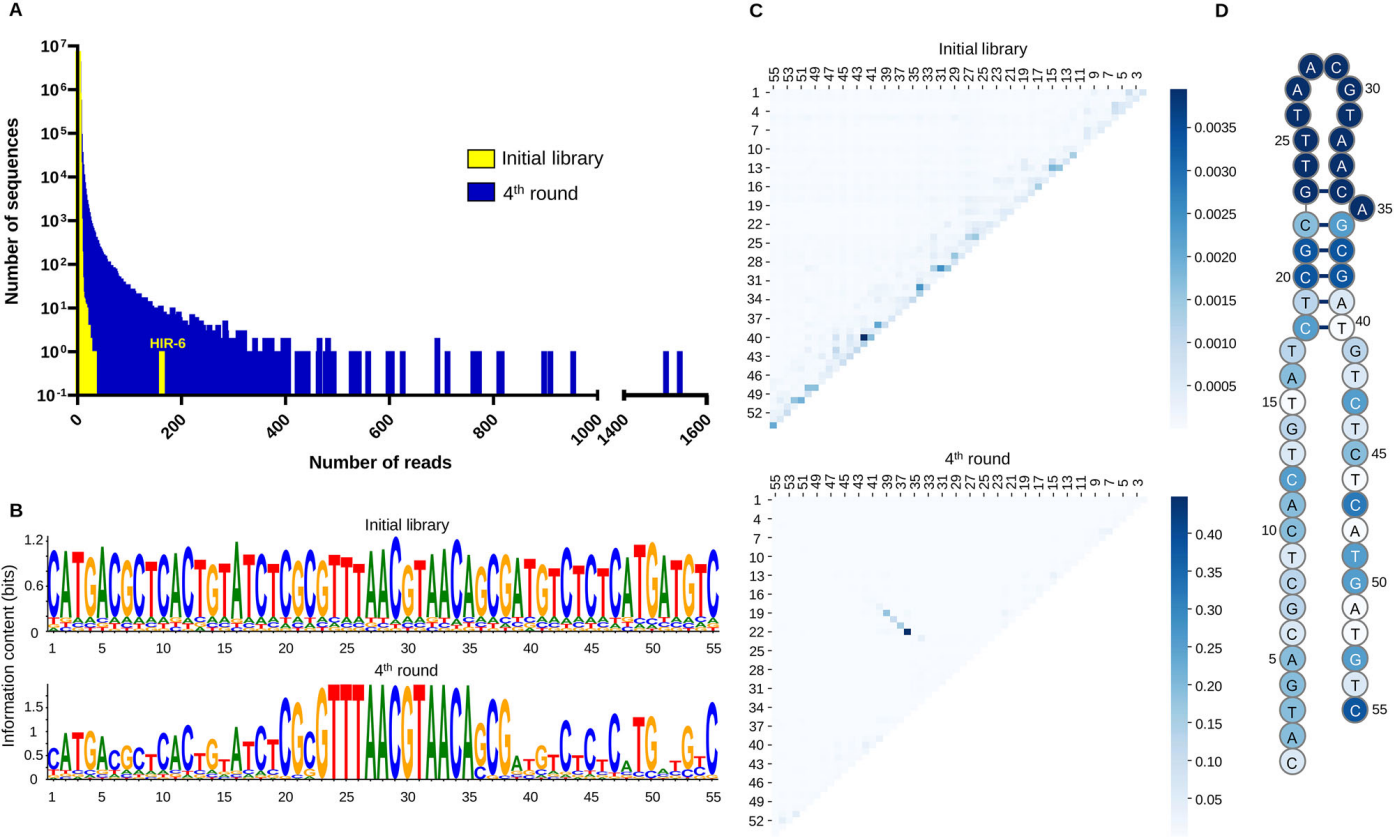
Covariation analysis. **A** Distributions of sequence abundances of the initial modified L4 library and the 4th round of selection used for covariation analysis. **B** Sequence logos of the initial modified L4 library and 4th round of selection. **C** Mutual information heatmaps for the initial modified library and the 4th round of selection. **D** Model of HIR-6 secondary structure resulting from covariation analysis. Source data are provided as a Source Data file.

Covariation analysis is often used to identify evolutionary constraint patterns that are linked to structure and function^52^ and applied to nucleic acid selections to determine multiple secondary structures^9,10,53^. For this purpose, we performed multiple rounds of selection using a library (L4) consisting of 21%-mutagenized HIR-6 sequence (Fig. 4B, top) under increasing stringency conditions (Supplementary Table 4). Results from the 4^th^ selection round showed a significant difference in the distributions of sequence abundances compared to initial library (Fig. 4A), however, the core motif remains highly conserved (Fig. 4B, bottom). This clearly indicates that mutations during the selection would lead to the loss of binding affinity, similarly as with HIR-6_M1 (Fig. 3C). Correlations with space-neighboring bases were analyzed to determine if they are coevolving during the selection process and to identify the most likely formed base pairs. Heatmaps of pairwise mutual information values before and after selection show a clear covariation pattern emerging as the consequence of selection (Fig. 4C). Nucleotide positions that exhibited strong correlations indicated the coevolution of a stem between positions 18-22 and 40-36 and formation of a loop structure containing the TTTAACGTAA motif and one-nucleotide A-bulge, followed by constant primer regions with no mutual information (Fig. 4D).

### Determination of cryo-EM structure revealed that the phenyl and indole moieties stabilize the aptamer structure and promote hydrophobic interactions with HIR

**Fig. 5:**
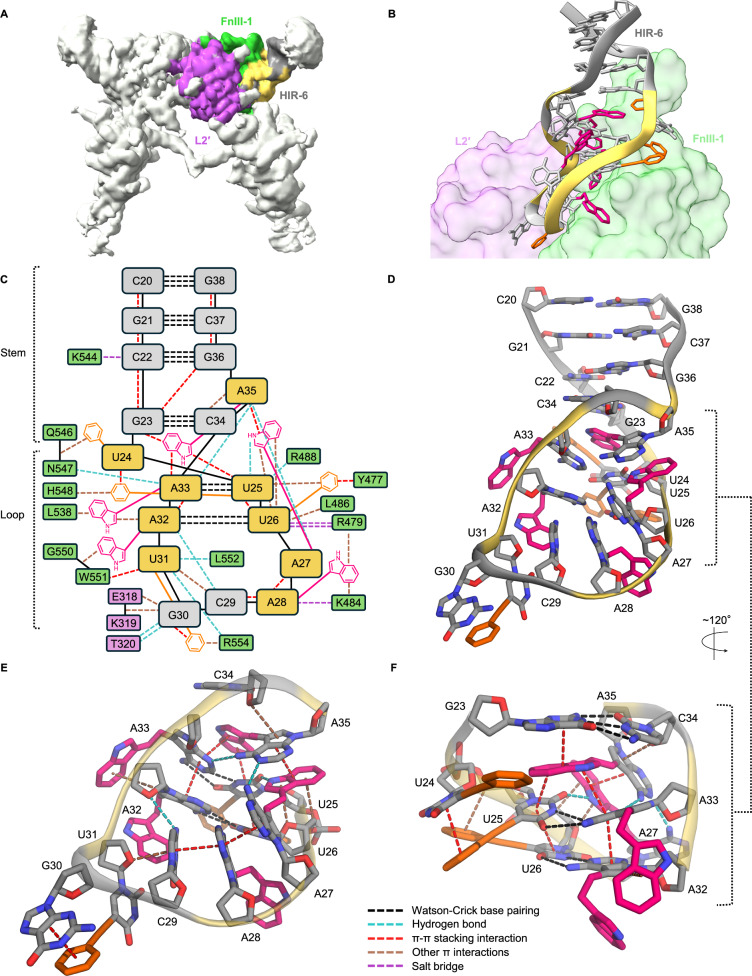
HIR-6 binding site and structure overview. **A** Overall low-resolution cryo-EM reconstruction highlighting one HIR-6 binding site on HIR ectodomain. FnIII-1 domain, L2’ domain and HIR-6 aptamer are shown in green, purple and gray, respectively, with modified nucleotides highlighted in yellow. **B** Cryo-EM (2.9 Å resolution, PDB: 9SA8) structure of the FnIII-1 and L2’ domains in complex with HIR-6 with indole and phenyl moieties highlighted in magenta and orange, respectively. The backbone cartoon of modified nucleotides is highlighted in yellow. **C** Schematic 2D diagram of HIR-6 aptamer interactions. The legend describing bond types appears in section (**F**). **D** Detailed 3D view of the HIR-6 stem-loop structure. **E** Internal interaction network in HIR-6 aptamer loop bottom (**E**) and middle (**F**) regions.

In order to gain structural insight into the aptamer-HIR complex, we determined the structure using cryo-EM. HIR forms a stable disulfide-linked homodimer (αβ)_2_ structure, and in the absence of insulin, the receptor adopts an autoinhibited Λ-shaped conformation^37^. Low-resolution cryo-EM reconstruction of the HIR-6 bound to HIR ectodomain revealed an apo-like conformation of the receptor with two equivalent molecules of HIR-6 aptamers bound to the FnIII-1 and L2 domains from opposite HIR protomers, i.e. FnIII-1 and L2’ and FnIII-1’ and L2, respectively (Fig. 5A). We then focused the cryo-EM analysis (Supplementary Figs. 42, 43 and Supplementary Table 13) on the HIR-6 binding site and reconstructed at 2.9 Å resolution (EMD-54689) the complex comprising 19 nucleotides of HIR-6 (positions C20-G38), the FnIII-1 domain (residues 469-519 and 527-573 and 578-592) and the L2’ domain (residues 308-468) (Fig. 5B, PDB: 9SA8, MolProbity score 1.92, EMRigner score 4.46).

The structural analysis revealed that the HIR-6 aptamer adopts a stem-loop motif with a non-modified G-C stem formed by four canonical Watson-Crick base pairs, serving as a scaffold for a highly modified loop (Fig. 5C, D). Beyond the G-C stem, there is only a blurred density for the T19-A39 base pair predicted by the covariation analysis (Fig. 4D), and the rest of HIR-6 is not defined in the cryo-EM reconstruction. The highly modified loop shape is stabilized by multiple intramolecular interactions involving nucleotides as well as the modifications. These include also unconventional base-base and sugar-base H-bonds, T-shape π-π stacking, and interactions of π electrons with ribose lone electron pair or with C-H groups (Fig. 5C, E, and F, Supplementary Table 14). The highly modified loop contains two unmodified bases (C29 and G30) and eight modified bases, facilitating several stacking interactions contributing to its stability. The indole modifications on A27 and on the bulged nucleotide A35 enable the formation of a cluster of π-π and other non-conventional stacking interactions. In particular, the A35 indole is inserted between the nucleobases of G23, U25, and A33, thereby extending the interaction network and reinforcing the loop structure (Fig. 5E, F). Moreover, the indole and nucleobase of A27 are engaged in a network of non-Watson-Crick H-bonds, which involves nucleobases of U25, A35, and A33, further supporting the internal structure. Stacking interactions between rigidly attached phenyl groups and nucleobases (U31-G30, U25-U24) or a CH-π interaction with the sugar-phosphate backbone (U26-U25) are observed protruding outside the loop structure. Taken together, some of the flexibly attached indoles contribute to the interaction network in the loop interior, while the phenyls attached via the rigid linker are externally projected and stabilize the structure from the outside. Aside from A27 and A35, which are key for internal interactions, all remaining aptamer positions contact the FnIII-1 and L2’ domains.

**Fig. 6:**
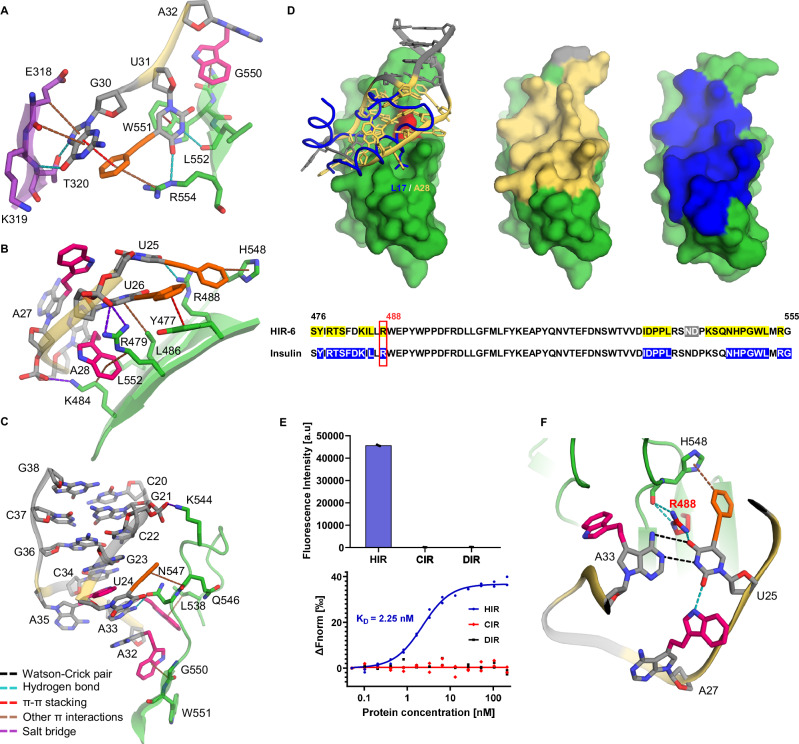
HIR-6 interaction with HIR. **A**–**C** Details of HIR-6 (gray) interactions with FnIII-1 (green) and L2’ domain (purple). The backbone cartoon of modified nucleotides is highlighted in yellow and phenyl and indole moieties are displayed in orange and magenta, respectively. The legend describing bond types appears in section C. **D** Left: Superposition of HIR-6 aptamer bound to the FnIII-1 domain with HIR-bound insulin (PDB: 6PXV^54^). Residue R488 is highlighted in red. Indole A28 of HIR-6 and insulin residue L17 occupying the same binding pocket are shown. Middle: HIR-6 binding interface. Right: Insulin binding interface. **E** HIR-6 specificity to HIR compared with orthologous proteins from cat (CIR) and dog (DIR). Binding affinity of HIR-6 was measured using FNBA assay (top, *n* = 2, each performed as technical triplicate) and microscale thermophoresis (bottom, *n* = 2 independent experiments). **F** A detail of the interaction network around R488. Source data are provided as a Source Data file.

The hypermodified aptamer loop provides a large interaction surface that mediates nearly all contacts with the receptor domains. The binding of HIR-6 to HIR creates a buried surface area of ~2434 Å^2^ and includes a broad repertoire of interactions. The hydrophobic aromatic modifications in HIR-6 provide sites for hydrophobic and π interactions. All phenyl modifications and all indole modifications except for A27 and A35, which are engaged in internal aptamer loop stabilization, form π interactions with the protein. These include regular and T-shape π-π stacking, and other interactions of π electrons with peptide bonds, C-H or cation-π interactions. Aptamer nucleobases interact with HIR through several H-bonds, and salt bridges are formed between phosphate moieties and positively charged residues (Fig. 5C and 6A, B and C, and Supplementary Table 15).

A key local interaction network is centered around the phenyl modification of U31, which mediates an inter-domain array of stacking interactions bridging the FnIII-1 domain, HIR-6, and L2’ domain (Fig. 6A). The phenyl moiety is sandwiched between R554 from FnIII-1 domain (cation-π stacking) and the nucleobase of G30, which itself stacks on the other face with the L2’ domain aliphatic chain of E318 as well as the E318-K319 peptide bond. These stacking interactions are further supported by H-bond interactions of nucleobases of G30 and U31. Specifically, G30 interacts with L2’ T320 main chain and side chain, while U31 interacts with FnIII-1 R554 side chain and L552 main chain, and additionally stacks with the aromatic W551. The bridging of the FnIII and L2’ domains by the aptamer locks HIR in the apo-like autoinhibited Λ-shaped state and thus prevents the domain rearrangement induced by insulin binding to site 2^37^.

Another notable example of a local interaction network between HIR-6 and HIR is the binding of A28, which inserts the indole modification into a hydrophobic pocket formed by residues R479, K484, L486 and L552 (Fig. 6B). This hydrophobic cavity in insulin binding site 2 serves as the binding site for insulin residue L17 (Fig. 6D). The indole stacks between the aliphatic chains of R479 and K484, that are also stabilized by salt bridges to phosphates moieties of A27 and U26.

### HIR-6 aptamer binds to insulin-binding site 2 with exquisite specificity

The HIR-6 aptamer binding site significantly overlaps with the insulin binding site 2, located on the surface of a major β sheet of FnIII-1 domain^54^. Insulin binding to this site promotes the formation of activated states of the receptor in which mutations have shown a negative effect on HIR autophosphorylation^37^. About 86% of HIR residues interacting with insulin site 2 lie within the HIR-6 binding interface (Fig. 6D). The interaction network between HIR-6 and the FnIII-1 and L2’ domains (Fig. 6A) stabilizes the auto-inhibited conformation of the receptor (Fig. 5A, B). This structural information, together with the HIR-6 competition and inhibition assays (Fig. 3A, B) indicates that the HIR-6 compete for site 2 and the resulting stabilization of the inactive receptor state represent the molecular mechanism of insulin antagonism effect of HIR-6.

We have also further investigated the specificity of HIR-6 by testing its binding affinity to highly related dog (DIR) and cat (CIR) insulin receptors with ≥96% amino acid sequence identity compared to HIR (Supplementary Figs. 4–5). Interestingly, HIR-6 showed no binding to DIR and CIR receptors (Fig. 6E), exhibiting remarkable specificity. Such a high level of specificity has been previously reported only for very few examples of aptamers obtained from multi-round^4^ selections typically including counter-SELEX against the competing targets^55,56^. Structure analysis showed there is only one amino acid difference (R488K) between human and orthologous DIR and CIR proteins within the HIR-6 binding interface (Fig. 6D). The guanidyl moiety of R488 is involved in three hydrogen bonds with HIR-6: two H-bonds are formed with the peptide bond oxygen of H548 and optimally position the third guanidyl nitrogen to form the third H-bond with the carbonyl group of uracil U25 (Fig. 6F). Given the fewer possibilities for hydrogen bonding of a lysine amino group and the difference in side-chain length, it is less likely that K488 would simultaneously hydrogen bond with both H548 and U25. Since a previous report suggested that a single hydrogen bond from cubane-linked aptamer^19^ might cause such a level of target specificity, the pattern of H-bonds around R488 could potentially contribute to the high specificity towards HIR. However, we cannot rule out the possibility that other amino acid mutations outside the HIR-6 interface may contribute to other structural differences underlying the specificity.

### Scope of the SRS - selection of an aptamer for the related DIR target

To demonstrate that our SRS approach can generate aptamers beyond HIR, we have performed another SRS targeting DIR. From the enriched sequences (Supplementary Fig. 18), we identified and synthesized five aptamer candidates as representative examples of the top clusters and tested them by FNBA (Supplementary Figs. 29, 30 and 35 and Table 11). Based on this screening, DIR-5 aptamer was further validated by MST, exhibiting a K_D_ of 19.2 nM for DIR. This aptamer has shown ca. 4-fold specificity for DIR, while binding HIR with a K_D_ of 74.8 nM (Supplementary Fig. 37). Although the half-order-of-magnitude specificity is rather moderate (compared to HIR-6), it is comparable to several previously reported modified aptamers obtained from multi-round SELEX^16^ that showed a similar level of cross-species reactivity to 96% amino acid sequence-identity proteins. Nevertheless, cross-species reactivity can be useful for identifying ligands whose efficacy can be tested in animal models^57^. These results show that the SRS approach can be used for rapid identification of aptamers against other proteins and generate specific aptamers suitable for several applications.

## Discussion

Previously, over-represented libraries with limited structural diversity have mostly produced poorly performing functional nucleic acid molecules, such as moderately active deoxyribozymes^8^ or weakly specific methyl-mannoside aptamers^31^. Very few scattered examples of aptamers selected from a short sequence space showed binding affinities comparable to those from conventional selections, i.e. re-identified thrombin aptamers^31^ and interleukin-6 aptamers from a library predicted to maintain a high level of secondary structures^30^. This apparent low success rate suggests that the properties of canonical over-represented libraries have limited capacity in aptamer discovery and use in single-round methods. In this work, we present an approach that allows aptamer selection in a single round by combining over-represented chemically expanded libraries featuring two different hydrophobic modified nucleotides, consecutive partition steps, and sequence abundance distribution analysis paired with clustering. In this approach, sequence over-representation driven by the length of the randomized (N) region plays a significant role. N13 to N17 showed a good balance between the number of over-represented sequences and length, capable of forming various secondary structures. Sequence over-representation coming from N19 and longer libraries was insufficient to generate non-exponential enrichment suitable for distribution and clustering analyses, making the eluted sequences unable to rank for affinity screening. We demonstrated that changes in the over-representation of N13, N15 and N17 dually modified libraries produce easily identifiable clusters of sequences. By forming distinct clusters, the need for extensive screening of aptamer candidates is reduced to testing only a few sequences, thus facilitating aptamer discovery. These changes in the modified aptamer landscapes revealed that short, dually modified, over-represented libraries offer significant advantages over standard DNA libraries when two consecutive partition steps are applied. Moreover, incorporating the modified nucleotides also benefits from shorter libraries requiring less extensive enzymatic^58^ or chemical^59^ synthesis optimizations and faster truncation analysis^60^, highlighting the synergistic effect of these components. Additionally, single-round selections provide further benefits, such as excellent compatibility of base-modified dNTPs in PEX since many bulkier derivatives are often not suitable as substrates for PCR. Based on these advantages, the SRS approach offers the expedient ability to screen multiple differently-modified libraries, apply various selection conditions, target several molecules, all in parallel and within a short period of time.

The single-round effectiveness in producing high-quality aptamers was demonstrated by HIR-6, which exhibited picomolar binding affinity to HIR, thus outperforming many aptamers from conventional selections and SRSs using over-represented libraries. The identification of HIR-6 as a top over-represented sequence in one of the most representative clusters validates the straightforward screening process. Furthermore, the ability of HIR-6 to discriminate subtle amino acid variations highlights the potential of the method to generate highly functional, specific aptamers through amino acid side chain-like interactions with both phenyl and indole moieties, along with flexible and rigid linkers. Moreover, the library with fifteen randomized positions, synthesized with two modified nucleotides, resulted in the selection of a hypermodified loop containing only two unmodified bases. Furthermore, it enabled us to experimentally test the whole sequence space and to identify the UUUAACGUAA motif containing 8 modified nucleotides from four independent selections, further validated as the core of the aptamer across the truncation analysis, co-variation study and cryo-EM structural analysis. To demonstrate the scope of the SRS approach, we performed another selection and identified aptamers against DIR with double-digit nanomolar K_D_ and 4-fold specificity. It shows the generality of the SRS methodology and its applicability for other targets. However, we assume that, in other cases, screening of other types of libraries containing different nucleotide analogs^42,61–63^ might be needed to identify the best-performing combination and unique target-complementary adoption. Moreover, to the best of our knowledge, this is the first performance comparison between dually modified and unmodified over-represented libraries with an unconventional short sequence space for aptamer identification using a single-round selection.

To the best of our knowledge, this is only the third reported example of dually modified aptamers containing two different modified nucleotides – but unlike the previous examples of dually modified SOMAmers^16^, and the recently reported dually modified aptamer-drug conjugate selected by multi-round baited-SELEX^17^, we utilized not only two different modifications but also two different linkers - one rigid alkyne and one flexible alkyl. This combination of hydrophobic aromatic groups and different linkers is unique and seems to be of crucial importance for the high binding affinity of the aptamer through adopting an exceptionally target-complementary shape as well as through specific interactions. While the flexible indole was highly involved in stabilizing the internal loop structure, the rigidity of the phenyls was needed to stabilize the structure from the outside, but both moieties are involved in essential interactions with the protein surface. None of these could be achieved with unmodified DNA library of the same sequence diversity.

The previously reported examples^38–41^ of modified aptamers targeting HIR have been selected using N40 libraries containing 5-[N-(1-naphthylmethyl) carboxamide]−2′-deoxyuridine via 8 rounds of conventional SELEX. These naphthyl-modified aptamers (A43, A48 and A62) exhibited nanomolar K_D_s and high specificity between HIR and IGF-1 R in addition to their allosteric activity as positive and/or negative modulators^38–40^. In our work, we demonstrated that the use of dually modified N17 and shorter random-region libraries in the SRS approach compensated for the lower-sequence diversity and generated HIR aptamers that even outperformed the previously reported aptamers showing low-nanomolar K_D_s and a very high level of IGF-1 R cross-target specificity.

Successful solving of the cryo-EM structure of HIR-6 aptamer bound to HIR enabled comparison with previously reported^41^ structures of A62 and A43 aptamers in complex with HIR (Supplementary Fig. 44). Aptamer A62 binds to both the FnIII-1 and L1′ domains and can induce two conformations of the HIR: symmetric binding of two A62 aptamers induces an arrowhead activated state, while binding of one A62 molecule together with one molecule of insulin results in a titled-T activated state. Aptamer A43 stabilizes the one-insulin-bound Γ-shaped fully activated state by bridging the FnIII-1 domain with CR′ and L2′. In contrast, symmetric binding of two HIR-6 aptamers stabilizes the apo-like Λ-shaped inactive state in a similar way as antibody Fab 83-14^64,65^. Indeed, both Fab 83-14^66^ and HIR-6 binding show an antagonistic effect on HIR, inhibiting insulin binding and HIR autophosphorylation. Although hyperinsulinism is less common than other insulin receptor-associated diseases^67^, such highly potent and specific aptamer with insulin antagonistic activity could be considered as a potential candidate for therapeutic applications.

It is important to note that the SRS approach has some limitations due to its non-exponential enrichment and lower sequence diversity. In some cases, the SRS for certain targets may not yield enriched sequences required to form distinguishable clusters or directly achieve exquisite aptamer specificity without further library optimization. However, non-exponential enrichment may be increased by enhancing partition efficiency with the PEG-based hydrogel^28^ matrix, as shown for conventional unmodified libraries. Therefore, a combination of these two technological advances can be further beneficial for SRSs. Additionally, since the SRS is very rapid and straightforward, one can very quickly test several different libraries featuring different types of secondary structures and different modifications and thus increase the chance of identification of potent binders.

In conclusion, we have developed a single-round selection strategy which enables rapid screening of multiple libraries and selection conditions, and provides high-affinity aptamers without the need for iterative selection cycles. Since diverse targets may need different libraries containing different modifications, this approach can quickly pre-screen them to identify the best-performing library, thereby increasing the success rate of the selection process. In accord with previous works on base-modified aptamers^14–20^, our results demonstrate that a combination of two different aromatic hydrophobic moieties attached to nucleotides can significantly expand secondary structure diversity as well as chemical diversity, bringing additional types of interactions with protein targets. This combination is essential for larger surface contacts with the targets (in particular with proteins), resulting in high binding affinity and specificity. The cryo-EM structure suggests that a balanced ratio of one rigid and one flexible linker connecting the moieties to the nucleobase provides an effective solution for stabilizing the aptamer secondary structure, positioning the moieties within shallow protein surfaces, and enabling the enzymatic production of hypermodified aptamers. It seems that a combination of two conventional and two modified nucleotides is sufficient for the formation of a hypermodified central aptamer region, with each base-modified nucleotide analog showing its own significance. Modified libraries with N13-17 randomized region lengths are sufficiently over-represented, with thousands to millions of copies of each individual sequence. This makes them suitable for rapid single-round selection strategies. Such over-representation provides power in NGS data analysis, where the ranking and clustering of eluted sequences prioritize their testing and avoid the need for extensive candidate screening. Although larger libraries undergoing multi-round SELEX may exhibit more complex secondary structures, our study shows that incorporating two nucleotide analogs within short motifs can compensate for the structural diversity of conventional libraries and deliver the required binding properties. Our approach addresses the main drawbacks of selection: lack of chemical functionalities, speed and scalability of the process. Alongside its high-throughput potential, this can accelerate the development of next-gen nucleic acid functional motifs for use in diagnostics or therapeutics.

## Methods

### General remarks

Sequences of natural templates, primers and candidates were purchased from Generi Biotech (Czech Republic). Randomized DNA pools were obtained from Integrated DNA Technologies (IDT, USA). Natural nucleoside triphosphates (dATP, dTTP, dCTP, dGTP), Dynabeads MyOne Streptavidin C1, Dynabeads His-Tag Isolation & Pulldown, Human Serum Albumin (HSA), Prothrombin, 1-ethyl-3-(3-dimethylaminopropyl) carbodiimide hydrochloride (EDC), Sulfo-NHS (N-hydroxysulfosuccinimide) and Dextrane Sulfate sodium salt from Leuconostoc spp. were purchased from ThermoFisher Scientific. KOD XL DNA polymerase was purchased from Merck (Sigma Aldrich). Primer Extension (PEX) and Polymerase chain reaction (PCR) reactions were performed in VWR thermal cycler (Doppio), Quantitative Polymerase chain reactions (qPCR) with CFX96 Real-time System thermal cycler (Bio-Rad). When required, PEX or PCR products were purified using the Monarch PCR & DNA Purification Kit (New England Biolabs, NEB) and QIAquick PCR Purification Kit (Qiagen). Purified product concentrations were measured by UV-Vis spectra at room temperature (r.t.) on Nanophotometer N60 (IMPLEN) and concentrated on CentriVap Vacuum Concentrator system (Labconco). Samples from PEX reactions were separated by 12.5% polyacrylamide gel electrophoresis (PAGE, acrylamide/bisacrylamide 19:1,25% urea [v/v]) with 1X TBE buffer (420 mA, 1 h) using stop solution (95% [v/v] formamide, 0.5 mM EDTA, 0.025% [w/v] bromophenol blue, 0.025% [w/v] xylene cyanol FF, 0.025% [w/v] SDS in Milli-Q water). PEX, PCR reactions, λ exonuclease digestions, and single-strand separations were separated by native 3% agarose gel (Serva) electrophoresis with 0.5X TBE buffer (120 V, 70 min) using 6X DNA Gel Loading Dye (ThermoFisher Scientific). All gels were analyzed by GelRed or fluorescence imaging using Typhoon FLA 9500 (GE Healthcare Life Sciences) by comparing the migration to a custom-made FAM-/Cy5-labeled DNA ladder or Low Range DNA Ladder (Invitrogen). His-Tagged Human Insulin Receptor (HIR, His28-Lys944, Cat. No. INR-H52Ha), His-Tagged Human IGF-1 R (Glu31-Asn932, Cat. No. IGR-H5229), His-Tagged Cat Insulin Receptor (CIR, His28-Ile956, Cat. No. INR-C52H3), Dog Insulin Receptor (DIR, His28-Ile956, Cat. No. INR-D52H3) and Capsid protein p24 (HIV, Cat. No. CP4-H52H3) were acquired from ACROBiosystems. His-Tagged CPA protein was prepared in-house by Milan Kožíšek (Jan Konvalinka research group, IOCB)^68^. His-tagged peptide (HHHHHH) was custom-made in-house. Dulbecco´s PBS buffer containing 1.5 mM KH_2_PO_4_, 8.1 mM Na_2_HPO_4_, 2.7 mM KCl, 137 mM NaCl was purchased from BioConcept.

### Enzymatic synthesis - Incorporation of two modified nucleotides

Doubly-modified DNA libraries (L2, L2’, L3, L4 and L5) used for selections were synthesized by PEX reaction in a 20 µL total volume containing 0.4 mM dNTPs (dA^R^TP, dU^R^TP, dCTP, dGTP; for combinations see Supplementary Table 2; 0.6 mM for L4; for chemical synthesis of modified nucleotides see Supplementary Section 2), 12.5 µM 5’-biotinylated library template (Supplementary Table 1), 10 µM 5’-Cy5-labeled primer (Supplementary Table 1), 1X KOD XL buffer and 2.5 U of KOD XL DNA polymerase. The reaction was incubated for 3 min at 95 °C, 2 min at 55 °C, and overnight at 60 °C. The unmodified library L1 was prepared using the same protocol but with all four natural dNTPs (dATP, dTTP, dCTP and dGTP). These PEX conditions (Supplementary Table 12) were also applied for enzymatic syntheses of all modified oligonucleotides used in this study (Supplementary Table 2). Full-length products were confirmed by LC-MS (Supplementary Figs. 52–72). PEX reactions were purified by HPLC using a C18 column (Waters XBridge Premiere BEH Oligo 4.6 × 150 mm) heated to 60 °C, with a linear gradient from 0.1 M TEAB in 5% MeCN in water to 0.1 M TEAB in 20% MeCN in water over one h, followed by a transition to 100% MeCN in one h. Buffer pH was adjusted to 7.4 with CO_2_ (g). HPLC fractions were freeze-dried, reconstituted in water, and quantified using a Nanophotometer N60 with Cy5 detection wavelength.

### Protein immobilization

HIR or DIR were immobilized on Dynabeads His-Tag Isolation & Pulldown beads: 10 µL of resuspended beads were collected on a magnet to remove the supernatant and incubated with His-tagged protein (24 µg, 150% of theoretical beads capacity) and His-tagged peptide (4 µg, 25% of theoretical beads capacity, used as a surface blocker) in 500 µL of binding buffer (50 mM sodium phosphate, 300 mM NaCl, 0.01% Tween-20, pH 8) for 30 min at r.t. using a Hulamixer. Beads were washed twice with 400 µL of binding buffer, once with 400 µL of PBS and then collected on a magnet. Supernatant was removed and HIR-/DIR-functionalized beads were resuspended in 10 µL of PBS. The immobilized protein was quantified with the BCA Protein Assay Kit (Novagen): A series of BSA dilutions (20-2000 µg/mL) was used to create a linear calibration plot within the expected HIR or DIR concentration range. Beads functionalized only with His-tagged peptide, HIR-/DIR-functionalized beads and BSA dilutions were mixed with BCA working reagent (1:20) and incubated for three hours at r.t. using a Hulamixer. Absorbance was measured at 562 nm with the Nanophotometer N60, and the concentration was determined from the calibration curve equation.

### Single-round aptamer selection

Aptamer selections involved a single round with two partitioning steps (Supplementary Table 3). The first step consisted of a mixture containing 300 pmol of pre-folded (incubated 3 min at 95 °C and 10 min at r.t.) library (L1, L2, L2’, L3 or L5), 1000 nM HIR-/DIR-functionalized magnetic beads, 1% HSA and 1 µM Prothrombin. This interaction mixture was incubated at r.t. for 30 min in interaction buffer (IB, PBS, 5 mM MgCl_2_, pH 7.4). Non-binding sequences were removed by three washing steps (300 µL of IB for 4 min). Binding sequences were then thermally eluted into water at 95 °C with shaking at 1000 rpm for 5 min. The second partitioning step consisted of a mixture containing the eluted binding sequences and 150 nM HIR-/DIR-functionalized magnetic beads in IB. This mixture was incubated for 30 min at r.t., followed by 15 min of incubation with 800 µL of 3 mM DxSO_4_ in IB. Non-binding sequences were removed by five washing steps (300 µL of IB for 4 min) and the binding sequences were again thermally eluted. The optimal number of PCR cycles was determined by qPCR and verified by native agarose gel electrophoresis.

### Optimal PCR cycle determination

Eluted sequences were amplified in a 20 µL qPCR reaction mixture containing 0.4 µM forward and reverse adapter primers (Supplementary Table 1) and 1X SsoAdvance Universal SYBR Green Supermix (Bio-Rad). The reaction mixture was incubated at 95 °C for 2 minutes, followed by 30 cycles at 95 °C for 15 sec and 60 °C for 30 sec. The optimal amplification cycles were determined when a relatively high level of fluorescence was recorded.

### Selection for covariation analysis

Selection for covariation analysis consisted of four rounds. The first incubation mixture contained 200 pmol of pre-folded (3 min at 90 °C and 10 min at r.t.) L4 library, 100 nM HIR-functionalized beads, 1% HSA and 1 µM Prothrombin. The interaction mixture was incubated at r.t. for 30 min in IB, followed by 15 min of incubation with 800 µL of 3 mM DxSO_4_ in IB. Non-binding sequences were removed by 5 washing steps (300 µL of IB for 4 min). Binding sequences were thermally eluted into water at 95 °C with shaking at 1000 rpm for 5 min. The optimal number of PCR cycles was determined by qPCR and verified by native agarose gel electrophoresis. During subsequent cycles, selection stringency was increased (Supplementary Table 4).

### Generation of single-stranded DNA using an exonuclease

Eluted sequences from the covariation analysis were PCR-amplified to obtain natural dsDNA. The 20 µL PCR mixture contained 5 µL of eluted sequences, 0.15 mM dNTPs, 0.4 µM 5’-biotinylated Fw3 primer (Supplementary Table 1), 0.4 µM 5’-phosphorylated Rev3 primer (Supplementary Table 1), 1X KOD XL buffer and 1.25 U of KOD XL DNA polymerase. The reaction mixture was incubated at 95 °C for 3 minutes, followed by the qPCR-determined number of cycles (95 °C for 30 seconds, 60 °C for 30 seconds, and 72 °C for 30 seconds). If needed, additional PCR reactions were performed using the described conditions with 1 pmol of dsDNA as template. Products were purified using the Monarch PCR & DNA purification kit (NEB), quantified, and verified by native agarose gel electrophoresis. The purified dsDNA was then used for λ-exonuclease digestion of the 5’-phosphorylated reverse strand. A 50 µL digestion reaction consisted of 1X λ-exonuclease buffer, 10 U of λ-exonuclease, and 70 pmol of dsDNA, and was incubated at 37 °C and 550 rpm shaking for 2.5 h. The 5’-biotinylated forward strand (which will later serve as template for PEX reactions with modified dN^R^TPs) was purified with the Monarch PCR & DNA purification kit and verified by native agarose gel electrophoresis.

### Generation of single-stranded DNA using magnetic beads

The PEX reactions were strand-separated using streptavidin magnetic beads (SMB, Dynabeads MyOne Streptavidin C1): 200 µL of SMB were washed three times with 400 µL of washing buffer (5 mM Tris-HCl, 0.5 mM EDTA, 1 M NaCl, pH 7.5). PEX reaction mixture (20 µL) was mixed with 380 µL nuclease-free water and 400 µL binding buffer (10 mM Tris-HCl, 1 mM EDTA, 2 M NaCl, pH 7.5) and incubated with washed SMB for 60 min at r.t. using a Hulamixer. Immobilized dsDNA was washed three times with 400 µL of washing buffer and then incubated with 100 µL of 20 mM NaOH. The SMB were collected on a magnet, and the solution containing the (modified) strand was transferred into a new vial, neutralized with 0.5 M HCl, and buffered with PBS. Samples were centrifuged at 13,000 × *g* for 3 min to remove any remaining SMB, quantified using a Nanophotometer N60 and analyzed by native 3% agarose.

### Next-generation sequencing (NGS)

Recovered binding sequences from aptamer selections were prepared for NGS by two additional PCR reactions – adapter and index PCR (Supplementary Figs. 6–9). Three adapter PCR reactions (20 µL each) contained 5 µL of eluted sequences, 0.15 mM dNTPs, 0.4 µM forward and reverse adapter primers (Supplementary Table 1), 1X KOD XL buffer, 1.25 U of KOD XL DNA polymerase and were amplified using the following thermal cycling protocol: 95 °C for 3 min followed by 10–18 cycles at 95 °C for 30 sec, 60 °C for 30 sec and 72 °C for 30 sec. PCR products were purified using the Monarch PCR & DNA purification kit and used as templates for subsequent index PCR. This mixture (20 µL) contained 150 fmol of dsDNA from adapter PCR, 0.15 mM dNTPs, 2 µL of illumina i5 Index, 2 µL of illumina i7 Index, 1X KOD XL buffer, 1.25 U of KOD XL DNA polymerase and was incubated at 95 °C for 3 min followed by five cycles at 95 °C for 30 sec, 60 °C for 30 sec and 72 °C for 30 sec. PCR products were extracted from a native 3% agarose gel, analyzed using a 4150 TapeStation (Agilent), and quantified with the NEBNext Ultra II DNA Library Prep Kit for Illumina, following the manufacturer´s protocol. Libraries were pooled to an equimolar ratio and paired-end sequenced using a NovaSeq system (Novogene, Cambridge). NGS data was processed with Cutadapt^69^, NGmerge^70^, Seqkit^71^, FASTX-Toolkit^72^ and FASTAptameR 2.0^43^ (Supplementary Table 5 and Equation 1).

### Fluorescent Nickel-plate binding affinity assay (FNBA)

Plate assays were performed in 96-well Pierce Nickel-coated Plates. For immobilization, 100 pmol of His-tagged proteins were incubated for 2 h at r.t. in PBS with shaking at 500 rpm. Wells were washed twice with PBS containing 0.2% Tween-20 and once with IB. Then, 100 pmol of pre-folded 5’-Cy5-labeled aptamers were incubated for 1 h at r.t. in IB. Wells were washed three times with IB and 100 µL of IB was added for measurement. Fluorescence intensity was measured at 633ex/678em nm using a Spark multimode microplate reader (Tecan).

### Microscale thermophoresis (MST)

Serial dilutions (1:1) of HIR, IGF-1 R, CIR or DIR were incubated for one h at r.t. with 20 nM 5’-Cy5-labeled aptamers in IB containing 0.1% Pluronic F-127 to produce a protein concentration range. Samples were loaded into premium MonolithX Capillaries and measured using a MonolithX (MM-286, NanoTemper Technologies) at 25 °C. Instrument parameters were adjusted to 100 % LED power and medium MST power. Dissociation constants (K_D_s) were calculated using the GraphPad Prism 8.01 software. Data were fitted to a specific binding with a Hill Slope non-linear regression model.

### Surface plasmon resonance (SPR)

For kinetic analysis, the Plasmon IV SPR platform, based on wavelength spectroscopy of surface plasmons, with six independent sensing channels and dispersionless microfluidics consisting of a polysulphon flow cell, a PVC gasket, and a thermostabilizing unit with precision to 0.1 °C, was used^73,74^. First, a glass-based sensor with a 1.5 nm Ti and 50 nm Au layer was modified with a self-assembled monolayer (SAM) of mixed OH- and COOH-terminated alkylthiols^75^. A mixture of 95 mM EDC and 25 mM NHS in miliQ water was injected for 10 min at a flow rate of 5 µL/min. A 50 µg/mL streptavidin solution in acetate buffer, pH 5, was subsequently injected onto the activated surface at a flow rate of 20 µL/min for about 20 min to reach saturation. The excess streptavidin was washed out using PBS buffer, pH 7.4, containing 750 mM NaCl, for 5 min at 20 µL/min, and unreacted COOH groups were inactivated by flowing 0.5 M Ethanolamine for 5 min at 20 µL/min. Then, 10 nM 5’-biotinylated HIR-6 aptamer (pre-folded at 90 °C for 3 min in IB) was injected onto the streptavidin-coated sensor surface for 10 min to reach a sensor response of ~0.14 nm, corresponding to ~2.4 ng/cm². HIR concentrations ranging from 60 to 5 nM were injected for 3 min into the streptavidin-HIR-6-modified sensor surface (detection channel) and the streptavidin-coated sensor surface (reference channel). All kinetic measurements were performed at 25 °C using IB as running buffer at a flow rate of 20 μL/min. A reference-compensated sensor response was obtained as a difference between the sensor response obtained in the detection and reference channels. Reference-compensated sensor responses were used for the kinetic analysis of the interactions. Kinetic and equilibrium constants were calculated assuming a 1:1 stoichiometry (1:1 Langmuir model with mass transfer) using BIAevaluation software (Biacore).

### Biolayer interferometry (BLI)

Binding kinetics were measured using an Octet RED96 (ForteBio) instrument using AR2G biosensors (Sartorius). The BLI run consisted of following steps: 60 sec hydration with water, 300 sec activation (400 mM EDC, 200 mM Sulfo-NHS), 600 sec streptavidin immobilization (500 nM, acetate buffer, pH 5.5), 300 sec quenching (1 M Ethanolamine in water), 120 sec baseline (IB), 600 sec loading of pre-folded 5’-biotinylated aptamer (100 nM, IB), 120 sec baseline (IB) and 500 sec for each association and dissociation phases. Measurements were performed at r.t. with HIR serially diluted concentrations (100 to 6.25 nM) in the association phase. One biosensor without a loaded aptamer, treated with 100 nM HIR, was used as a reference for background subtraction. Data were analyzed using GraphPad Prism 8.01 software assuming 1:1 stoichiometry.

### Receptor-binding cell studies

Human IM-9 lymphocytes (ATCC, CCL-159^TM^) were grown in RPMI 1640 medium supplemented with 10% fetal bovine serum, 2 mM L-glutamine, 100 units/mL penicillin and 100 µg/mL streptomycin. Mouse embryonic fibroblasts derived from IGF-1 R knockout mice stably transfected with human IR-A, kindly provided by A. Belfiore (Catanzaro, Italy) and R. Baserga (Philadelphia, Pennsylvania, USA), were grown in Dulbecco-modified Eagle medium (DMEM) with 5 mM glucose supplemented with 10 % fetal bovine serum, 2 mM L-glutamine, 0.3 µg/mL puromycin, 100 units/mL penicillin and 100 µg/mL streptomycin. Cells were kept in humidified air with 5% CO_2_ at 37 °C^76–78^. Binding affinities of the aptamers for IR-A were determined by the competition of compounds with [^125^I]monoiodotyrosyl-A14-insulin for IR-A in cell membranes of human IM-9 lymphocytes (ATCC). For the assay, 2.10^6^/mL cells were incubated with increasing concentrations of insulin/aptamer and human [^125^I]monoiodotyrosyl-A14-insulin (2200 Ci/mmol, 20,000 cpm, about 0.01 nM) for 2.5 h at 15 °C in HEPES binding buffer (100 mM HEPES, 100 mM NaCl, 5 mM KCl, 1.3 mM MgSO_4_, 1 mM EDTA, 10 mM Glucose, 15 mM NaOAc, 1% BSA (w/v), pH 7.6) (500 µL). After incubation, 2 × 200 µL (providing technical replicates) were centrifuged at 13,000 x g for 10 min. Radioactive pellets were counted using a Wizard 1470 Automatic γ Counter (PerkinElmer Life Sciences). Binding curves of HIR-6 and HIR_SC were measured and analyzed by GraphPad Prism 8, using a non-linear regression method, a one-site fitting program, and considering the potential depletion of free ligand that allows determination of the dissociation constant of unlabeled ligand (Supplementary Fig. 38). The dissociation constant of human [^125^I]monoiodotyrosyl-A14-insulin was set up to 0.3 nM. Radiolabeled [^125^I]monoiodotyrosyl-A14-insulin was prepared via radioiodination of Tyr14 of human insulin with ^125^I (Na[^125^I], product code: I-RB-41, 1 mCi, IZOTOP, Hungary), using the IODO-GEN system (Pierce)^79^.

### Receptor phosphorylation and antagonism assay

Ligand-dose response HIR-A autophosphorylation levels in mouse embryonic fibroblasts (IR-A) were determined using an In-Cell Western assay adapted for chemiluminescence^80^. The IR-A cells were plated at 20,000 cells/well in white 96-well Brand plates cell grade (Brand GMBH, Germany) and incubated for 24 h. The cells were starved for 4 h in serum-free media and stimulated with dilutions of ligands for 20 min. After incubation, the cells were fixed in 3.75% freshly prepared formaldehyde for 20 min. The cells were permeabilized with 0.1% Triton-X-100 in PBS for 5 min and blocked with 5% BSA in T-TBS. Plates were incubated with Phospho-IGF-I Rβ (Tyr1135/1136)/IRβ (Tyr1150/1151) (19H7) (Cell Signaling Technology, 3024) overnight at 4 °C and developed with peroxidase-labeled anti-rabbit secondary antibody (Sigma). SuperSignal West Femto maximum sensitivity substrate was added to each well, and chemiluminescence was detected using the ChemiDocMP Imaging System. For antagonism, the cells were incubated with the aptamers (concentration range from 0.1 nM to 1 μM) in the presence of 10 nM insulin. Data were subtracted from the background values and expressed as the contribution of phosphorylation relative to the 10 nM insulin signal. Nonlinear regression curve fitting of the combined data from all experiments was carried out with GraphPad Prism 8 software^81^. Control western blots (Supplementary Fig. 40) were performed using standard procedures. The IR-A cells on 24-well plates were stimulated with 500 and 100 nM HIR-6 alone and in the presence of 10 nM insulin for 20 min. Proteins were routinely analyzed using immunoblotting. The membranes were cut at 75 kDa and 50 kDa standards. The appropriate parts of the membrane were probed with Phospho-IGF-I Rβ (Tyr1135/1136)/IRβ (Tyr1150/1151) (19H7) (above 75 kDa), with Phospho-Akt (Thr308) (C31E5E) (Cell Signaling Technology, 2965) (75-50 kDa) and Akt (pan) (C67E7) (Cell Signaling Technology, 4691) used as loading controls.

### Cryo-electron microscopy (cryo-EM) of HIR-HIR-6 complex

#### Cryo-EM grid preparation

HIR was reconstituted to a concentration of 3.38 µM, dialyzed to PBS using a D-tube Dialyzer mini (Merck, MWCO 6-8 kDa), and quantified with a BCA assay. The final interaction mixture (50 µL) used to prepare the cryo-EM grids contained 2 µM HIR-6 and 0.72 µM HIR in IB. Each sample was incubated for 10 min at 4 °C. Aliquots of 3 μL were applied to a glow-discharged Quantifoil R2/1 Au 300 mesh grid, immediately blotted for 2 sec, and plunged into liquid ethane using a Thermo Fisher Scientific Vitrobot Mark IV (4 °C, 100% humidity).

#### Cryo-EM data collection

The grids were loaded into a 300 kV Titan Krios (FEI) electron microscope equipped with a Gatan K3 (model 1025) direct electron detector mounted on a Gatan BioQuantum (model 1967) energy filter. Data were collected using Serial EM software^82^ in image shift acquisition mode (3 × 3 holes; 7 exposures per hole) at a nominal magnification of ×165,000 with a pixel size of 0.5113 Å per pixel. The Gatan K3 detector was used in correlated-double sampling mode and energy filter with the energy slit set to 10 eV. Movies were collected for 2 sec at a flux of 25 electrons per Å^2^ per second, giving a total exposure of 50 electrons per Å^2^. The defocus values ranged from −0.5 to −3.0 μm. Forty frames of each movie were saved.

#### Cryo-EM image processing

All data processing (Supplementary Figs. 42, 43, and Supplementary Table 13) was performed using the Relion 4.0 software package^83^. Motion correction was performed using the RELION implementation of MotionCor2^84^. Movies were aligned using 7 × 5 patches with dose weighting. A contrast transfer function (CTF) was estimated using CTFFIND4.1^85^ from summed power spectra^86^ for every 4 electrons per Å^2^. 50 micrographs were randomly selected, and a representative set of particles was picked manually. These particle coordinates were used for training a Topaz picking model and Topaz model was used for subsequent particle picking^87^. After initial binning, particles underwent two rounds of 2D-classification. In each round, the particles were sorted into 200 classes with an E-step of 8 Å and a mask diameter of 220 Å. Only classes with well-defined structural features were retained and subjected to 3D-classification using a reference from the crystal structure PDB ID 4ZXB^64^. The first 3D classification sorted particles into ten classes with the regularization parameter set to T = 4. Selected classes were aligned into a global 3D auto-refinement. A subsequent 3D classification, using the result of the previous 3D auto-refinement as input along with mask focused on the area around L2 and FnIII-1 domains and density for the HIR-6 aptamers, was performed using local searches from 3.7 to 1.8 degrees with the regularization parameter T = 4. Particles with poorly defined structural features were removed, and the remaining particles were re-extracted to two times binned pixel size. Particles were 3D refined with focused mask on the area around L2 and FnIII-1 domains and density for the HIR-6 aptamers, and using C2 relaxed symmetry. The 3D refinement was improved by correction for microscope aberrations within Relion 4.0. Final focused 3D classification was performed without angular alignment and regularization parameter set to T = 20. For the final 3D refinement, the particles were Bayesian-polished and further corrected for microscope aberrations. The final cryo-EM density maps were generated by the post-processing feature in RELION and sharpened or blurred into MTZ format using CCP-EM^88^. The final set was B = −200, −100, −50, 0, 50, 100, 200 Å^2^ sharpened/blurred MTZ maps. The resolution of the cryo-EM density maps (Supplementary Table 13, Supplementary Figs. 42-43) was estimated with the gold standard Fourier shell correlation (FSC) cut-off value of 0.143. Reference-based local amplitude scaling was performed by LocScale^89^ around the modeled domains of FnIII-1, L2’ and HIR-6. The angular orientation distribution of the 3D reconstruction was calculated by cryoEF v1.1.06^90^. Local resolution was calculated within Relion 4.0.

#### Cryo-EM model building and refinement

The model is limited to the HIR domain FnIII-1 and L2’, where the HIR-6 aptamer interacts with the protein moiety. Specifically, modeled residues span FnIII-1/469-519, FnIII-1/527-573, FnIII-1/578-592 and L2’/308-468. The model for the HIR-6 aptamer is limited to residues 20 to 38, the cryo-EM density map for the rest of the aptamer is not interpretable, only showing low resolution secondary structure features for the T19-A39 base pair. The X-ray structure of the HIR ectodomain (PDB ID 4zbx)^64^ was used as a starting model. One protomer of the HIR ectodomain was docked into the cryo-EM map by Molrep^91^ and only domain L2’ and FnIII-1 were retained for further modeling. The model for HIR-6 aptamer was modeled manually in Coot 0.8.9.1^92^ together with adjustments to the HIR ectodomain protein moiety. Refinement in Coot was done against a blurred MTZ map (blurring B = 50 Å^2^) generated in CCP-EM^88^. The aptamer register of residues 20 to 38 was verified by cryo-EM density matching. Ligand geometry restraints for modified DNA bases were generated using Acedrg^93^ within the CCP4i2 package^94^. Atom naming of the modified nucleotides was matched with the respective unmodified nucleotides from the monomer library, and the “NON-POLYMER” identifier was manually changed to “DNA” to ensure that the monomer units seamlessly link into the DNA chain. Model self-restraints were used, as well as base pairing and parallelity restraints for DNA, which were automatically generated by the program libG^95^ running under Refmac 5.8.0405^96^ within the CCP4 Interface 8.0.010^97^ and curated manually. The final model was refined in real space^98^ against a post-processed MRC map in Phenix 1.21-5207^99^, using self-restraints with the strict rotamer matching option enabled, as well as secondary structure restraints, including base pairing and parallelity restraints for DNA. The restraints were generated automatically in Phenix 1.21-5207^99^. The final refinement round in Phenix included one cycle of ADP refinement only. The refined models were validated using MolProbity^100^, EMRinger within Phenix^101^ and the wwPDB database^102^ validation server. For Supplementary Fig. 43b all individual domains of HIR were rigid body fitted to low resolution cryo-EM contours by Jiggle-Fit tool in Coot. Structure analysis was performed using ChimeraX^103^, Coot^104^, and PISA^105^ server. Non-covalent interactions were identified in ChimeraX with relaxed geometry criteria enabled. ChimeraX and Pymol^106^ were used for structure visualization.

#### NMR analysis

NMR spectra were measured on a Bruker Avance III HD 400 and 500 MHz spectrometers (^1^H at 401 or 500.0 MHz, ^13^C at 101 or 126 MHz and ^31^P at 202 MHz) in DMSO-d6 (referenced to δ(CHD_2_SO_2_CD_3_) = 2.50 ppm and δ(CD_3_SO_2_CD_3_) = 39.7 ppm) or D_2_O (referenced to either to tertbutanol or dioxane) at 25 °C. Chemical shifts are given in ppm (δ-scale) and coupling constants (J) in Hz. Complete assignment of all NMR signals was achieved by using a combination of H,H-COSY, H,C-HSQC and H,C-HMBC 2D NMR experiments.

#### LC-MS analysis

LC-MS was performed on an Agilent 1920 Infinity II BIO system with an MSD XT mass spectrometer equipped with an ESI ion source, using a Phenomenex Biozen 1.7 μm Oligo 50×2.1 mm column and applying a 30-min gradient from 95% A (300 mM HFIP + 15 mM TEA in H_2_O) to 50% B (300 mM HFIP + 15 mM TEA in MeOH). Samples were HPLC-purified, reconstituted in water, and injected at ~5 µM concentration. Data were acquired under these MS settings: 3000 V capillary voltage, 12 L/min drying gas flow, 350 °C drying gas temperature and 35 psi nebulizer pressure. Acquired spectra were exported from the Agilent OpenLab Chemstation program and deconvoluted using the UniDec^107^ program with full m/z negative range, no background subtraction, no suppression of artifacts, and a Gaussian peak shape function.

#### Statistics & Reproducibility

No statistical method was used to predetermine sample size. The experiments were not randomized. Investigators were not blinded to allocation during the experiments and outcome assessment.

### Reporting summary

Further information on research design is available in the Nature Portfolio Reporting Summary linked to this article.

## Supplementary information

Supplementary Information

Reporting Summary

Transparent Peer Review file

## Source data

Source Data

## Data Availability

The source data supporting the findings of this study are available in the Supplementary Information file, Source Data file, as well as in public repositories: Raw NGS data were deposited in NCBI SRA repository^108^, under accession code PRJNA1467126 (https://www.ncbi.nlm.nih.gov/bioproject/1467126), model coordinates and maps for the HIR-HIR-6 complex in PDB database^109^ under accession code 9SA8 and MEDB database^110^ under accession code EMD-54689. Additional raw data, including the cryo-EM PDB model and map, raw LC-MS data and raw NMR data are also available via the BioStudies repository (10.6019/S-BSST2268)^111^. Source data are provided with this paper.

## Code availability

Scripts used for k-mer PCA plots and covariation analysis are provided in BioStudies repository (10.6019/S-BSST2268)^111^.
