# Supplementary material for: Expedient single-round selection of hyper-modified aptamer targeting insulin receptor from over-represented dually nucleobase-modified DNA libraries
Source: Nat Commun. 2026 May 27;17:6895. doi: 10.1038/s41467-026-73676-y (PMC13389011; doi:10.1038/s41467-026-73676-y)
Supplement: Supplementary file 1 — Supplementary Information [file 41467_2026_73676_MOESM1_ESM.pdf]

Supporting Information

Expedient Single-round Selection of Hyper-modified Aptamer Targeting Insulin Receptor from Over-represented Dually Nucleobase-modified DNA Libraries

Pablo Alberto Franco-Urquijo, Marek Ondruš, Jaroslav Kurfürst, Jana Škerlová, Irena Selicharová, Lucie Mužíková Čechová, Hana Šváchová, Alena Semerádtová, Anatolij Filimoněnko, Adéla Fejfarová, Jiří Homola, Tomáš Kouba and Michal Hocek

Table of contents

1. Oligonucleotide / Aptamer sequences 3
1.1 Oligonucleotides used in this study 3
1.2 Libraries and oligonucleotides synthesized in this study 6
2. Chemical synthesis and compound characterizations 12
2.1 Sonogashira cross-coupling reaction 12
2.2 Catalytic hydrogenation 13
2.3 Triphosphorylation 14
3. Enzymatic synthesis of DNA libraries 16
4. Insulin receptors as targets 17
4.1 Amino acid sequence alignments of HIR with CIR and DIR 17
5. Aptamer selections 21
5.1 Single-round aptamer selection....................21
5.2 Selection for covariation analysis 21
6. Next-Generation Sequencing (NGS) and data analysis 21
6.1 Sample preparation and NGS 21
6.2 Distributions of sequence abundances....................24
6.3 Cluster analysis 28
6.4 Sequence alignment of most active clusters for L2 and L2' selections 32
6.5 Covariation analysis....................33
7. Enzymatic synthesis of modified oligonucleotides 33
7.1 Generation of single-stranded DNA using magnetic beads 34
8. Binding affinity and specificity assays 40
8.1 Fluorescent Ni-plate binding assay (FNBA)....................40
8.2 Microscale thermophoresis (MST) 41
9. Cell assays 42
9.1 Receptor-binding studies 42
9.2 Receptor phosphorylation and antagonism assay 43
10. Cryo-electron microscopy (cryo-EM) of HIR-HIR-6 complex 45
10.1 HIR-6 interactions 50
11. Copies of NMR spectra of prepared compounds 52
12. MS characterization of synthesized modified ONs 56
13. Supplementary References 77

1. Oligonucleotide / Aptamer sequences

1.1 Oligonucleotides used in this study

Supplementary Table 1. Sequences of natural templates, primers, libraries and candidates used in this study.

ON title	Sequence (5'→3')	nt
L1_temp[b]	AGTCACACAGAAGTGACGTC-N15-ACGAAGGCTGTATAGATGCG	55
L2_temp[b]	GACATCATGAGAGACATCGC-N15-GAGATACAGTGAGCGTCATG	55
L2_N13_temp[b]	GACATCATGAGAGACATCGC-N13-GAGATACAGTGAGCGTCATG	53
L2_N17_temp[b]	GACATCATGAGAGACATCGC-N17-GAGATACAGTGAGCGTCATG	57
L2_N19_temp[b]	GACATCATGAGAGACATCGC-N19-GAGATACAGTGAGCGTCATG	59
L2_N22_temp[b]	GACATCATGAGAGACATCGC-N22-GAGATACAGTGAGCGTCATG	62
L3_temp[b,d]	GTAGACACATCTGACGAGCAGACATCATGAGAGACATCGCTGTTACGTTAAACGCGAGATACAGTGAGCGTCATGGAGACGCTGATGAGATTAGC	95
L4_temp[b]	GACCGTTTACTTGCTGCTAG-N15-ACATGGACCGGTAGAAGTTG	55
Rev1[a]	CGCATCTATACAGCCTTCGT	20
Rev2[a]	CATGACGCTCACTGTATCTC	20
Rev3[a,c]	GCTAATCTCATCAGCGTCTC	20
Fw3[b]	GTAGACACATCTGACGAGCA	20
Rev4[a]	CAACTTCTACCGGTCCATGT	20
Rev1_adapter	TCGTCGGCAGCGTCAGATGTGTATAAGAGACAGCGCATCTATACAGCCTTCGT	53
Fw1_adapter	GTCTCGTGGGCTCGGAGATGTGTATAAGAGACAGAGTCACACAGAAGTGACGTC	54
Rev2_adapter	TCGTCGGCAGCGTCAGATGTGTATAAGAGACAGCATGACGCTCACTGTATCTC	53
Fw2_adapter	GTCTCGTGGGCTCGGAGATGTGTATAAGAGACAGGACATCATGAGAGACATCGC	54
Rev3_adapter	TCGTCGGCAGCGTCAGATGTGTATAAGAGACAGGCTAATCTCATCAGCGTCTC	53
Fw3_adapter	GTCTCGTGGGCTCGGAGATGTGTATAAGAGACAGGTAGACACATCTGACGAGCA	54
Rev4_adapter	TCGTCGGCAGCGTCAGATGTGTATAAGAGACAGCAACTTCTACCGGTCCATGT	53
Fw4_adapter	GTCTCGTGGGCTCGGAGATGTGTATAAGAGACAGGACCGTTTACTTGCTGCTAG	54
L1_c1	CGCATCTATACAGCCTTCGTATGACTACGTACTAGGACGTCACTTCTGTGTGACT	55
L1_c2	CGCATCTATACAGCCTTCGTGTCATACCATCACCGGACGTCACTTCTGTGTGACT	55
L1_c3	CGCATCTATACAGCCTTCGTCTGCAAGCGGATACAGACGTCACTTCTGTGTGACT	55
L1_c4	CGCATCTATACAGCCTTCGTAGGGGGGGTCAGAGAGACGTCACTTCTGTGTGACT	55

L1_c5	CGCATCTATACAGCCTTCGTCAACTTCCGTCCTATGACGTCACTTC TGTGTGACT	55
L2_c1_temp[b]	AGTCACACAGAAGTGACGTCTTCGAGATAGGTCGAACGAAGGCTG TATAGATGCG	55
L2_c2_temp[b]	AGTCACACAGAAGTGACGTCCCATTAGATGTAAGGACGAAGGCTG TATAGATGCG	55
L2_c3_temp[b]	AGTCACACAGAAGTGACGTCTGCAAACGAAGTCTGACGAAGGCTG TATAGATGCG	55
L2_c4_temp[b]	AGTCACACAGAAGTGACGTCGCCCGTAGATGTCTAACGAAGGCTG TATAGATGCG	55
L2_c5_temp[b]	AGTCACACAGAAGTGACGTCTTACGTTAAAGCGGCACGAAGGCTG TATAGATGCG	55
L3_c1_temp[b]	AGTCACACAGAAGTGACGTCGCGAAGTGTAGATGCACGAAGGCT GTATAGATGCG	55
L3_c2_temp[b]	AGTCACACAGAAGTGACGTCTCGAATATCGGGGGCACGAAGGCT GTATAGATGCG	55
L3_c3_temp[b]	AGTCACACAGAAGTGACGTCCCTAGAATGGACCCTACGAAGGCTG TATAGATGCG	55
L3_c4_temp[b]	AGTCACACAGAAGTGACGTCGGGCCATTCAGCTCGACGAAGGCT GTATAGATGCG	55
L3_c5_temp[b]	AGTCACACAGAAGTGACGTCGAGGTTAACATGGGCACGAAGGCT GTATAGATGCG	55
L2'_c1_temp[b]	GACATCATGAGAGACATCGCGTGTTACGTTAAACCGAGATACAGT GAGCGTCATG	55
L2'_c2_temp[b]	GACATCATGAGAGACATCGCTCTCTTCACACACAAGAGATACAGT GAG CGTCATG	55
L2'_c3_temp[b]	GACATCATGAGAGACATCGCGTCTTACGTTAAAGCGAGATACAGT GAGCGTCATG	55
L2'_c4_temp[b]	GACATCATGAGAGACATCGCCACCTTGAGTCTAACGAGATACAGT GAGCGTCATG	55
L2'_c5_temp[b]	GACATCATGAGAGACATCGCTCGTTTGAGGCTAACGAGATACAGT GAGCGTCATG	55
L2'_c6_temp[b]	GACATCATGAGAGACATCGCTGTTACGTTAAACGCGAGATACAGT GAGCGTCATG	55
L2'_c7_temp[b]	GACATCATGAGAGACATCGCTACCTTGAGTCTAACGAGATACAGT GAGCGTCATG	55
L2'_c8_temp[b]	GACATCATGAGAGACATCGCTCTTACGTTAAAGGCGAGATACAGT GAGCGTCATG	55
L2'_c9_temp[b]	GACATCATGAGAGACATCGCTACCTTGAGGCTTACGAGATACAGT GAGCGTCATG	55
L2'_c10_temp[b]	GACATCATGAGAGACATCGCTGTTACGTTAAACCCGAGATACAGT GAGCGTCATG	55
L2'_c11_temp[b]	GACATCATGAGAGACATCGCTACCTTGAGGCTAACGAGATACAGT GAGCGTCATG	55
L2'_c12_temp[b]	GACATCATGAGAGACATCGCACCCTTGAGTCTAACGAGATACAGT GAGCGTCATG	55
L2'_c13_temp[b]	GACATCATGAGAGACATCGCCTGTTACGTTAAACGGAGATACAGT GAGCGTCATG	55
L2'_c14_temp[b]	GACATCATGAGAGACATCGCACGTTTGAGGCTAACGAGATACAGT GAGCGTCATG	55

L2'_c15_temp[b]	GACATCATGAGAGACATCGCGCTCGATCTACAAAAGAGATACAGT GAGCGTCATG	55
L2'_c16_temp[b]	GACATCATGAGAGACATCGCTCGGGATCTACAAAAGAGATACAGT GAGCGTCATG	55
L2'_c17_temp[b]	GACATCATGAGAGACATCGCGCATATCATGATAACGAGATACAGT GAGCGTCATG	55
L2'_c18_temp[b]	GACATCATGAGAGACATCGCTCCGTTGAGTCTAACGAGATACAGT GAGCGTCATG	55
L2'_c19_temp[b]	GACATCATGAGAGACATCGCTTGTAACAATCGCAAGAGATACAGT GAGCGTCATG	55
L2'_c20_temp[b]	GACATCATGAGAGACATCGCTTGTAACAATCGGCAGAGATACAGT GAGCGTCATG	55
L2'_c21_temp[b]	GACATCATGAGAGACATCGCCCATATCATGATAACGAGATACAGT GAG CGTCATG	55
L2'_c22_temp[b]	GACATCATGAGAGACATCGCTTTCATAAGCGACATGAGATACAGT GAG CGTCATG	55
L2'_c23_temp[b]	GACATCATGAGAGACATCGCAACCTTGAGGCTTACGAGATACAGT GAGCGTCATG	55
L2'_c24_temp[b]	GACATCATGAGAGACATCGCTCGAGTATAATGCAAGAGATACAGT GAGCGTCATG	55
L2'_c25_temp[b]	GACATCATGAGAGACATCGCCTCTTACGTTAAAGGGAGATACAGT GAGCGTCATG	55
L2'_c26_temp[b]	GACATCATGAGAGACATCGCACCTTTGAGTCTAACGAGATACAGT GAGCGTCATG	55
L2'_c27_temp[b]	GACATCATGAGAGACATCGCGCTGGATCTACAAAAGAGATACAGT GAGCGTCATG	55
L2'_c28_temp[b]	GACATCATGAGAGACATCGCCACCTTGAGGCTTACGAGATACAGT GAGCGTCATG	55
L2'_c29_temp[b]	GACATCATGAGAGACATCGCGTTCGATCTACAAAAGAGATACAGT GAGCGTCATG	55
L2'_N13_c1_temp[b]	GACATCATGAGAGACATCGCACTTGAGGCTAACGAGATACAGTGA GCGTCATG	53
L2'_N13_c2_temp[b]	GACATCATGAGAGACATCGCCCGATATGATGATGAGATACAGTGA GCGTCATG	53
L2'_N13_c3_temp[b]	GACATCATGAGAGACATCGCGAAAAGAAAGCATGAGATACAGTGA GCGTCATG	53
L2'_N13_c4_temp[b]	GACATCATGAGAGACATCGCTGTTACGTTAAACGAGATACAGTGA GCGTCATG	53
L2'_N13_c5_temp[b]	GACATCATGAGAGACATCGCTGGATCTACAAAAGAGATACAGTGA GCGTCATG	53
L2'_N17_c1_temp[b]	GACATCATGAGAGACATCGCTTGATAGGTCAAGGATGGAGATACA GTGAGCGTCATG	57
L2'_N17_c2_temp[b]	GACATCATGAGAGACATCGCGTGTTACGTTAAACCGTGAGATACA GTGAGCGTCATG	57
L2'_N17_c3_temp[b]	GACATCATGAGAGACATCGCGTGTGATAGGTCACCATGAGATACA GTGAGCGTCATG	57
L2'_N17_c4_temp[b]	GACATCATGAGAGACATCGCCCGTCTTACGTTAAAGCGAGATACA GTGAGCGTCATG	57
L2'_N17_c5_temp[b]	GACATCATGAGAGACATCGCCCAAGGGATAATCGGCAGAGATACA GTGAGCGTCATG	57

L4_c1_temp[b]	GACCGTTTACTTGCTGCTAGCTGTCGGCATCACAAACATGGACCGGTAGAAGTTG	55
L4_c2_temp[b]	GACCGTTTACTTGCTGCTAGATATCGTATCATGTGACATGGACCGGTAGAAGTTG	55
L4_c3_temp[b]	GACCGTTTACTTGCTGCTAGTGATAGGTCACAATGACATGGACCGGTAGAAGTTG	55
L4_c4_temp[b]	GACCGTTTACTTGCTGCTAGATAGGTCAATACAGCACATGGACCGGTAGAAGTTG	55
L4_c5_temp[b]	GACCGTTTACTTGCTGCTAGATCTCTAAGCGCATCACATGGACCGGTAGAAGTTG	55
HIR-6_N[a]	CATGACGCTCACTGTATCTCGCGTTTAACGTAACAGCGATGTCTCTCATGATGTC	55
HIR-8_N[a]	CATGACGCTCACTGTATCTCGCCTTTAACGTAAGAGCGATGTCTCTCATGATGTC	55
DIR-5_N[a]	CAACTTCTACCGGTCCATGTTGTGATGCCTGACAGCTAGCAGCAAGTAAACGGTC	55
SC_temp[b]	CTACGACTCAATGGTAGTATTCAACGCGTGCGAAAAGTTAAAGGCCAGGTGCAGT	55
SC2_temp[b]	TTAGACAGTTGTCGTTCGTTCTCCGCCATAACGCATATCAGAGCGTAAGGGCAGT	55
Rev2_SC[a]	ACTGCACCTGGCCTTTAACT	20
Rev4_SC[a]	ACTGCCCTTACGCTCTGATA	20
T1_temp[b]	5CATGAGAGACATCGCTGTTACGTTAAACGCGAGATACAGTGAGCGTCATG	50
T2_temp[b]	GAGACATCGCTGTTACGTTAAACGCGAGATACAGTGAGCGTCATG	45
T3_temp[b]	ATCGCTGTTACGTTAAACGCGAGATACAGTGAGCGTCATG	40
Rev2A[a]	CGCTCACTGTATCTC	15
Rev2B[a]	ACTGTATCTC	10
Rev2C[a]	TATCTCGCG	9
Rev2D[a]	CATGACGCTCACTGTATCTCGCGTTTAA	28

[a] 5'-Cy5; [b] 5'-Biotin; [c] 5'-Phosphate; [d] mutagenized (21%) part underlined.

1.2 Libraries and oligonucleotides synthesized in this study

Supplementary Table 2. Sequences of modified libraries, candidates and aptamers synthesized in this study.

ON title	Sequence (5'→3')	nt	dN^{R}TPs	Template	Primer
L1[a]	CGCATCTATACAGCCTTCGT-N15-GACGTCACTTCTGTGTGACT	55	-,-	L1_temp[b]	Rev1[a]

L2[a]	CGCATCTATACAGCCTTCGT-N15-GACGUCACUUCUGUGUGACU	55	1,2	L1_temp[b]	Rev1[a]
L3[a]	CGCATCTATACAGCCTTCGT-N15-GACGUCACUUCUGUGUGACU	55	3,4	L1_temp[b]	Rev1[a]
L2'[a]	CATGACGCTCACTGTATCTC-N15-GCGAUGUCUCUCAUGAUGUC	55	1,2	L2_temp[b]	Rev2[a]
L2'_N13[a]	CATGACGCTCACTGTATCTC-N13-GCGAUGUCUCUCAUGAUGUC	53	1,2	L2_N13_temp[b]	Rev2[a]
L2'_N17[a]	CATGACGCTCACTGTATCTC-N17-GCGAUGUCUCUCAUGAUGUC	57	1,2	L2_N17_temp[b]	Rev2[a]
L2'_N19[a]	CATGACGCTCACTGTATCTC-N19-GCGAUGUCUCUCAUGAUGUC	59	1,2	L2_N19_temp[b]	Rev2[a]
L2'_N22[a]	CATGACGCTCACTGTATCTC-N22-GCGAUGUCUCUCAUGAUGUC	62	1,2	L2_N22_temp[b]	Rev2[a]
L4[a,c]	GCTAATCTCATCAGCGTCTCCAUGACGCUCACUGUAUCUCGCGUUUAACGUAACAGCGAUGUCUCUCAUGAUGUCUGCUCGUCAGAUGUGUCUAC	95	1,2	L3_temp[b]	Rev3[a]
L5 [a]	CAACTTCTACCGGTCCATGT-N15-CUAGCAGCAAGUAAACGGUC	55	1,2	L4_temp[b]	Rev4[a]
L2_c1[a]	CGCATCTATACAGCCTTCGTUCGACCUAUCUCGAAGACGUCACUUCUGUGUGACU	55	1,2	L2_c1_temp[b]	Rev1[a]
L2_c2[a]	CGCATCTATACAGCCTTCGTCCUUACAUCUAAUGGGACGUCACUUCUGUGUGACU	55	1,2	L2_c2_temp[b]	Rev1[a]
L2_c3[a]	CGCATCTATACAGCCTTCGTCAGACUUCGUUUGCAGACGUCACUUCUGUGUGACU	55	1,2	L2_c3_temp[b]	Rev1[a]
L2_c4[a]	CGCATCTATACAGCCTTCGTUAGACAUCUACGGGCGACGUCACUUCUGUGUGACU	55	1,2	L2_c4_temp[b]	Rev1[a]
L2_c5[a]	CGCATCTATACAGCCTTCGTGCCGCUUUAACGUAAGACGUCACUUCUGUGUGACU	55	1,2	L2_c5_temp[b]	Rev1[a]
L3_c1[a]	CGCATCTATACAGCCTTCGTGCAUCUACACUUCGCGACGUCACUUCUGUGUGACU	55	3,4	L3_c1_temp[b]	Rev1[a]
L3_c2[a]	CGCATCTATACAGCCTTCGTGCCCCCGAUAUUCGAGACGUCACUUCUGUGUGACU	55	3,4	L3_c2_temp[b]	Rev1[a]

L3_c3[a]	CGCATCTATACAGCCTTCGTA GGGUCCAUUCUAGGGACGU CACUUCUGUGUGACU	55	3,4	L3_c3_temp[b]	Rev1[a]
L3_c4[a]	CGCATCTATACAGCCTTCGTC GAGCUGAAUGGCCCGACGU CACUUCUGUGUGACU	55	3,4	L3_c4_temp[b]	Rev1[a]
L3_c5[a]	CGCATCTATACAGCCTTCGTG CCCAUGUUAACCUCGACGUC ACUUCUGUGUGACU	55	3,4	L3_c5_temp[b]	Rev1[a]
HIR-1[a]	CATGACGCTCACTGTATCTCG GUUUAACGUAACACGCGAUG UCUCUCAUGAUGUC	55	1,2	L2'_c1_temp[b]	Rev2[a]
HIR-2[a]	CATGACGCTCACTGTATCTCU UGUGUGUGAAGAGAGCGAU GUCUCUCAUGAUGUC	55	1,2	L2'_c2_temp[b]	Rev2[a]
HIR-3[a]	CATGACGCTCACTGTATCTCG CUUUAACGUAAGACGCGAUG UCUCUCAUGAUGUC	55	1,2	L2'_c3_temp[b]	Rev2[a]
HIR-4[a]	CATGACGCTCACTGTATCTCG UUAGACUCAAGGUGGCGAUG UCUCUCAUGAUGUC	55	1,2	L2'_c4_temp[b]	Rev2[a]
HIR-5[a]	CATGACGCTCACTGTATCTCG UUAGCCUCAAACGAGCGAUG UCUCUCAUGAUGUC	55	1,2	L2'_c5_temp[b]	Rev2[a]
HIR-6[a,b]	CATGACGCTCACTGTATCTCG CGUUUAACGUAACAGCGAUG UCUCUCAUGAUGUC	55	1,2	L2'_c6_temp[b]	Rev2[a]
HIR-7[a]	CATGACGCTCACTGTATCTCG UUAGACUCAAGGUAGCGAUG UCUCUCAUGAUGUC	55	1,2	L2'_c7_temp[b]	Rev2[a]
HIR-8[a]	CATGACGCTCACTGTATCTCG CCUUUAACGUAAGAGCGAUG UCUCUCAUGAUGUC	55	1,2	L2'_c8_temp[b]	Rev2[a]
HIR-9[a]	CATGACGCTCACTGTATCTCG UAAGCCUCAAGGUAGCGAUG UCUCUCAUGAUGUC	55	1,2	L2'_c9_temp[b]	Rev2[a]
HIR-10[a]	CATGACGCTCACTGTATCTCG GGUUUAACGUAACAGCGAUG UCUCUCAUGAUGUC	55	1,2	L2'_c10_temp[b]	Rev2[a]
HIR-11[a]	CATGACGCTCACTGTATCTCG UUAGCCUCAAGGUAGCGAUG UCUCUCAUGAUGUC	55	1,2	L2'_c11_temp[b]	Rev2[a]
HIR-12[a]	CATGACGCTCACTGTATCTCG UUAGACUCAAGGGUGCGAUG UCUCUCAUGAUGUC	55	1,2	L2'_c12_temp[b]	Rev2[a]
HIR-13[a]	CATGACGCTCACTGTATCTCC GUUUAACGUAACAGGCGAUG UCUCUCAUGAUGUC	55	1,2	L2'_c13_temp[b]	Rev2[a]

HIR-14[a]	CATGACGCTCACTGTATCTCGUUAGCCUCAAACGUGCGAUGUCUCUCAUGAUGUC	55	1,2	L2'_c14_temp[b]	Rev2[a]
HIR-15[a]	CATGACGCTCACTGTATCTCUUUUGUAGAUCGAGCGCGAUGUCUCUCAUGAUGUC	55	1,2	L2'_c15_temp[b]	Rev2[a]
HIR-16[a]	CATGACGCTCACTGTATCTCUUUUGUAGAUCCCGAGCGAUGUCUCUCAUGAUGUC	55	1,2	L2'_c16_temp[b]	Rev2[a]
HIR-17[a]	CATGACGCTCACTGTATCTCGUUAUCAUGAUAUGCGCGAUGUCUCUCAUGAUGUC	55	1,2	L2'_c17_temp[b]	Rev2[a]
HIR-18[a]	CATGACGCTCACTGTATCTCGUUAGACUCAACGGAGCGAUGUCUCUCAUGAUGUC	55	1,2	L2'_c18_temp[b]	Rev2[a]
HIR-19[a]	CATGACGCTCACTGTATCTCUUGCGAUUGUUACAAGCGAUGUCUCUCAUGAUGUC	55	1,2	L2'_c19_temp[b]	Rev2[a]
HIR-20[a]	CATGACGCTCACTGTATCTCUGCCGAUUGUUACAAGCGAUGUCUCUCAUGAUGUC	55	1,2	L2'_c20_temp[b]	Rev2[a]
HIR-21[a]	CATGACGCTCACTGTATCTCGUUAUCAUGAUAUGGGCGAUGUCUCUCAUGAUGUC	55	1,2	L2'_c21_temp[b]	Rev2[a]
HIR-22[a]	CATGACGCTCACTGTATCTCAUGUCGCUUAUGAAAGCGAUGUCUCUCAUGAUGUC	55	1,2	L2'_c22_temp[b]	Rev2[a]
HIR-23[a]	CATGACGCTCACTGTATCTCGUAAGCCUCAAGGUUGCGAUGUCUCUCAUGAUGUC	55	1,2	L2'_c23_temp[b]	Rev2[a]
HIR-24[a]	CATGACGCTCACTGTATCTCTGCAUUAUACUCGAGCGAUGUCUCUCAUGAUGUC	55	1,2	L2'_c24_temp[b]	Rev2[a]
HIR-25[a]	CATGACGCTCACTGTATCTCCCUUUAACGUAAGAGGCGAUGUCUCUCAUGAUGUC	55	1,2	L2'_c25_temp[b]	Rev2[a]
HIR-26[a]	CATGACGCTCACTGTATCTCGUUAGACUCAAAGGUGCGAUGUCUCUCAUGAUGUC	55	1,2	L2'_c26_temp[b]	Rev2[a]
HIR-27[a]	CATGACGCTCACTGTATCTCUUUUGUAGAUCCAGCGCGAUGUCUCUCAUGAUGUC	55	1,2	L2'_c27_temp[b]	Rev2[a]
HIR-28[a]	CATGACGCTCACTGTATCTCGUAAGCCUCAAGGUGGCGAUGUCUCUCAUGAUGUC	55	1,2	L2'_c28_temp[b]	Rev2[a]
HIR-29[a]	CATGACGCTCACTGTATCTCUUUUGUAGAUCGAACGCGAUGUCUCUCAUGAUGUC	55	1,2	L2'_c29_temp[b]	Rev2[a]

L2'_N13_c1[a]	CATGACGCTCACTGTATCTCGUUAGCCUCAAGUGCGAUGUCUCUCAUGAUGUC	53	1,2	L2'_N13_c1_temp[b]	Rev2[a]
L2'_N13_c2[a]	CATGACGCTCACTGTATCTCAUCAUCAUAUCGGGCGAUGUCUCUCAUGAUGUC	53	1,2	L2'_N13_c2_temp[b]	Rev2[a]
L2'_N13_c3[a]	CATGACGCTCACTGTATCTCAUGCUUUCUUUUCGCGAUGUCUCUCAUGAUGUC	53	1,2	L2'_N13_c3_temp[b]	Rev2[a]
L2'_N13_c4[a]	CATGACGCTCACTGTATCTCGUUUAACGUAACAGCGAUGUCUCUCAUGAUGUC	53	1,2	L2'_N13_c4_temp[b]	Rev2[a]
L2'_N13_c5[a]	CATGACGCTCACTGTATCTCUUUUGUAGAUCCAGCGAUGUCUCUCAUGAUGUC	53	1,2	L2'_N13_c5_temp[b]	Rev2[a]
L2'_N17_c1[a]	CATGACGCTCACTGTATCTCCAUCCUUGACCUAUCAAGCGAUGUCUCUCAUGAUGUC	57	1,2	L2'_N17_c1_temp[b]	Rev2[a]
L2'_N17_c2[a]	CATGACGCTCACTGTATCTCACGGUUUAACGUAACACGCGAUGUCUCUCAUGAUGUC	57	1,2	L2'_N17_c2_temp[b]	Rev2[a]
L2'_N17_c3[a]	CATGACGCTCACTGTATCTCAUGGUGACCUAUCACACGCGAUGUCUCUCAUGAUGUC	57	1,2	L2'_N17_c3_temp[b]	Rev2[a]
L2'_N17_c4[a]	CATGACGCTCACTGTATCTCGCUUUAACGUAAGACGGGCGAUGUCUCUCAUGAUGUC	57	1,2	L2'_N17_c4_temp[b]	Rev2[a]
L2'_N17_c5[a]	CATGACGCTCACTGTATCTCUGCCGAUUAUCCCUUGGGCGAUGUCUCUCAUGAUGUC	57	1,2	L2'_N17_c5_temp[b]	Rev2[a]
DIR-1[a]	CAACTTCTACCGGTCCATGTUUGUGAUGCCGACAGCUAGCAGCAAGUAAACGGUC	55	1,2	L4_c1_temp[b]	Rev4[a]
DIR-2[a]	CAACTTCTACCGGTCCATGTCACAUGAUACGAUAUCUAGCAGCAAGUAAACGGUC	55	1,2	L4_c2_temp[b]	Rev4[a]
DIR-3[a]	CAACTTCTACCGGTCCATGTCAUUGUGACCUAUCACUAGCAGCAAGUAAACGGUC	55	1,2	L4_c3_temp[b]	Rev4[a]
DIR-4[a]	CAACTTCTACCGGTCCATGTGCUGUAUUGACCUAUCUAGCAGCAAGUAAACGGUC	55	1,2	L4_c4_temp[b]	Rev4[a]
DIR-5[a]	CAACTTCTACCGGTCCATGTGAUGCGCUUAGAGAUCUAGCAGCAAGUAAACGGUC	55	1,2	L4_c5_temp[b]	Rev4[a]
HIR_SC[a]	ACTGCACCTGGCCTTTAACTUUUCGCACGCGUUGAAUACUACCAUUGAGUCGUAG	55	1,2	SC_temp[b]	Rev2_SC[a]

HIR-6_V1[a]	CATGACGCTCACTGTATCTCGCGUUUAACGUAACAGCGAUGUCUCUCAUGAUGUC	55	3,4	L2'_c6_temp[b]	Rev2[a]
HIR-6_V2[a]	CATGACGCTCACTGTATCTCGCGUUUAACGUAACAGCGAUGUCUCUCAUGAUGUC	55	3,2	L2'_c6_temp[b]	Rev2[a]
HIR-6_V3[a]	CATGACGCTCACTGTATCTCGCGUUUAACGUAACAGCGAUGUCUCUCAUGAUGUC	55	1,4	L2'_c6_temp[b]	Rev2[a]
HIR-6_V4[a]	CATGACGCTCACTGTATCTCGCGUUUAACGUAACAGCGAUGUCUCUCAUGAUGUC	55	5,2	L2'_c6_temp[b]	Rev2[a]
DIR_SC[a]	ACTGCCCTTACGCTCTGATAUGCGUUAUGGCGGAGAACGAACGACAACUGUCUAA	55	1,2	SC2_temp[b]	Rev2_SC[a]
HIR-6_T1[a]	CATGACGCTCACTGTATCTCGCGUUUAACGUAACAGCGAUGUCUCUCAUG	50	1,2	T1_temp[b]	Rev2[a]
HIR-6_T2[a]	CATGACGCTCACTGTATCTCGCGUUUAACGUAACAGCGAUGUCUC	45	1,2	T2_temp[b]	Rev2[a]
HIR-6_T3[a]	CATGACGCTCACTGTATCTCGCGUUUAACGUAACAGCGAU	40	1,2	T3_temp[b]	Rev2[a]
HIR-6_T4[a]	CGCTCACTGTATCTCGCGUUUAACGUAACAGCGAUGUCUCUCAUGAUGUC	50	1,2	L2'_c6_temp[b]	Rev2A[a]
HIR-6_T5[a]	ACTGTATCTCGCGUUUAACGUAACAGCGAUGUCUCUCAUGAUGUC	45	1,2	L2'_c6_temp[b]	Rev2B[a]
HIR-6_T6[a]	TATCTCGCGUUUAACGUAACAGCGAUGUCUCUCAUGAUGUC	41	1,2	L2'_c6_temp[b]	Rev2C[a]
HIR-6_T7[a]	CGCTCACTGTATCTCGCGUUUAACGUAACAGCGAU	35	1,2	T3_temp[b]	Rev2A[a]
HIR-6_T8[a]	ACTGTATCTCGCGUUUAACGUAACAGCGAU	30	1,2	T3_temp[b]	Rev2B[a]
HIR-6_T9[a]	CGCTCACTGTATCTCGCGUUUAACGUAACAGCGAUGUCUC	40	1,2	T2_temp[b]	Rev2A[a]
HIR-6_M1[a]	CATGACGCTCACTGTATCTCGCGTTTAACGUAACAGCGAUGUCUCUCAUGAUGUC	55	1,2	L2'_c6_temp[b]	Rev2D[a]
HIR-6_M2[a]	CATGACGCTCACTGTATCTCGCGUUUAACGUAACAGCGAUGTCTCTCATGATGTC	55	1,2	L2'_c6_temp[b]	HIR-6_T3[a]

1 = dA^{AIn}TP; 2 = dU^{EPh}TP; 3 = dA^{EIn}TP; 4 = dU^{APh}TP; 5 = dA^{APh}TP; [a] 5'-Cy5; [b] 5'-Biotin; [c]mutagenized (21%) part underlined; modified nucleotide positions highlighted in red.

2. Chemical synthesis and compound characterizations

All nucleobase-modified dNTPs used in this study were synthesized according to published protocols.[1]

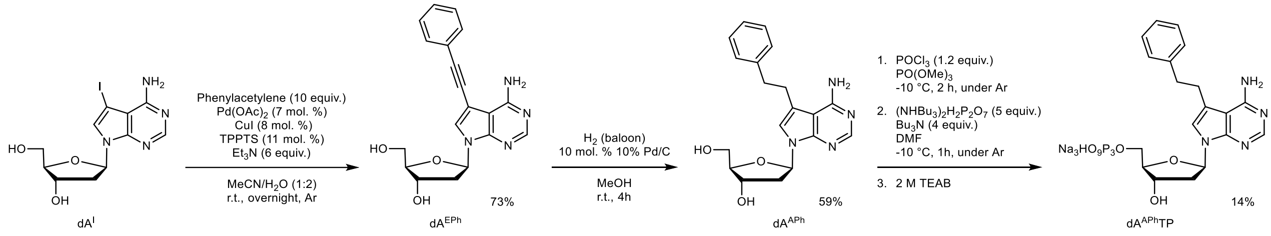

Supplementary Figure 1: Reaction pathway for synthesizing phenyl-modified dA^{APh}TP.

2.1 Sonogashira cross-coupling reaction

1:2 mixture of $MeCN/H_2O$ was added through a septum to an argon-purged flask containing dA^{I} (1 equiv.), TPPTS (11 mol%), CuI (8 mol%), and $Pd(OAc)_2$ (7 mol%), followed by the addition of Phenylacetylene (10 equiv.) and TEA (6 equiv.) (Supplementary Fig. 1). The reaction mixture was stirred at r.t. overnight and then evaporated under vacuum. The product was purified by FLC chromatography using DCM/MeOH (0-30%) as the eluent, followed by evaporation under vacuum to obtain a brown solid with a 73% yield.

7-(2-Phenyl-1-ethyn-1-yl)-2´-deoxyadenosine (dA^{EPh})

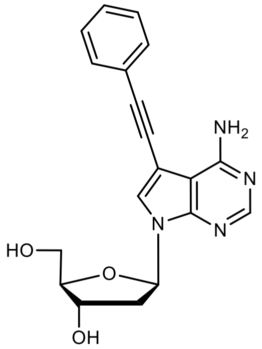

^{1}H NMR (401 MHz, DMSO-d_6): δ 2.21 (ddd, 1H, J_{gem} = 13.1, $J_{2'a,1'}$ = 6.0, $J_{2'a,3'}$ = 2.8, H-2'a); 2.50 (ddd, 1H, J_{gem} = 13.1, $J_{2'b,1'}$ = 8.1, $J_{2'b,3'}$ = 5.6, H-2'b); 3.53, 3.60 (2 × ddd, 2 ×

1H, J_{gem} = 11.7, $J_{5',OH}$ = 5.6, $J_{5',4'}$ = 4.4, H-5'); 3.84 (td, 1H, $J_{4',5'}$ = 4.4, $J_{4',3'}$ = 2.5, H-4'); 4.36 (dddd, 1H, $J_{3',2'}$ = 5.6, 2.8, $J_{3',OH}$ = 4.1, $J_{3',4'}$ = 2.5, H-3'); 5.08 (t, 1H, $J_{OH,5'}$ = 5.6, OH-5'); 5.29 (d, 1H, $J_{OH,3'}$ = 4.1, OH-3'); 6.52 (dd, 1H, $J_{1',2'}$ = 8.1, 6.0, H-1'); 6.72 (bs, 2H, NH_2); 7.39 – 7.45 (m, 3H, H-*m,p*-Ph); 7.56 – 7.60 (m, 2H, H-*o*-Ph); 7.89 (s, 1H, H-6); 8.16 (s, 1H, H-2).

^{13}C NMR (101 MHz, DMSO-d_6): δ 39.92 (CH_2-2'); 61.88 (CH_2-5'); 70.96 (CH-3'); 83.06 (deaza*A*-**C**≡C-Ph); 83.22 (CH-1'); 87.58 (CH-4'); 91.09 (deaza*A*-C≡**C**-Ph); 94.68 (C-5); 102.07 (C-4a); 122.53 (C-*i*-Ph); 126.82 (CH-6); 128.50 (CH-*p*-Ph); 128.70 (CH-*m*-Ph); 131.11 (CH-*o*-Ph); 149.42 (C-7a); 152.82 (CH-2); 157.59 (C-4).

HRMS (m/z): $[M]^+$ calcd. for $C_{19}H_{19}O_3N_4$: 351.14517; found: 351.14513.

2.2 Catalytic hydrogenation

MeOH was added through a septum to an argon-purged flask containing dA^{EPh} (1 equiv.), 10% Pd/C (10 mol%). The flask was then vacuumed and filled with H_2 atmosphere (balloon). The reaction mixture was stirred at r.t. for 4 hours and then evaporated under vacuum. The product was purified by FLC chromatography using DCM/MeOH (0-30%) as the eluent, followed by evaporation under vacuum to yield a white solid with 59% yield.

7-(2-Phenylethyl)-2´-deoxyadenosine (dA^{APh})

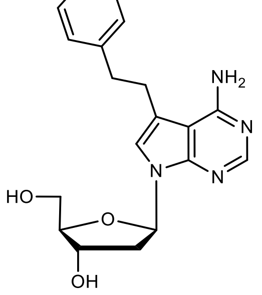

^{1}H NMR (401 MHz, DMSO-d_6): δ 2.09 (ddd, 1H, J_{gem} = 13.1, $J_{2'a,1'}$ = 5.9, $J_{2'a,3'}$ = 2.6, H-2'a); 2.42 (ddd, 1H, J_{gem} = 13.1, $J_{2'b,1'}$ = 8.4, $J_{2'b,3'}$ = 5.8, H-2'b); 2.84-2.96 (m, 2H, deazaA-CH_2C**H_2**-Ph); 2.99-3.14 (m, 2H, deazaA-C**H_2**CH_2-Ph); 3.47 (ddd, 1H, J_{gem} = 11.6, $J_{5'b,OH}$

= 6.0, $J_{5'b,4'}$ = 4.6, H-5'b); 3.54 (ddd, 1H, J_{gem} = 11.6, $J_{5'a,OH}$ = 5.2, $J_{5'a,4'}$ = 4.6, H-5'a); 3.79 (td, 1H, $J_{4',5'}$ = 4.6, $J_{4',3'}$ = 2.5, H-4'); 4.30 (m, 1H, H-3'); 5.07 (dd, 1H, $J_{OH,5'}$ = 6.0, 5.2, OH-5'); 5.23 (d, 1H, $J_{OH,3'}$ = 4.1, OH-3'); 6.47 (dd, 1H, $J_{1',2'}$ = 8.4, 5.9, H-1'); 6.57 (bs, 2H, NH_2); 7.10 (t, 1H, $J_{6,CH2}$ = 1.2, H-6); 7.18 (m, 1H, H-*p*-Ph); 7.25 – 7.31 (m, 4H, H-*o*,*m*-Ph); 8.02 (s, 1H, H-2).

^{13}C NMR (101 MHz, DMSO-d_6): δ 27.61 (deazaA-**C**H_2CH_2-Ph); 35.85 (deazaA-CH_2**C**H_2-Ph); 39.51 (CH_2-2'); 62.17 (CH_2-5'); 71.12 (CH-3'); 82.73 (CH-1'); 87.11 (CH-4'); 102.20 (C-4a); 114.75 (C-5); 118.74 (CH-6); 125.78 (CH-*p*-Ph); 128.14 (CH-*m*-Ph); 128.52 (CH-*o*-Ph); 141.58 (C-*i*-Ph); 150.36 (C-7a); 151.32 (CH-2); 157.77 (C-4).

HRMS (m/z): $[M]^+$ calcd. for $C_{19}H_{23}O_3N_4$: 355.17647; found: 355.17642.

2.3 Triphosphorylation

$PO(OMe)_3$ (1 mL) was added through a septum to an argon-purged flask containing dA^{APh} (1 equiv.), followed by the dropwise addition of $POCl_3$ (1.2 equiv.) at -10 °C (ice bath + NaCl). The reaction mixture was stirred for two hours at -10 °C. Then, the ice-cooled mixture containing a solution of $(NHBu_3)_2H_2P_2O_7$ (5 equiv.) and Bu_3N (4 equiv.) in 1 mL of dry DMF was added dropwise. The mixture was stirred for an additional hour at -10 °C. The reaction was quenched with 5 mL of aqueous 2 M TEAB (triethylammonium bicarbonate). Solvents were evaporated under vacuum and co-distilled with water three times. The product was purified using HPLC on a C18 column, employing a linear gradient from 0.1 M TEAB in water to 0.1 M TEAB in a 1:1 mixture of water and methanol as the eluent. Conversion to the sodium salt was achieved via ion exchange resin Dowex 50WX8, followed by freeze-drying from water, resulting in a white solid product with a 14% yield.

7-(2-Phenylethyl)-2´-deoxyadenosine triphosphate (dA^{APh}TP)

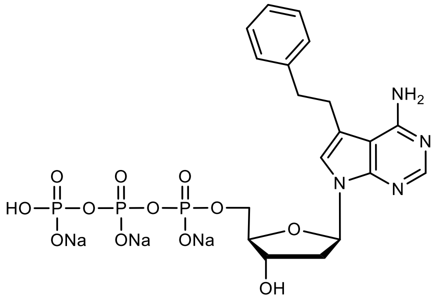

^{1}H NMR (500 MHz, D_2O): δ 2.36 (ddd, 1H, J_{gem} = 14.1, $J_{2'a,1'}$ = 6.3, $J_{2'a,3'}$ = 3.4, H-2'a); 2.55 (ddd, 1H, J_{gem} = 14.1, $J_{2'b,1'}$ = 8.0, $J_{2'b,3'}$ = 6.4, H-2'b); 2.92-3.04 (m, 2H, deaza*A*-$CH_2C\mathbf{H_2}$-Ph); 3.05-3.15 (m, 2H, deazaA-$C\mathbf{H_2}CH_2$-Ph); 4.05 (ddd, 1H, J_{gem} = 11.1, $J_{H,P}$ = 6.0, $J_{5'b,4'}$ = 4.5, H-5'b); 4.11 (ddd, 1H, J_{gem} = 11.1, $J_{H,P}$ = 6.6, $J_{5'a,4'}$ = 4.5, H-5'a); 4.18 (tdd, 1H, $J_{4',5'}$ = 4.5, $J_{4',3'}$ = 3.4, $J_{H,P}$ = 1.2, H-4'); 4.68 (dt, 1H, $J_{3',2'}$ = 6.4, 3.4, $J_{3',4'}$ = 3.4, H-3'); 6.57 (dd, 1H, $J_{1',2'}$ = 8.0, 6.2, H-1'); 7.08 (s, 1H, H-6); 7.14-7.18 (m, 2H, H-*o*-Ph); 7.24 (m, 1H, H-*p*-Ph); 7.27 – 7.31 (m, 2H, H-*m*-Ph); 8.08 (s, 1H, H-2).

^{13}C NMR (126 MHz, D_2O): δ 28.24 (deazaA-$\mathbf{C}H_2CH_2$-Ph); 36.83 (deazaA-$CH_2\mathbf{C}H_2$-Ph); 38.68 (CH_2-2'); 66.19 (d, $J_{C,P}$ = 5.7, CH_2-5'); 71.74 (CH-3'); 83.10 (CH-1'); 85.61 (d, $J_{C,P}$ = 8.7, CH-4'); 103.55 (C-4a); 116.82 (C-5); 120.24 (CH-6); 126.90 (CH-*p*-Ph); 129.18 (CH-*m*-Ph); 129.54 (CH-*o*-Ph); 142.12 (C-*i*-Ph); 150.51 (C-7a); 151.82 (CH-2); 158.32 (C-4).

^{31}P NMR (202 MHz, D_2O): δ -21.26 (dd, J = 20.4, 19.5, P_β); -10.31 (d, J = 19.5, P_α); -5.26 (d, J = 20.4, P_γ).

HRMS (m/z): $[M]^-$ calcd. for $C_{19}H_{21}O_{12}N_4Na_3P_3$: 659.00674; found: 659.00662.

3. Enzymatic synthesis of DNA libraries

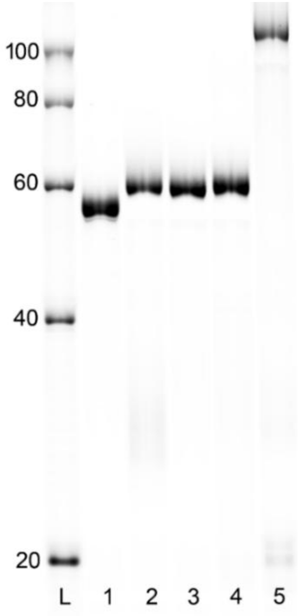

Supplementary Figure 2. Cy5 scan of denaturing PAGE analysis of synthesized and HPLC-purified libraries. Cy5-labeled ssDNA ladder (lane L); L1 library (lane 1); L2 library (lane 2); L3 library (lane 3); L2' library (lane 4); L4 library (lane 5).

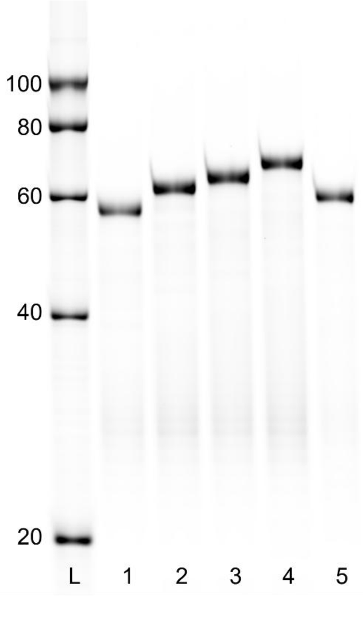

Supplementary Figure 3. Cy5 scan of dPAGE analysis of synthesized and HPLC-purified dually-modified libraries. Cy5-labeled ssDNA ladder (lane L); L2'_N13 (lane 1); L2'_N17 (lane 2); L2'_N19 (lane 3); L2'_N22 (lane 4); L5 (lane 5).

4. Insulin receptors as targets

4.1 Amino acid sequence alignments of HIR with CIR and DIR

Sequence alignment performed with BLAST.[2]

Query = HIR, Accession # P06213-2; subject = CIR, Accession # A0A2I2UE37.

Score	Expect	Method	Identities	Positives	Gaps
2736 bits(7093)	0.0	Compositional matrix adjust.	1306/1355(96%)	1332/1355(98%)	1/1355(0%)

```
Query  1    HLYPGEVCPGMDIRNNLTRLHELENCSVIEGHLQILLMFKTRPEDFRDLSFPKLIMITDY  60
            HLYPGEVCPGMDIRNNLTRLHEL NCSVIEGHLQILLMFKTRPEDFRDLSFPKL+MITDY
Sbjct  1    HLYPGEVCPGMDIRNNLTRLHELANCSVIEGHLQILLMFKTRPEDFRDLSFPKLVMITDY  60

Query  61   LLLFRVYGLESLKDLFPNLTVIRGSRLFFNYALVIFEMVHLKELGLYNLMNITRGSVRIE  120
            LLLFRVYGLESLKDLFPNLTVIRGSRLFFNYALV+FEMVHLKELGLY+LMNITRGSVRIE
Sbjct  61   LLLFRVYGLESLKDLFPNLTVIRGSRLFFNYALVVFEMVHLKELGLYSLMNITRGSVRIE  120

Query  121  KNNELCYLATIDWSRILDSVEDNYIVLNKDDNEECGDICPGTAKGKTNCPATVINGQFVE  180
            KNNELCYLATIDWSRILDSVEDNYIVLNKDDNEECGDICPGTAKGKTNCPATVINGQFVE
Sbjct  121  KNNELCYLATIDWSRILDSVEDNYIVLNKDDNEECGDICPGTAKGKTNCPATVINGQFVE  180

Query  181  RCWTHSHCQKVCPTICKSHGCTAEGLCCHSECLGNCSQPDDPTKCVACRNFYLDGRCVET  240
            RCWTH HCQKVCPT+CKSHGCTA+GLCCHSECLGNCS+PDDPTKCVACRNFYLDGRCVET
Sbjct  181  RCWTHGHCQKVCPTVCKSHGCTADGLCCHSECLGNCSEPDDPTKCVACRNFYLDGRCVET  240

Query  241  CPPPYYHFQDWRCVNFSFCQDLHHKCKNSRRQGCHQYVIHNNKCIPECPSGYTMNSSNLL  300
            CPPPYYHFQDWRCVNFSFC+DLH+KCKNSRRQGCHQYVIHNN+CIPECPSGYTMNSSNL+
Sbjct  241  CPPPYYHFQDWRCVNFSFCRDLHNKCKNSRRQGCHQYVIHNNRCIPECPSGYTMNSSNLM  300

Query  301  CTPCLGPCPKVCHLLEGEKTIDSVTSAQELRGCTVINGSLIINIRGGNNLAAELEANLGL  360
            CTPCLGPCPKVCH+LEGEKTIDSVTSAQELRGCTVINGSLIINIRGGNNLAAELEANLGL
Sbjct  301  CTPCLGPCPKVCHILEGEKTIDSVTSAQELRGCTVINGSLIINIRGGNNLAAELEANLGL  360

Query  361  IEEISGYLKIRRSYALVSLSFFRKLRLIRGETLEIGNYSFYALDNQNLRQLWDWSKHNLT  420
            IEEISGYLKIRRSYALVSLSFFRKLRLIRGETLEIGNYSFYALDNQNLRQLWDWSKHNLT
Sbjct  361  IEEISGYLKIRRSYALVSLSFFRKLRLIRGETLEIGNYSFYALDNQNLRQLWDWSKHNLT  420

Query  421  ITQGKLFFHYNPKLCLSEIHKMEEVSGTKGRQERNDIALKTNGDQASCENELLKFSYIRT  480
            ITQGKLFFHYNPKLCLSEIHKMEEVSGTKGRQERNDIALKTNGDQASCENELLKFSYIRT
Sbjct  421  ITQGKLFFHYNPKLCLSEIHKMEEVSGTKGRQERNDIALKTNGDQASCENELLKFSYIRT  480

Query  481  SFDKILLRWEPYWPPDFRDLLGFMLFYKEAPYQNVTEFDGQDACGSNSWTVVDIDPPLRS  540
            S+DKILL+WEPYWPPDFRDLLGFMLFYKEAPYQNVTEFDGQDACGSNSWTVVDIDPPLRS
Sbjct  481  SYDKILLKWEPYWPPDFRDLLGFMLFYKEAPYQNVTEFDGQDACGSNSWTVVDIDPPLRS  540

Query  541  NDPKSQNHPGWLMRGLKPWTQYAIFVKTLVTFSDERRTYGAKSDIIYVQTDATNPSVPLD  600
            NDPKSQNHPGWLMRGLKPWTQYAIFVKTLVTFSDERRTYGAKSDIIYVQTDATNPSVPLD
Sbjct  541  NDPKSQNHPGWLMRGLKPWTQYAIFVKTLVTFSDERRTYGAKSDIIYVQTDATNPSVPLD  600

Query  601  PISVSNSSSQIILKWKPPSDPNGNITHYLVFWERQAEDSELFELDYCLKGLKLPSRTWSP  660
            PISVSNSSSQIILKWKPPSDPNGNITHYLVFWERQAEDSEL+ELDYCLKGLKLPSRTWSP
Sbjct  601  PISVSNSSSQIILKWKPPSDPNGNITHYLVFWERQAEDSELYELDYCLKGLKLPSRTWSP  660

Query  661  PFESEDSQKHNQSEYEDSAGECCSCPKTDSQILKELEESSFRKTFEDYLHNVVFVPRKTS  720
            PFESE SQK NQSEYE+SAGECCSCPKTDSQILKELEESSFRKTFEDYLHNVVFVPRK+
Sbjct  661  PFESEGSQKRNQSEYEESAGECCSCPKTDSQILKELEESSFRKTFEDYLHNVVFVPRKSF  720

Query  721  SGTGAEDPRPSRKRRSLGDVGNVTVAVPTVAAFPNTSSTSVPTSPEEHRPFEKVVNKESL  780
            S  GAED RPSRKRR+L D GNVT AVPTV  FPNTSS SVPTSPEEH+PFEKVVNKESL
Sbjct  721  SSPGAEDSRPSRKRRALADAGNVTAAVPTVPGFPNTSS-SVPTSPEEHKPFEKVVNKESL  779
```

```
Query  781   VISGLRHFTGYRIELQACNQDTPEERCSVAAYVSARTMPEAKADDIVGPVTHEIFENNVV  840
             VISGLRHFTGYRIELQACNQD PEERCSVAAYVSARTMPEAKADDIVGPVTHEIFENNVV
Sbjct  780   VISGLRHFTGYRIELQACNQDAPEERCSVAAYVSARTMPEAKADDIVGPVTHEIFENNVV  839

Query  841   HLMWQEPKEPNGLIVLYEVSYRRYGDEELHLCVSRKHFALERGCRLRGLSPGNYSVRIRA  900
             HLMWQEPKEPNGLIVLYEVSYRRYGDEELHLCVSR+HFALERGCRLRGL PGNYSVR+RA
Sbjct  840   HLMWQEPKEPNGLIVLYEVSYRRYGDEELHLCVSRRHFALERGCRLRGLPPGNYSVRVRA  899

Query  901   TSLAGNGSWTEPTYFYVTDYLDVPSNIAKIIIGPLIFVFLFSVVIGSIYLFLRKRQPDGP  960
             TSLAGNGSWTE TYFYVTDYLDVPSNIAKIIIGPLIFVFLFSVVIGSIYLFLRKRQPDGP
Sbjct  900   TSLAGNGSWTEATYFYVTDYLDVPSNIAKIIIGPLIFVFLFSVVIGSIYLFLRKRQPDGP  959

Query  961   LGPLYASSNPEYLSASDVFPCSVYVPDEWEVSREKITLLRELGQGSFGMVYEGNARDIIK  1020
             LGPLYASSNPEYLSASDVFPCSVYVPDEWEV REKITLLRELGQGSFGMVYEGNARDI+K
Sbjct  960   LGPLYASSNPEYLSASDVFPCSVYVPDEWEVPREKITLLRELGQGSFGMVYEGNARDIVK  1019

Query  1021  GEAETRVAVKTVNESASLRERIEFLNEASVMKGFTCHHVVRLLGVVSKGQPTLVVMELMA  1080
             GEAETRVAVKTVNESASLRERIEFLNEASVMKGFTCHHVVRLLGVVSKGQPTLVVMELM
Sbjct  1020  GEAETRVAVKTVNESASLRERIEFLNEASVMKGFTCHHVVRLLGVVSKGQPTLVVMELMV  1079

Query  1081  HGDLKSYLRSLRPEAENNPGRPPPTLQEMIQMAAEIADGMAYLNAKKFVHRDLAARNCMV  1140
             HGDLKSYLRSLRPEAENNPGRPPPTLQEMIQMAAEIADGMAYLNAKKFVHRDLAARNCMV
Sbjct  1080  HGDLKSYLRSLRPEAENNPGRPPPTLQEMIQMAAEIADGMAYLNAKKFVHRDLAARNCMV  1139

Query  1141  AHDFTVKIGDFGMTRDIYETDYYRKGGKGLLPVRWMAPESLKDGVFTTSSDMWSFGVVLW  1200
             AHDFTVKIGDFGMTRDIYETDYYRKGGKGLLPVRWMAPESLKDGVFTTSSDMWSFGVVLW
Sbjct  1140  AHDFTVKIGDFGMTRDIYETDYYRKGGKGLLPVRWMAPESLKDGVFTTSSDMWSFGVVLW  1199

Query  1201  EITSLAEQPYQGLSNEQVLKFVMDGGYLDQPDNCPERVTDLMRMCWQFNPKMRPTFLEIV  1260
             EITSLAEQPYQGLSNEQVLKFVMDGGYLDQPDNCPERVTDLM MCWQFNPKMRPTFLEIV
Sbjct  1200  EITSLAEQPYQGLSNEQVLKFVMDGGYLDQPDNCPERVTDLMHMCWQFNPKMRPTFLEIV  1259

Query  1261  NLLKDDLHPSFPEVSFFHSEENKAPESEELEMEFEDMENVPLDRSSHCQREEAGGRDGGS  1320
             +LLKDDLHPSFPEVSFFHSEENKAPESEELEMEFEDME+VPLDR+SH QREEAGGRDG S
Sbjct  1260  DLLKDDLHPSFPEVSFFHSEENKAPESEELEMEFEDMESVPLDRASHSQREEAGGRDGVS  1319

Query  1321  SLGFKRSYEEHIPYTHMNGGKKNGRILTLPRSNPS  1355
             SLG KR+YE+HIPYTHMNGGKKNGRILTLPRSNPS
Sbjct  1320  SLGLKRNYEDHIPYTHMNGGKKNGRILTLPRSNPS  1354
```

Supplementary Figure 4. Amino acid sequence alignment of HIR and CIR. Amino acid sequences are presented in single-letter code, non-identical residues are presented with a gap, and conserved missense mutations are represented with +. Amino acid numbering starts after the signal peptide.

Query = HIR, Accession # P06213-2; subject = DIR, Accession # A0A8I3PWD4.

Score	Expect	Method	Identities	Positives	Gaps
2753 bits(7137)	0.0	Compositional matrix adjust.	1314/1355(97%)	1336/1355(98%)	1/1355(0%)

```
Query  1    HLYPGEVCPGMDIRNNLTRLHELENCSVIEGHLQILLMFKTRPEDFRDLSFPKLIMITDY  60
            HLYPGEVCPGMDIRNNLTRLHEL NCSVIEGHLQILLMFKTRPEDFRDLSFPKLIMITDY
Sbjct  1    HLYPGEVCPGMDIRNNLTRLHELANCSVIEGHLQILLMFKTRPEDFRDLSFPKLIMITDY  60

Query  61   LLLFRVYGLESLKDLFPNLTVIRGSRLFFNYALVIFEMVHLKELGLYNLMNITRGSVRIE  120
            LLLFRVYGLESLKDLFPNLTVIRGSRLFFNYALVIFEMVHLKELGLY+LMNITRGSVRIE
Sbjct  61   LLLFRVYGLESLKDLFPNLTVIRGSRLFFNYALVIFEMVHLKELGLYSLMNITRGSVRIE  120

Query  121  KNNELCYLATIDWSRILDSVEDNYIVLNKDDNEECGDICPGTAKGKTNCPATVINGQFVE  180
            KNNELCYLATIDWSRILDSVEDNYIVLNKDDNEECGDICPGTAKGKTNCPATVINGQFVE
Sbjct  121  KNNELCYLATIDWSRILDSVEDNYIVLNKDDNEECGDICPGTAKGKTNCPATVINGQFVE  180

Query  181  RCWTHSHCQKVCPTICKSHGCTAEGLCCHSECLGNCSQPDDPTKCVACRNFYLDGRCVET  240
            RCWTHSHCQKVCPTICKSHGCTAEGLCCHSECLGNCS+PDDPTKCVACRNFYLDGRCVET
Sbjct  181  RCWTHSHCQKVCPTICKSHGCTAEGLCCHSECLGNCSEPDDPTKCVACRNFYLDGRCVET  240

Query  241  CPPPYYHFQDWRCVNFSFCQDLHHKCKNSRRQGCHQYVIHNNKCIPECPSGYTMNSSNLL  300
            CPPPYYHFQDWRCVNFSFCQDLH+KCKNSRRQGCHQYVIHNNKCIPECPSGYTMNSSNL+
Sbjct  241  CPPPYYHFQDWRCVNFSFCQDLHNKCKNSRRQGCHQYVIHNNKCIPECPSGYTMNSSNLM  300

Query  301  CTPCLGPCPKVCHLLEGEKTIDSVTSAQELRGCTVINGSLIINIRGGNNLAAELEANLGL  360
            CTPCLGPCPKVCH+LEGEKTIDSVTSAQELRGCTV+NGSLIINIRGGNNLAAELEANLGL
Sbjct  301  CTPCLGPCPKVCHILEGEKTIDSVTSAQELRGCTVVNGSLIINIRGGNNLAAELEANLGL  360

Query  361  IEEISGYLKIRRSYALVSLSFFRKLRLIRGETLEIGNYSFYALDNQNLRQLWDWSKHNLT  420
            IEEISGYLKIRRSYALVSLSFFRKLRLIRGETLEIGNYSFYALDNQNLRQLWDWSKHNLT
Sbjct  361  IEEISGYLKIRRSYALVSLSFFRKLRLIRGETLEIGNYSFYALDNQNLRQLWDWSKHNLT  420

Query  421  ITQGKLFFHYNPKLCLSEIHKMEEVSGTKGRQERNDIALKTNGDQASCENELLKFSYIRT  480
            ITQGKLFFHYNPKLCLSEIHKMEEVSGTKGRQERNDIALKTNGDQASCENELLKFSYIRT
Sbjct  421  ITQGKLFFHYNPKLCLSEIHKMEEVSGTKGRQERNDIALKTNGDQASCENELLKFSYIRT  480

Query  481  SFDKILLRWEPYWPPDFRDLLGFMLFYKEAPYQNVTEFDGQDACGSNSWTVVDIDPPLRS  540
            S+DKILL+WEPYWPPDFRDLLGFMLFYKEAPYQNVTEFDGQDACGSNSWTVVDIDPPLRS
Sbjct  481  SYDKILLKWEPYWPPDFRDLLGFMLFYKEAPYQNVTEFDGQDACGSNSWTVVDIDPPLRS  540

Query  541  NDPKSQNHPGWLMRGLKPWTQYAIFVKTLVTFSDERRTYGAKSDIIYVQTDATNPSVPLD  600
            NDPKSQNHPGWLMRGLKPWTQYAIFVKTLVTFSDERRTYGAKSDIIYVQTDATNPSVPLD
Sbjct  541  NDPKSQNHPGWLMRGLKPWTQYAIFVKTLVTFSDERRTYGAKSDIIYVQTDATNPSVPLD  600

Query  601  PISVSNSSSQIILKWKPPSDPNGNITHYLVFWERQAEDSELFELDYCLKGLKLPSRTWSP  660
            PISVSNSSSQIILKWKPPSDPNGNITHYLVFWERQAEDSEL+ELDYCLKGLKLPSRTWSP
Sbjct  601  PISVSNSSSQIILKWKPPSDPNGNITHYLVFWERQAEDSELYELDYCLKGLKLPSRTWSP  660

Query  661  PFESEDSQKHNQSEYEDSAGECCSCPKTDSQILKELEESSFRKTFEDYLHNVVFVPRKTS  720
            PFE+E SQKHNQSEYE+SAGECCSCPKTDSQILKELEESSFRKTFEDYLHNVVFVPRK+S
Sbjct  661  PFEAEGSQKHNQSEYEESAGECCSCPKTDSQILKELEESSFRKTFEDYLHNVVFVPRKSS  720

Query  721  SGTGAEDPRPSRKRRSLGDVGNVTVAVPTVAAFPNTSSTSVPTSPEEHRPFEKVVNKESL  780
            S  GAED RPSRKRR+L D GNVT A+PTV  FPNT STS PTSPEEH+PFEKVVNKESL
Sbjct  721  SSPGAEDTRPSRKRRALSDAGNVTAAIPTVPGFPNT-STSTPTSPEEHKPFEKVVNKESL  779
```

```
Query  781   VISGLRHFTGYRIELQACNQDTPEERCSVAAYVSARTMPEAKADDIVGPVTHEIFENNVV  840
             VISGLRHFTGYRIELQACNQD+PEERCSVAAYVSARTMPEAKADDIVGPVTHEIFENNVV
Sbjct  780   VISGLRHFTGYRIELQACNQDSPEERCSVAAYVSARTMPEAKADDIVGPVTHEIFENNVV  839

Query  841   HLMWQEPKEPNGLIVLYEVSYRRYGDEELHLCVSRKHFALERGCRLRGLSPGNYSVRIRA  900
             HLMWQEPKEPNGLIVLYEVSYRRYGDEELHLCVSR+HFALERGCRLRGL PGNYSVR+RA
Sbjct  840   HLMWQEPKEPNGLIVLYEVSYRRYGDEELHLCVSRRHFALERGCRLRGLPPGNYSVRVRA  899

Query  901   TSLAGNGSWTEPTYFYVTDYLDVPSNIAKIIIGPLIFVFLFSVVIGSIYLFLRKRQPDGP  960
             TSLAGNGSWTE TYFYVTDYLDVPSNIAKIIIGPLIFVFLFSVVIGSIYLFLRKRQPDGP
Sbjct  900   TSLAGNGSWTEATYFYVTDYLDVPSNIAKIIIGPLIFVFLFSVVIGSIYLFLRKRQPDGP  959

Query  961   LGPLYASSNPEYLSASDVFPCSVYVPDEWEVSREKITLLRELGQGSFGMVYEGNARDIIK  1020
             LGPLYASSNPEYLSASDVFPCSVYVPDEWEV REKITLLRELGQGSFGMVYEGNARDI+K
Sbjct  960   LGPLYASSNPEYLSASDVFPCSVYVPDEWEVPREKITLLRELGQGSFGMVYEGNARDIVK  1019

Query  1021  GEAETRVAVKTVNESASLRERIEFLNEASVMKGFTCHHVVRLLGVVSKGQPTLVVMELMA  1080
             GE ETRVAVKTVNESASLRERIEFLNEASVMKGFTCHHVVRLLGVVSKGQPTLVVMELMA
Sbjct  1020  GEPETRVAVKTVNESASLRERIEFLNEASVMKGFTCHHVVRLLGVVSKGQPTLVVMELMA  1079

Query  1081  HGDLKSYLRSLRPEAENNPGRPPPTLQEMIQMAAEIADGMAYLNAKKFVHRDLAARNCMV  1140
             HGDLKSYLRSLRPEAENNPGRPPPTLQEMIQMAAEIADGMAYLNAKKFVHRDLAARNCMV
Sbjct  1080  HGDLKSYLRSLRPEAENNPGRPPPTLQEMIQMAAEIADGMAYLNAKKFVHRDLAARNCMV  1139

Query  1141  AHDFTVKIGDFGMTRDIYETDYYRKGGKGLLPVRWMAPESLKDGVFTTSSDMWSFGVVLW  1200
             AHDFTVKIGDFGMTRDIYETDYYRKGGKGLLPVRWMAPESLKDGVFTTSSDMWSFGVVLW
Sbjct  1140  AHDFTVKIGDFGMTRDIYETDYYRKGGKGLLPVRWMAPESLKDGVFTTSSDMWSFGVVLW  1199

Query  1201  EITSLAEQPYQGLSNEQVLKFVMDGGYLDQPDNCPERVTDLMRMCWQFNPKMRPTFLEIV  1260
             EITSLAEQPYQGLSNEQVLKFVMDGGYLDQPDNCPERVTDLMRMCWQFNPKMRPTFLEIV
Sbjct  1200  EITSLAEQPYQGLSNEQVLKFVMDGGYLDQPDNCPERVTDLMRMCWQFNPKMRPTFLEIV  1259

Query  1261  NLLKDDLHPSFPEVSFFHSEENKAPESEELEMEFEDMENVPLDRSSHCQREEAGGRDGGS  1320
             +LLKDDLHPSFPEVSFFHSEENKAPESEELEMEFEDME+VPLDR+SH QREEAGGRD GS
Sbjct  1260  DLLKDDLHPSFPEVSFFHSEENKAPESEELEMEFEDMESVPLDRASHSQREEAGGRDAGS  1319

Query  1321  SLGFKRSYEEHIPYTHMNGGKKNGRILTLPRSNPS  1355
             SLG KRSYEEHIPYTHMNGGKKNGRILTLPRSNPS
Sbjct  1320  SLGLKRSYEEHIPYTHMNGGKKNGRILTLPRSNPS  1354
```

Supplementary Figure 5. Amino acid sequence alignment of HIR and DIR. Amino acid sequences are presented in single-letter code, non-identical residues are presented with a gap, and conserved missense mutations are represented with +. Amino acid numbering starts after the signal peptide.

5. Aptamer selections

5.1 Single-round aptamer selection

Supplementary Table 3. HIR single-round selection conditions.

Partition step	HIR/DIR (nM)	Library (pmol)	Protein competitors	Anionic competitor	Interaction volume (µL)
1st	1000	300	Yes	No	100
2nd	150	~5×10^{-5}	No	Yes	100

5.2 Selection for covariation analysis

Supplementary Table 4. Selection conditions used in covariation analysis.

Cycle	HIR (nM)	L4 library (pmol)	Protein competitors	Anionic competitor (µL)	Interaction volume (µL)
1st	100	200	Yes	800	100
2nd	58	7	Yes	800	100
3rd	15	7	Yes	800	100
4th	7	7	Yes	1600	100

6. Next-Generation Sequencing (NGS) and data analysis

6.1 Sample preparation and NGS

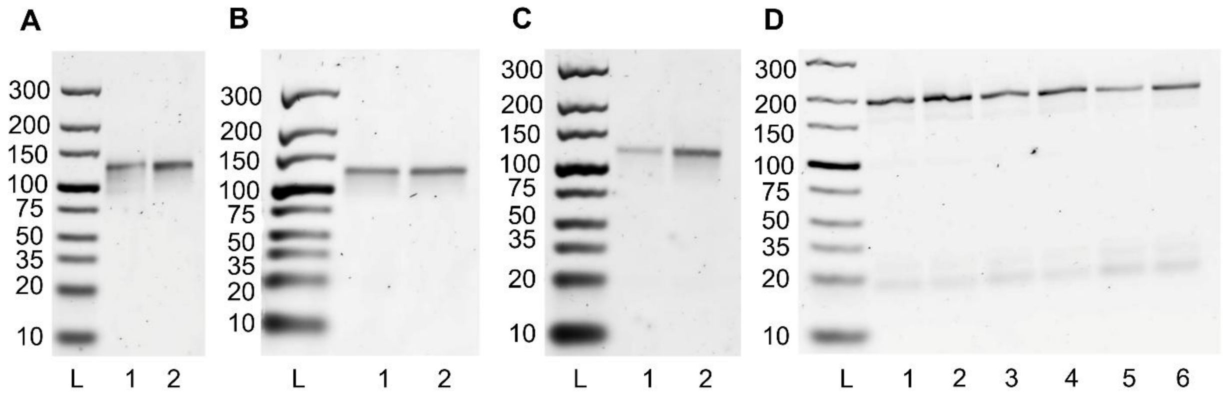

Supplementary Figure 6. GelRed-stained native agarose gel of PCR-amplified sequences from SRSs. dsDNA ladder (lanes L). A), B) and C) Adapter PCR of initial libraries (lanes 1) and recovered sequences (lanes 2) from selections using L1, L2 and L3, respectively. D) Index PCR

of initial libraries (odd lanes) and recovered sequences (even lanes) from selections using L1, L2 and L3, respectively.

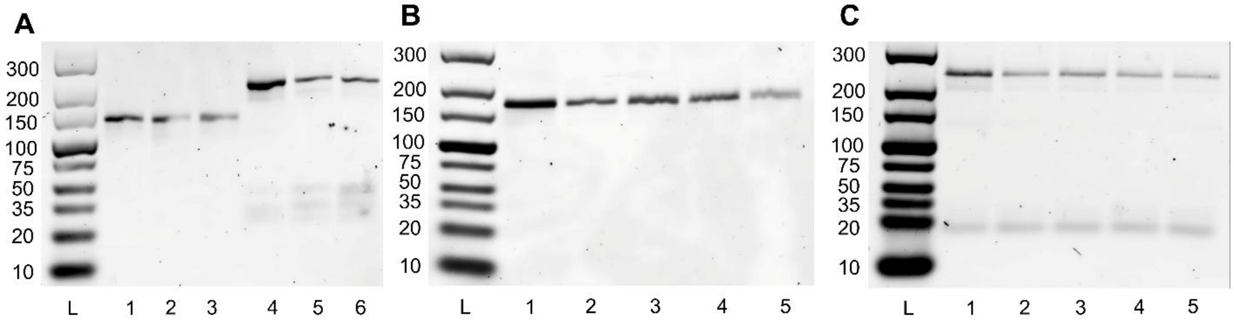

Supplementary Figure 7. GelRed-stained native agarose gel of PCR-amplified sequences from SRS and covariation selection. dsDNA Ladder (lanes L). A) Adapter PCR (lanes 1-3) and index PCR (lanes 4-6) from selection using L2' library. Initial library (lanes 1 and 4); 1st partition step (lanes 2 and 5); 2nd partition step (lanes 3 and 6). B) Adapter and C) index PCR from covariation selection. Initial library (lanes 1); 1st round (lanes 2); 2nd round (lanes 3); 3rd round (lanes 4) and 4th round (lanes 5).

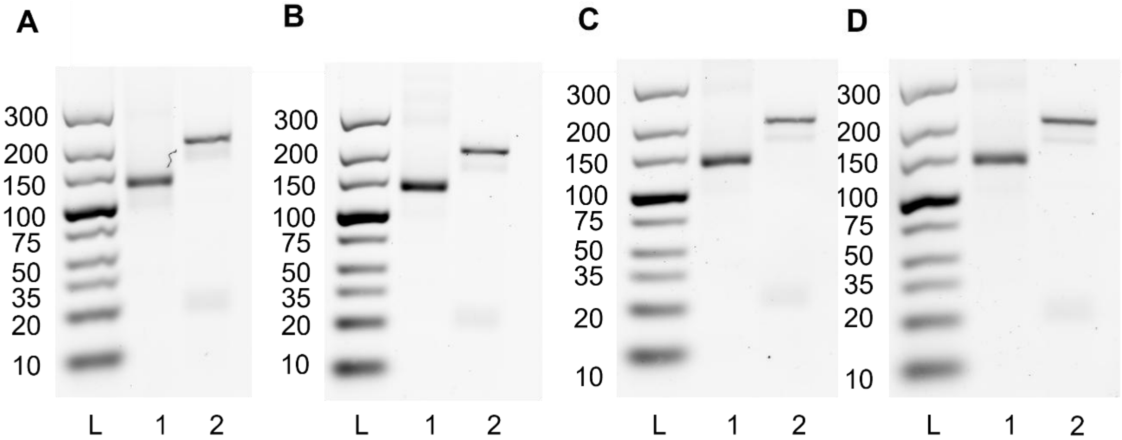

Supplementary Figure 8. GelRed-stained native agarose gel of PCR-amplified recovered sequences from SRSs. dsDNA Ladder (lanes L). Adapter PCR (lanes 1) and index PCR (lanes 2) of recovered sequences from selections using L2'_N13 (A), L2'_N17 (B), L2'_N19 (C) and L2'_N22 (D) library.

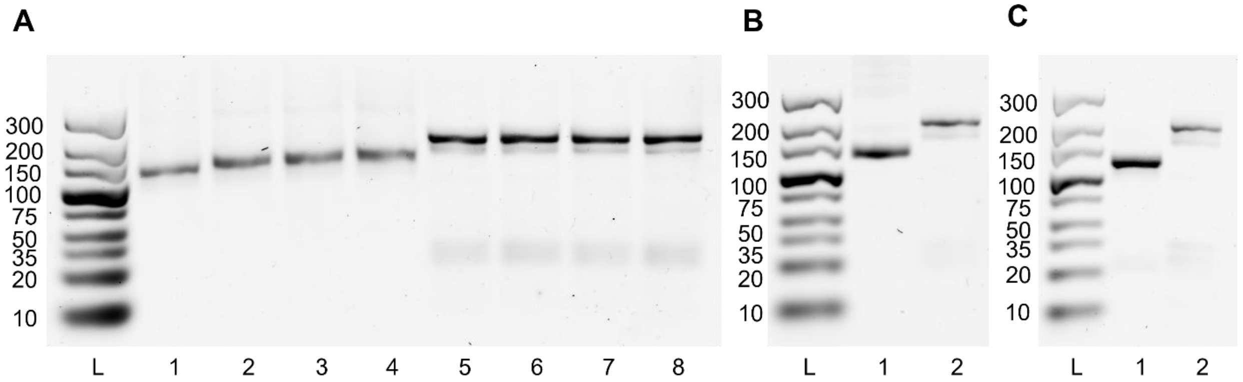

Supplementary Figure 9. GelRed-stained native agarose gel of PCR-amplified recovered sequences from SRSs. dsDNA Ladder (lanes L). A) Adapter PCR (lanes 1-4) and index PCR (lanes 1-4) of L2'_N13 (lanes 1 and 5), L2'_N17 (lanes 2 and 6), L2'_N19 (lanes 3 and 7) and L2'_N22 (lanes 4 and 8) initial libraries. B) and C) Adapter PCR (lanes 1) and index PCR (lanes 2) of recovered sequences from selection using L5 (B) and L5 initial library (C).

Supplementary Table 5. NGS data of the SRS pools.

Selection round	Total raw sequences	Sequences used for distributions of sequence abundances
L1 - initial library	62,718,779	25,000,000
L1	61,078,238	25,000,000
L2 - initial library	53,948,409	25,000,000
L2	44,945,590	25,000,000
L3 - initial library	58,320,126	25,000,000
L3	43,383,785	25,000,000
L2' - initial library	31,439,601	28,635,776
L2' 1st partition step	32,141,588	29,407,599
L2' 2nd partition step	31,505,267	28,647,255
L2'_N13 – initial library	31,791,499	20,000,000
L2' - initial library	31,439,601	20,000,000
L2'_N17 – initial library	33,013,069	20,000,000
L2'_N19 – initial library	30,018,021	20,000,000
L2'_N22 – initial library	25,758,998	20,000,000
L2'_N13	24,471,797	20,000,000
L2' 2nd partition step	31,505,267	20,000,000
L2'_N17	23,883,446	20,000,000
L2'_N19	29,680,003	20,000,000
L2'_N22	25,713,585	20,000,000
L4 – initial library	10,273,117	9,188,802
L4 4th round	7,471,308	6,618,962
L5 – initial library	31,814,874	29,214,147
L5	30,671,848	28,401,356

6.2 Distributions of sequence abundances

To evaluate the impact of each library on the SRS, each sequenced pool was normalized to its initial library and used to generate enrichment profiles that display the distributions of sequence abundances. These distributions illustrate the performance of phenyl- and indol-modified dNTPs in enriching binding sequences except for the non-modified L1.

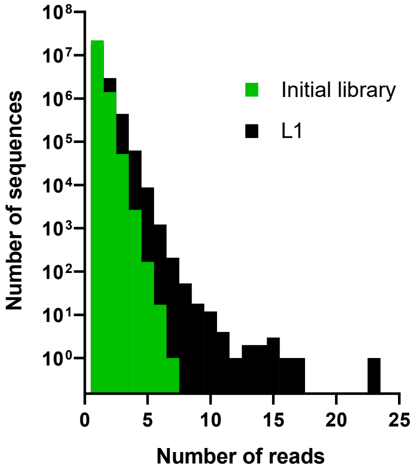

Supplementary Figure 10. Distributions of sequence abundances of L1 library after SRS and its initial library.

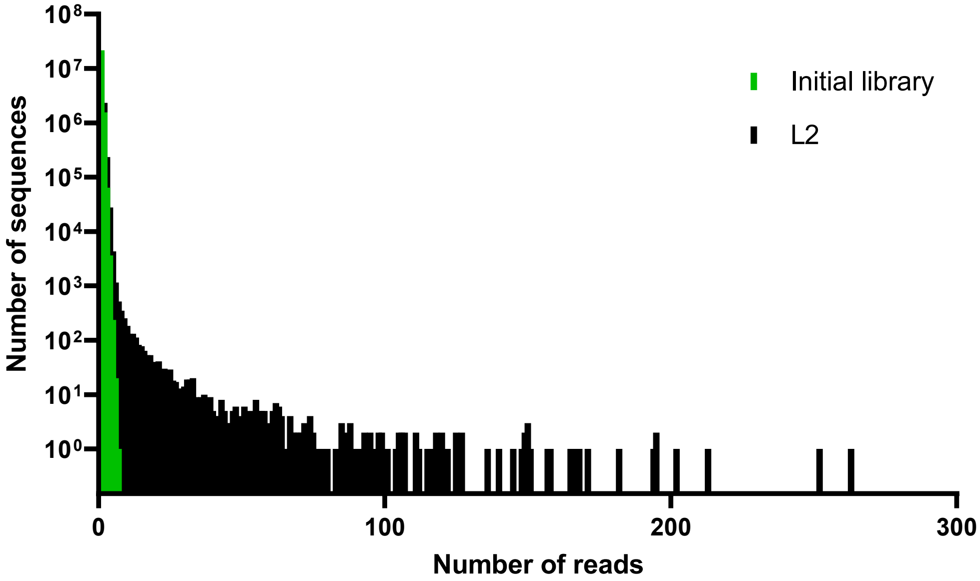

Supplementary Figure 11. Distributions of sequence abundances of L2 library after SRS and its initial library.

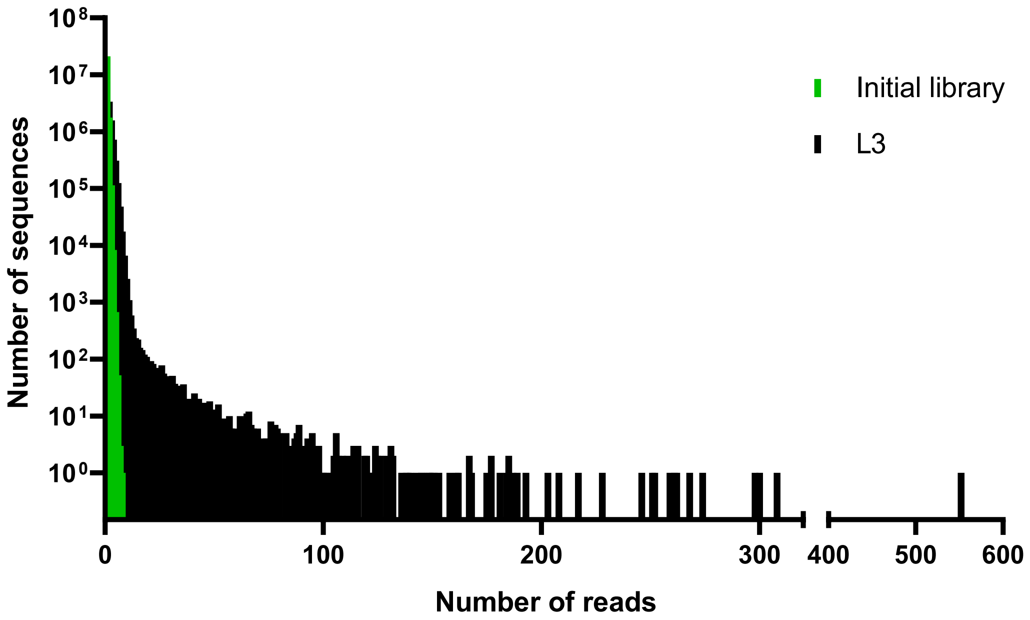

Supplementary Figure 12. Distributions of sequence abundances of L3 library after SRS and its initial library.

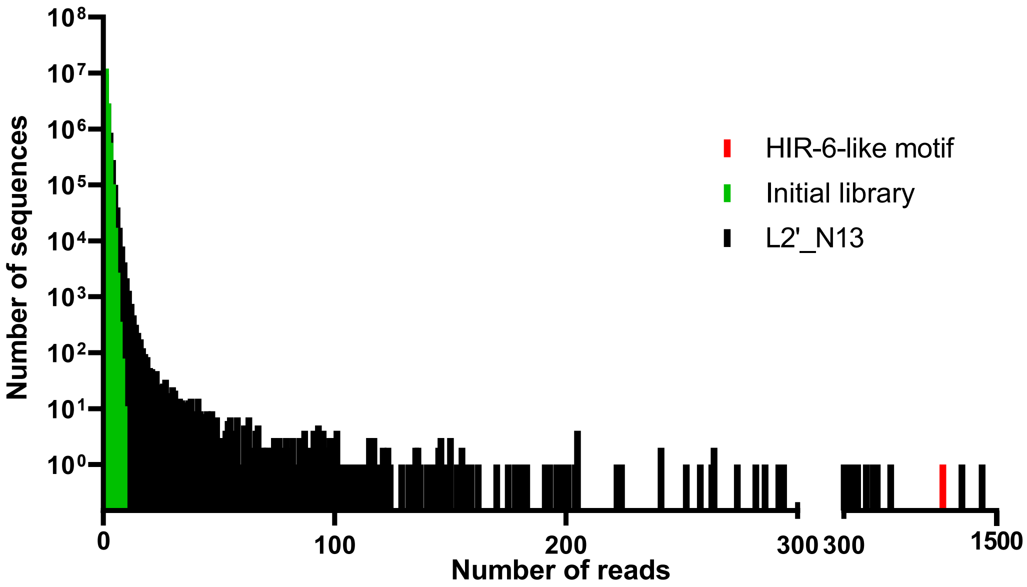

Supplementary Figure 13. Distributions of sequence abundances of L2'_N13 library after SRS and its initial library. Top-count sequence with HIR-6-like motif highlighted in red.

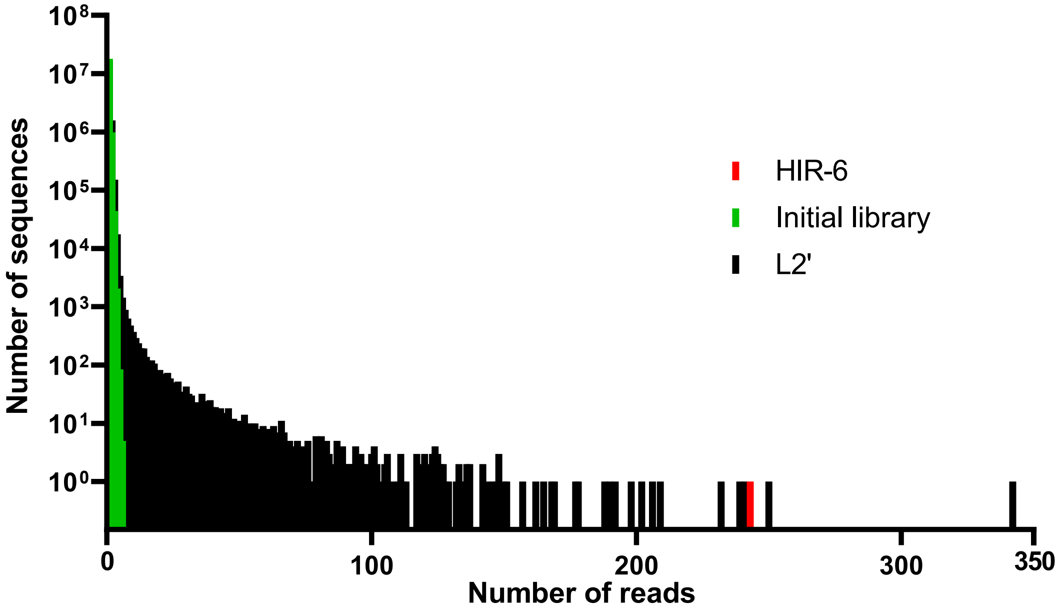

Supplementary Figure 14. Distributions of sequence abundances of L2' library after SRS and its initial library. Top-count sequence with HIR-6 highlighted in red.

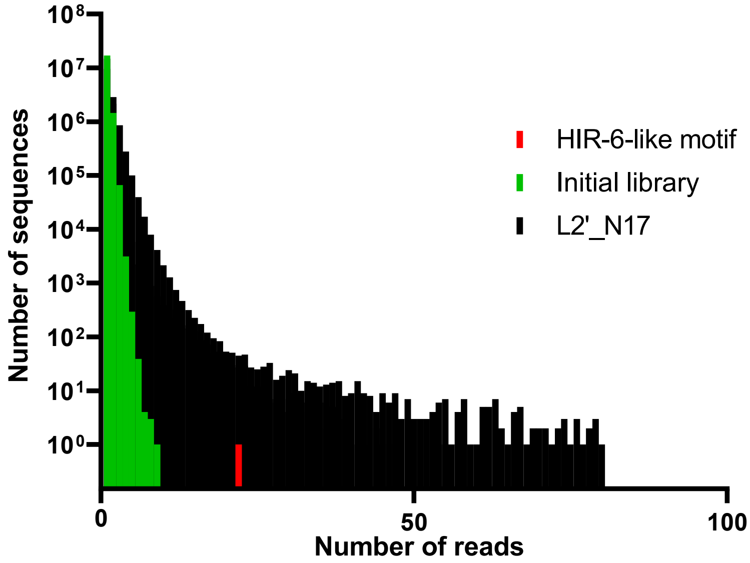

Supplementary Figure 15. Distributions of sequence abundances of L2'_N17 library after SRS and its initial library. Top-count sequence with HIR-6-like motif highlighted in red.

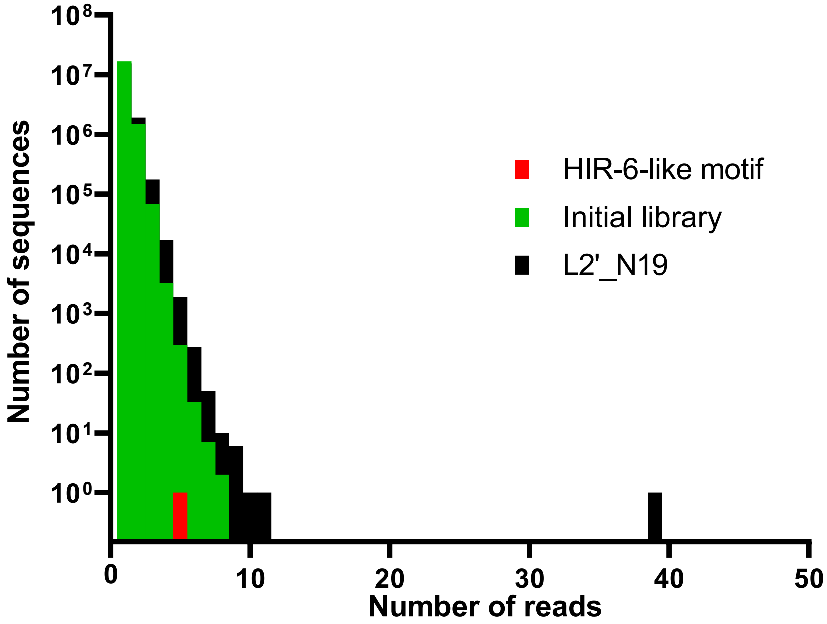

Supplementary Figure 16. Distributions of sequence abundances of L2'_N19 library after SRS and its initial library. Top-count sequence with HIR-6-like motif highlighted in red.

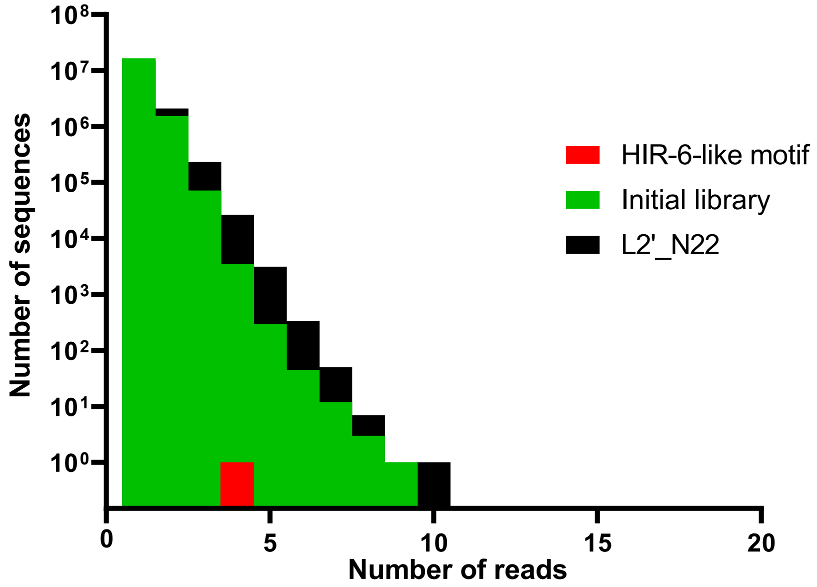

Supplementary Figure 17. Distributions of sequence abundances of L2'_N22 library after SRS and its initial library. Top-count sequence with HIR-6-like motif highlighted in red.

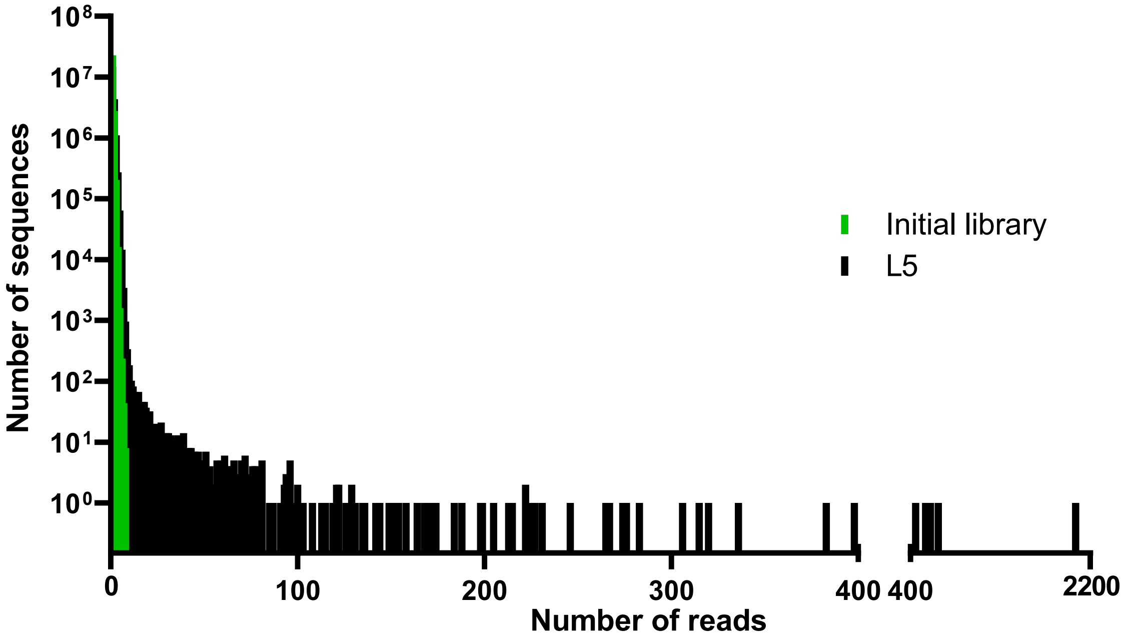

Supplementary Figure 18. Distributions of sequence abundances of L5 library after SRS and its initial library.

6.3 Cluster analysis

Clusters generated from SRSs are presented in Supplementary Tables 6-11. SRS using an unmodified L1 library, modified L2'_N19 and L2'_N22 did not produce any identifiable clusters due to the lack of enriched sequences.

Supplementary Table 6. Identified clusters from SRS using L2 library.

Cluster	Seed sequence	No. sequences	Total NGS reads
1	TCGACCTATCTCGAA	29	2752
2	TAGACCTTATGTCCC	6	778
3	CGACTAGATGTTACC	4	572
4	GTGACCTATCACCGG	3	383
5	CCTTACATCTAATGG	9	761
6	CCGATTATCCCTTGG	6	762
7	CAGACTTCGTTTGCA	15	1166
8	TAGACATCTACGGGC	22	1928
9	CCATGATCTTTGAGG	6	527
10	AATTCCCAACAGGCC	3	269

11	GCCGCTTTAACGTAA	9	802
12	GTTCAGCCTAACGGT	2	223
13	GAACACCACTGTTCG	2	218
14	CGCCTATACATCTAC	2	175
15	GATCAACTGGTCACG	2	181
16	CACCTTGAGGGTTAC	3	240
17	CATCATGATATGGGC	5	420
18	TATCTGATTTGTCCC	2	211
19	GAGACCCCATAGGTG	3	236
20	ATCTCTTTGCTCACG	3	204

Supplementary Table 7. Identified clusters from SRS using L3 library.

Cluster	Seed sequence	No. sequences	Total NGS reads
1	GCATCTACACTTCGC	65	7915
2	CCCTGATCAAGTCTG	13	1549
3	GCCCCCGATATTCGA	28	2496
4	CGATATTCGACCCGA	16	1387
5	AGGGTCCATTCTAGG	45	4153
6	CGAGCTGAATGGCCC	41	3489
7	GGAACTCCAATACCG	13	1261
8	GCCCATGTTAACCTC	34	2672
9	CCCCTACGTGATCTC	9	828
10	CGAACATGCTTGTCC	7	537
11	AACTCAAGTCGGGGC	27	2298
12	CCGGTGACCAACACG	8	760
13	TACGTGATCTCGACC	4	310
14	GAATTCCGTTTAGCC	1	116
15	TACGTGGTACCTGTC	2	158
16	GATCCATTAACCCCC	2	155
17	AGCTCGACATGCCGC	5	308
18	GCTGAATGGCACGGG	3	216
19	GATTGACGTACGGGC	5	309
20	ACGATTCTGTGGTGG	1	80

Supplementary Table 8. Identified clusters from SRS using L2' library.

Cluster	Seed sequence	No. sequences	Total NGS reads
1	GGTTTAACGTAACAC	45	5693
2	TTGTGTGTGAAGAGA	4	609
3	GTTAGACTCAAGGTG	302	31939
4	TTTTGTAGATCGAGC	70	5915
5	GTTATCATGATATGC	19	1412

6	GTGTCGCTTATGAAA	19	1416
7	GCATTGACGCAGAAA	2	337
8	TTGACCTATCAAGAT	4	401
9	ATGATGTCCTGTCTG	2	246
10	GGTATTTGTTTACGA	4	371
11	GCGCGGTAGATGTAC	4	333
12	ATCTTGACCTATCAA	6	488
13	TTTTGTGCAATACAT	5	427
14	TTGCGATTGTTACAA	19	1328
15	TTGCATTATACTCGA	7	529
16	TGCATATATCACACT	8	619
17	TTATGCATATCCGGT	11	821
18	TACCTTGATCTTAAG	1	111
19	CGCGATGAACTGGTC	1	98
20	GCATGATGTTTTTAC	11	791

Supplementary Table 9. Identified clusters from SRS using L2'_N13 library.

Cluster	Seed sequence	No. sequences	Total NGS reads
1	GTTAGCCTCAAGT	47	9347
2	GTTTAACGTAACA	11	2341
3	ATCATCATATCGG	44	6076
4	ATGCTTTCTTTTC	14	2420
5	CGTCGTTACCTAC	4	517
6	TTTTGTAGATCCA	16	2100
7	ATCCGTCCCTAAC	5	533
8	GCATTATCCCTTG	7	981
9	TGCGATTGTTACA	16	1664
10	TTGTGATGAGGGC	3	319
11	AAACACATTTGAC	4	418
12	TTGACCTATCAAA	22	2093
13	CGTGATATCACAT	6	742
14	ACATCCGATCCTA	5	448
15	GCTGATCAATATA	6	567
16	ATGACATCGTAAT	4	372
17	GTTCTATCCAGTC	2	195
18	TGGTTCGCATGTT	3	280
19	TTCGCTTCACTTT	1	109
20	ATTACCAATTCAT	1	106

Supplementary Table 10. Identified clusters from SRS using L2'_N17 library.

Cluster	Seeds sequence	No. sequences	Total NGS reads
1	CATCCTTGACCTATCAA	43	1382
2	ACGGTTTAACGTAACAC	33	1038
3	ATGGTGACCTATCACAC	4	121
4	GCTTTAACGTAAGACGG	4	117
5	ATGATGTCCTGTCTCTT	1	31
6	GTTAGCCTCAAACGAGG	2	56
7	TGCCGATTATCCCTTGG	3	85
8	GAGCCTTTAACGTAAGA	1	26
9	GTTTTTGACCTATCAAC	1	25

Supplementary Table 11. Identified clusters from SRS using L5 library.

Cluster	Seed sequence	No. sequences	Total NGS reads
1	TGTGATGCCTGACAG	29	7173
2	CACATGATACGATAT	53	7954
3	CATTGTGACCTATCA	3	670
4	GCAAGCAATGTTCCG	1	306
5	GATGCGATACGTTCG	2	317
6	GCTGTATTGACCTAT	3	369
7	GCGAGGCATAACGGA	3	350
8	GCAAGTGAGCAAGTG	1	198
9	GGGACCCATAGCCGG	1	174
10	GTCAATAACACGCAG	1	173
11	TTCTCTGCTTGTTCG	1	168
12	CTGAAAGCCTACGCA	1	149
13	GATGCGCTTAGAGAT	4	371
14	CACTTCCTAGAAGGG	2	197
15	TTCTTTGATAACAAA	1	131
16	CCTTGAGCTCGGGGC	4	341
17	GCCTTTGATCACTCG	1	129
18	CCTAGGGCCTGCTCA	1	126
19	GCCGATGCTCTAGCG	1	121
20	CCTGAAGGGCCCCCG	1	119

6.4 Sequence alignment of most active clusters for L2 and L2' selections

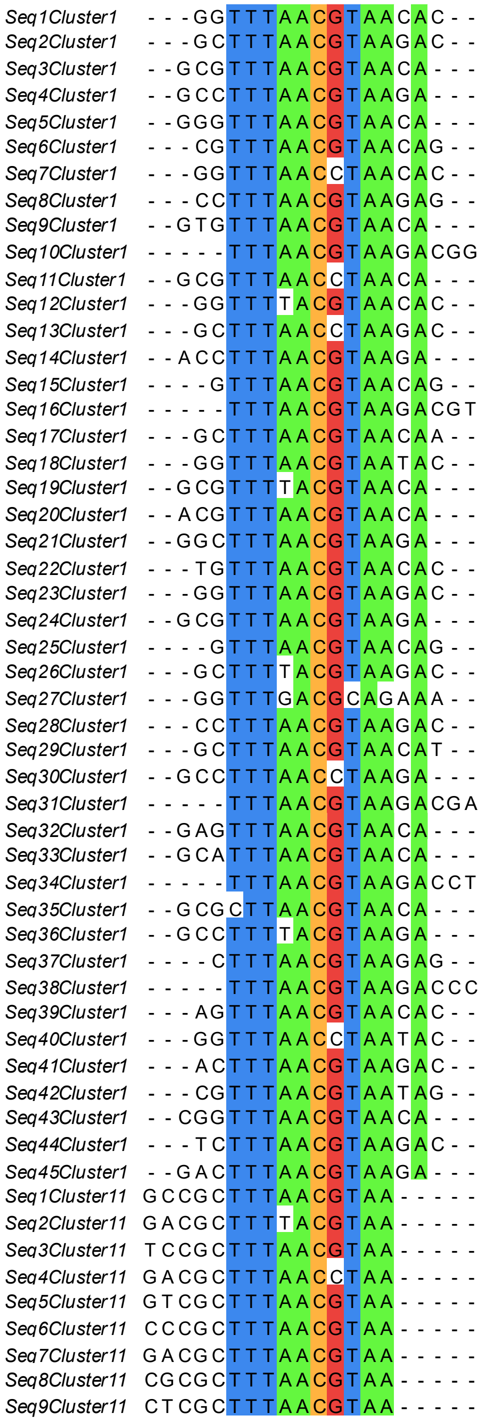

Supplementary Figure 19. Sequence alignment of randomized regions from sequences found in cluster 1 (from L2' library) and cluster 11 (from L2 library) performed with MUSCLE[3] by MEGA12[4] and visualized with Jalview2.11.5.0[5]. Sequences with an identity of over 80% are highlighted.

6.5 Covariation analysis

Pre-processed variable regions were filtered for the correct length of 55 nucleotides, and unique sequences were counted with basic bash commands. This dataset, containing aligned sequences and their counts, was then used to calculate mutual information between all pairs of positions with a custom Python script according to the equation, and the values of mutual information were arranged into a symmetric matrix (Supplementary Equation 1).

$$\mathrm{I}(X;Y) = \sum_{x\in X}\sum_{y\in Y} P(x,y)\log\frac{P(x,y)}{P(x)P(y)}$$

Supplementary Equation 1. The mutual information between a pair of positions X and Y, denoted as I(X; Y), is calculated as a double sum (one over all possible values at position X and the second over all possible values at position Y) of the products of the frequencies of a given dinucleotides and the logs of ratios of frequencies of given dinucleotides (denoted as P(X,Y)(x, y)) and the products of individual frequencies of corresponding single nucleotides (denoted as PX(x) and PY(y) respectively).

7. Enzymatic synthesis of modified oligonucleotides

Aptamer candidates selected for affinity screening and subsequent truncated, mutated and scrambled sequences of HIR-6 and HIR-8 aptamers were prepared by PEX using a 5'-biotinylated template, 5'-Cy5-labeled reverse primer (Supplementary Table 1), and strand separated as described in the method sections in the manuscript. If needed, more PEX reactions were performed, merged and HPLC purified. PEX conditions were applied with some variations (Supplementary Table 12).

Supplementary Table 12. PEX reaction conditions.

Name	dNTPs / $dN^{R}TPs$	KOD XL DNA polymerase (U)	Extension time (h)
HIR-6_V1/3/4	0.5 mM	3.75	3
Rest of the sequences	0.5 mM	1.25	2

7.1 Generation of single-stranded DNA using magnetic beads

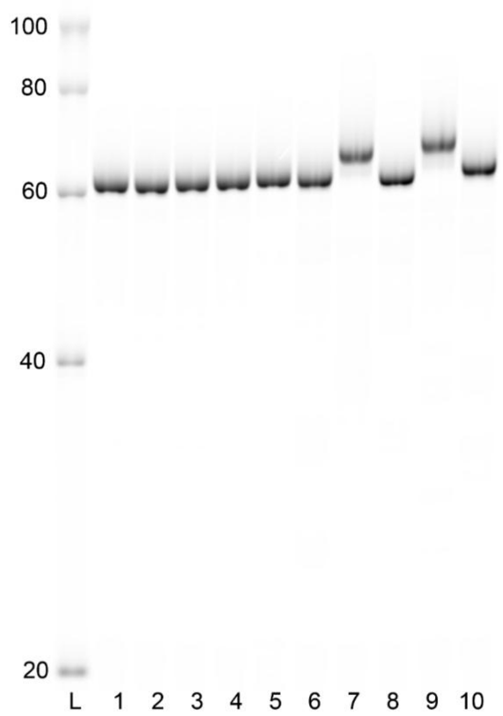

Supplementary Figure 20. Cy5 scan of denaturing PAGE analysis of ten aptamer candidates from SRSs using L2 and L3 libraries. Cy5-labeled ssDNA ladder (lane L); five candidates from L2 (lanes 1-5); five candidates from L3 (lanes 6-10).

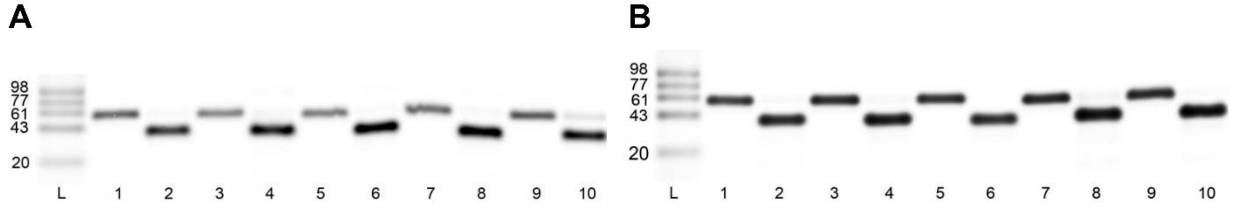

Supplementary Figure 21. Cy5 scan of native agarose gel of ten aptamer candidates from SRSs using L2 and L3 libraries. Cy5-labeled ssDNA ladder (lanes L); A) five candidates from L2, dsDNA from PEX (odd lanes); modified single strands after magnetoseparation (even lanes). B) five candidates from L3, dsDNA from PEX (odd lanes); modified single strands after magnetoseparation (even lanes).

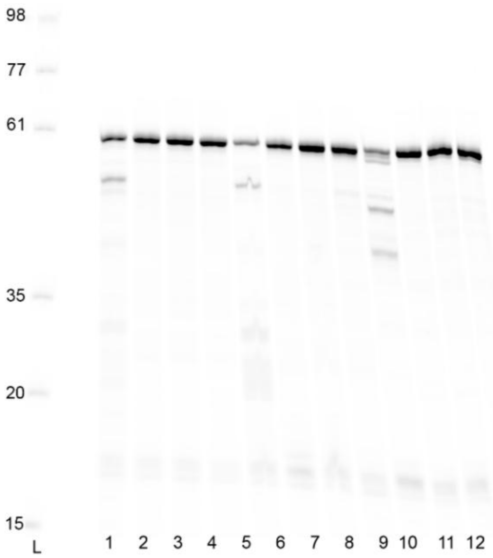

Supplementary Figure 22. Cy5 scan of denaturing PAGE analysis of PEX of aptamer candidates from SRS using L2' library. Different concentrations of dN^{R}TPs were tested for PEX optimizations. Cy5-labeled ssDNA ladder (lane L); HIR-1 (lanes 1-4); HIR-2 (lanes 5-8); HIR-3 (lanes 9-12); 0.2 mM dN^{R}TPs (lanes 1, 5 and 9), 0.4 mM dN^{R}TPs (lanes 2, 6 and 10); 0.5 mM dN^{R}TPs (lanes 3,7 and 11); 0.6 mM dN^{R}TPs (lanes 4, 8 and 12).

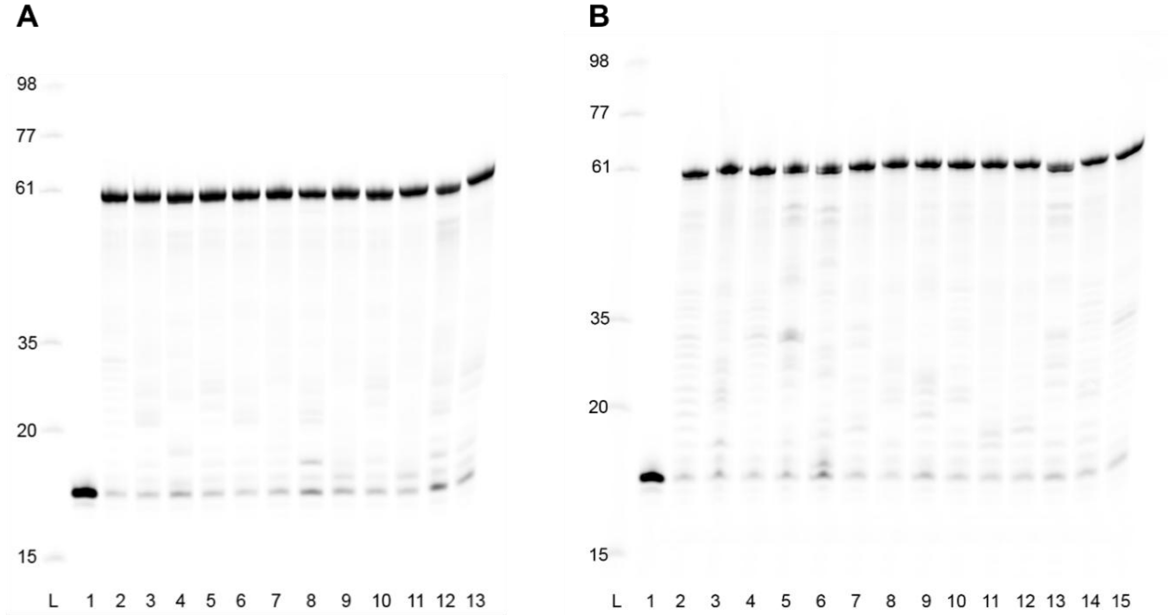

Supplementary Figure 23. Cy5 scan of denaturing PAGE analysis of PEX of aptamer candidates from SRS using L2' library. Cy5-labeled ssDNA ladder (lanes L); Rev2 primer used in PEX (lanes 1); A) HIR-4 to HIR-15 (lanes 2-13). B) HIR-16 to HIR-29 (lanes 2-14).

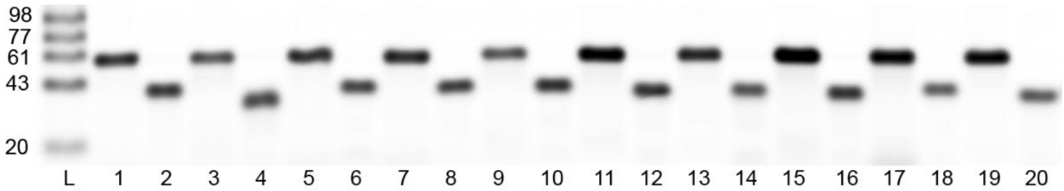

Supplementary Figure 24. Cy5 scan of native agarose gel of aptamer candidates from SRS using L2' library. Cy5-labeled ssDNA ladder (lane L); HIR-1 to HIR-10 as dsDNA after PEX (odd lanes); HIR-1 to HIR-10 as ssDNA after magnetoseparation (even lanes).

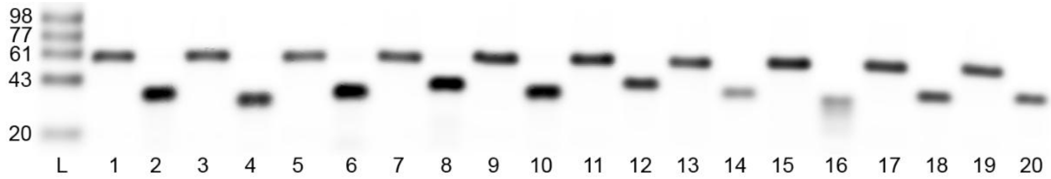

Supplementary Figure 25. Cy5 scan of native agarose gel of aptamer candidates from SRS using L2' library. Cy5-labeled ssDNA ladder (lane L); HIR-11 to HIR-20 as dsDNA after PEX (odd lanes); HIR-11 to HIR-20 as ssDNA after magnetoseparation (even lanes).

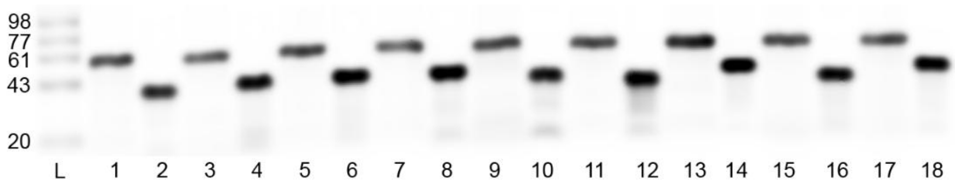

Supplementary Figure 26. Cy5 scan of native agarose gel of aptamer candidates from SRS using L2' library. Cy5-labeled ssDNA ladder (lane L); HIR-21 to HIR-29 as dsDNA after PEX (odd lanes); HIR-21 to HIR-29 as ssDNA after magnetoseparation (even lanes).

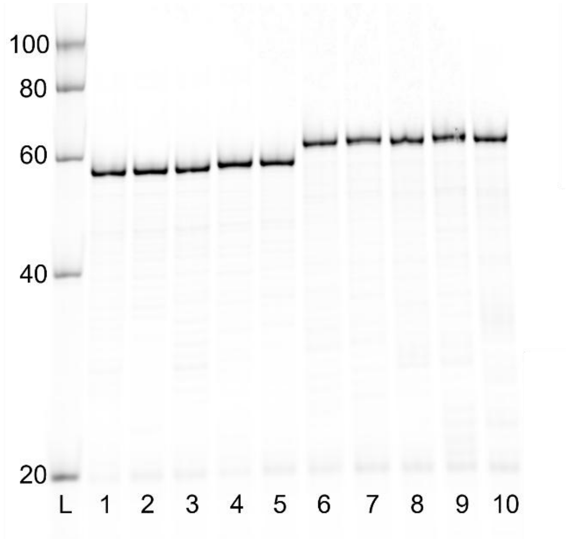

Supplementary Figure 27. Cy5 scan of dPAGE analysis of aptamer candidates from SRSs using L2'_N13 and L2'_N17 libraries. Cy5-labeled ssDNA ladder (lane L); five candidates from L2'_N13 (lanes 1-5); five candidates from L2'_N17 (lanes 6-10).

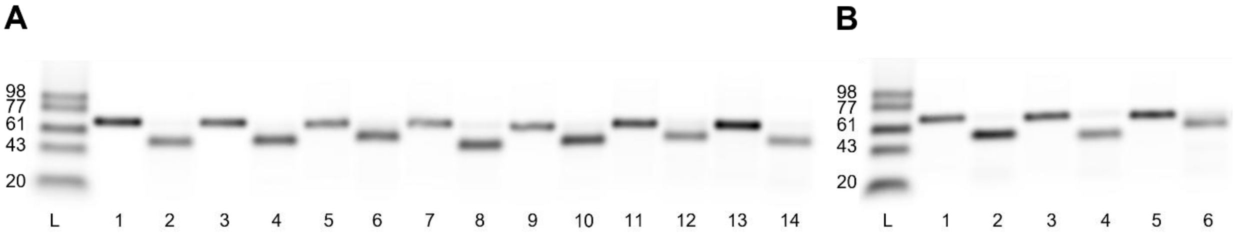

Supplementary Figure 28. A) and B) Cy5 scan of native agarose gel of aptamer candidates from SRSs using L2'_N13 and L2'_N17 libraries. Cy5-labeled ssDNA ladder (lanes L); A) five candidates from L2'_N13 and two candidates from L2'_N17, dsDNA after PEX (odd lanes), modified single strands after magnetoseparation (even lanes). B) three candidates from L2'_N17, dsDNA after PEX (odd lanes), modified single strands after magnetoseparation (even lanes).

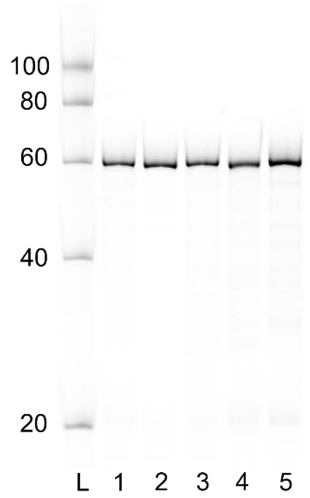

Supplementary Figure 29. Cy5 scan of denaturing PAGE analysis of five aptamer candidates from SRSs using L5 library. Cy5-labeled ssDNA ladder (lane L); five candidates from L5 (lanes 1-5).

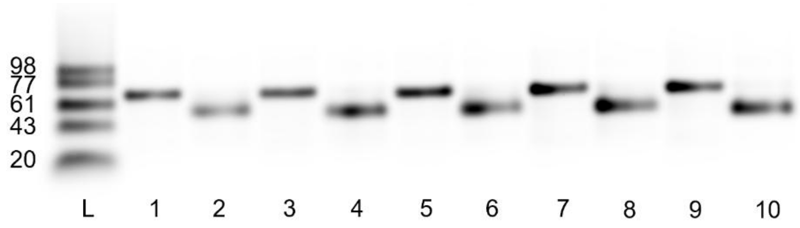

Supplementary Figure 30. Cy5 scan of native agarose gel of aptamer candidates from SRS using L5 library. Cy5-labeled ssDNA ladder (lane L); dsDNA after PEX (odd lanes); DIR-1 to DIR-5 as ssDNA after magnetoseparation (even lanes).

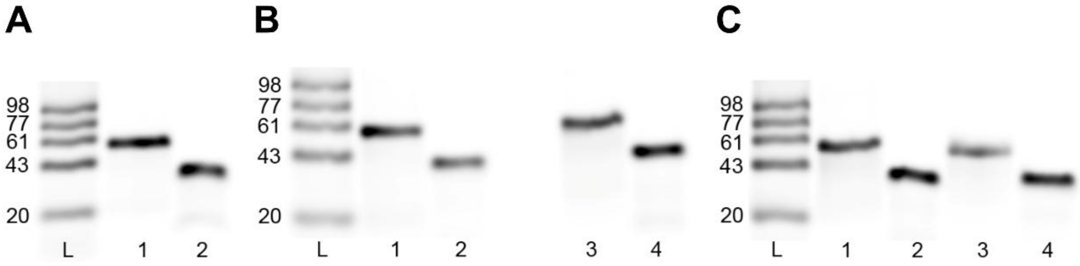

Supplementary Figure 31. Cy5 scan of native agarose gel of HIR-6 aptamer and its variant sequences. Cy5-labeled ssDNA ladder (lanes L); dsDNA after PEX (odd lanes); ssDNA after magnetoseparation (even lanes). A) HIR-6_V2 (lanes 1-2). B) HIR-6 (lanes 1-2); HIR-6_V3 (lanes 3-4). C) HIR-6_V1 (lanes 1-2); HIR-6_V4 (lanes 3-4).

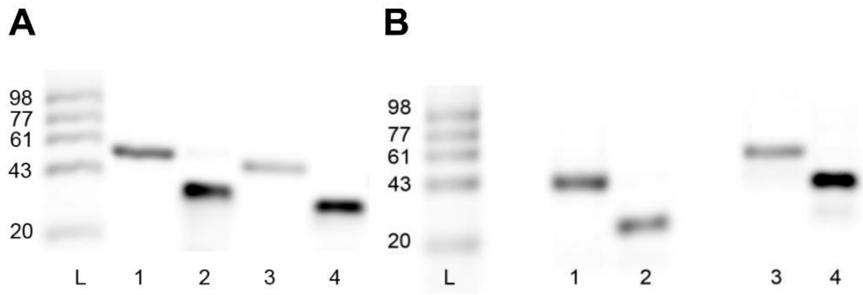

Supplementary Figure 32. Cy5 scan of native agarose gel of truncated and mutated sequences of HIR-6 aptamer. Cy5-labeled ssDNA ladder (lanes L); dsDNA after PEX (odd lanes); ssDNA after magnetoseparation (even lanes). A) HIR-6_T1 (lanes 1-2); HIR-6_T2 (lanes 3-4). B) HIR-6_T3 (lanes 1-2); HIR-6_M2 (lanes 3-4).

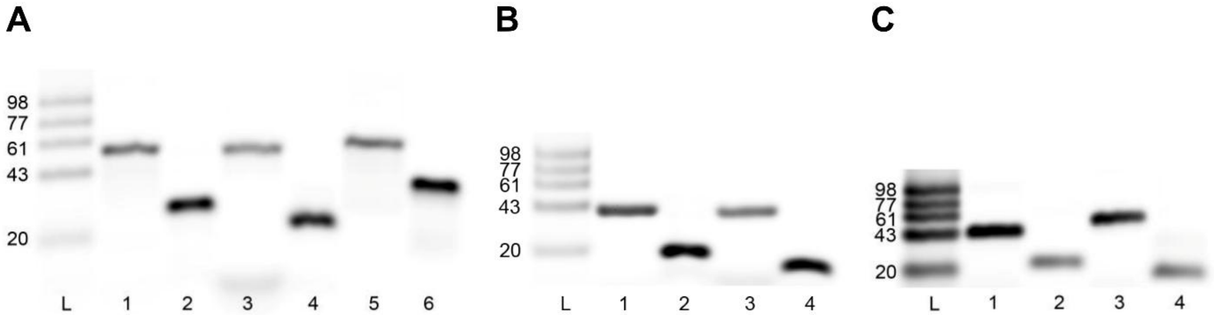

Supplementary Figure 33. Cy5 scan of native agarose gel of truncated sequences of HIR-6 aptamer. Cy5-labeled ssDNA ladder (lanes L); dsDNA after PEX (odd lanes); ssDNA after magnetoseparation (even lanes). A) HIR-6_T4 (lanes 1-2); HIR-6_T5 (lanes 3-4); HIR-6_M1 (lanes 5-6). B) HIR-6_T7 (lanes 1-2); HIR-6_T8 (lanes 3-4). C) HIR-6_T9 (lanes 1-2); HIR-6_T6 (lanes 3-4).

8. Binding affinity and specificity assays

Because two identical HIR monomers form a 2-fold symmetric homo-dimeric structure, each monomer can bind one aptamer molecule. Consequently, we calculated the dissociation constants (K_Ds) using a 1:1 HIR:HIR-6 ratio using three different techniques.

8.1 Fluorescent Ni-plate binding assay (FNBA)

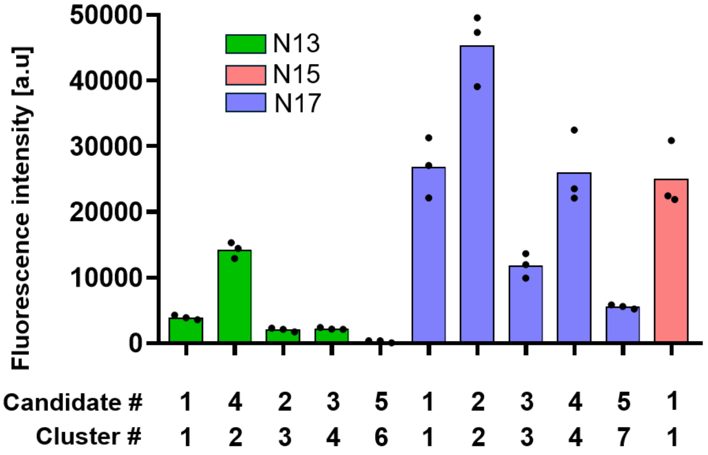

Supplementary Figure 34. FNBA assay of top clustered sequences from L2'_N13, L2'_N17 and L2'; n=1 performed as technical triplicate.

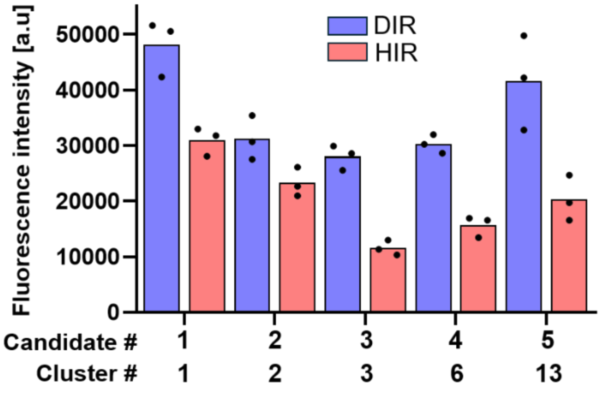

Supplementary Figure 35. FNBA assay of top clustered sequences from L5; n=1 performed as technical triplicate.

8.2 Microscale thermophoresis (MST)

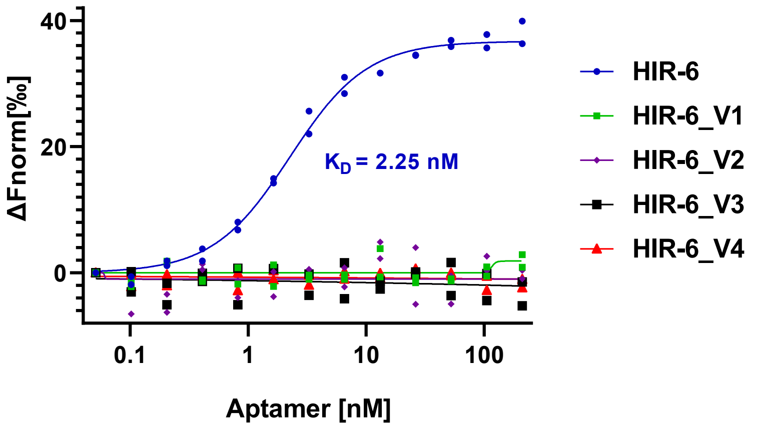

Supplementary Figure 36. MST measurements of HIR-6 and its variants with calculated K_D value from n=2 independent experiments.

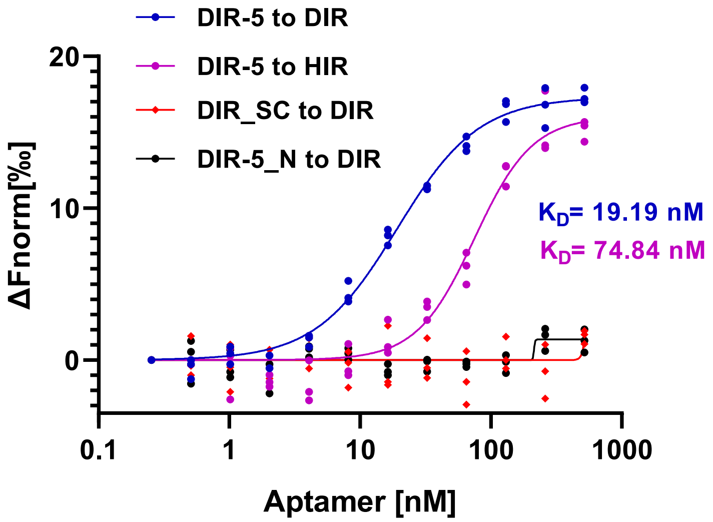

Supplementary Figure 37. MST measurements of DIR-5 and its control scrambled (DIR_SC) and non-modified (DIR-5_N) sequences with calculated K_D values for DIR and HIR; n=1 measured as technical triplicate.

9. Cell assays

9.1 Receptor-binding studies

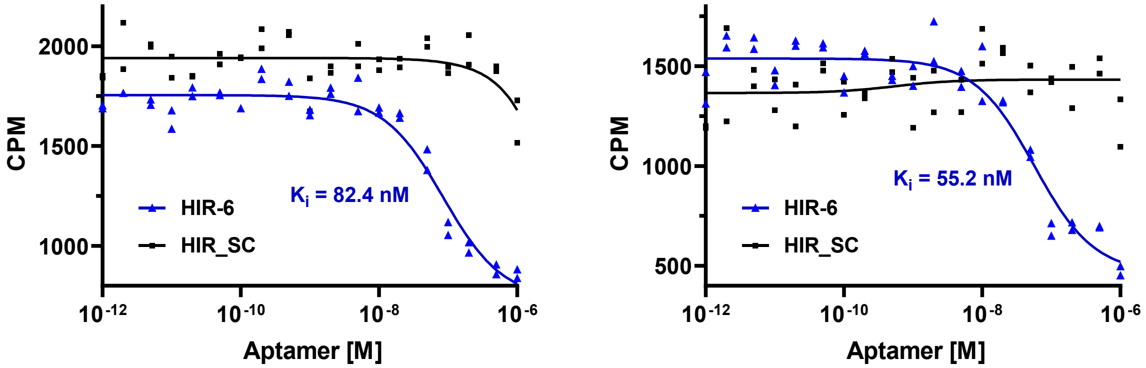

Supplementary Figure 38. HIR-6 binding competition with [^{125}I]monoiodotyrosyl-A14-insulin for IR-A isoform in human IM-9 lymphocyte cell membranes using increasing concentrations of HIR-6 and HIR_SC. A decrease in radioactively labeled insulin bound to HIR was measured (CPM). Each graph represents n=1 independent experiment performed as technical duplicate.

9.2 Receptor phosphorylation and antagonism assay

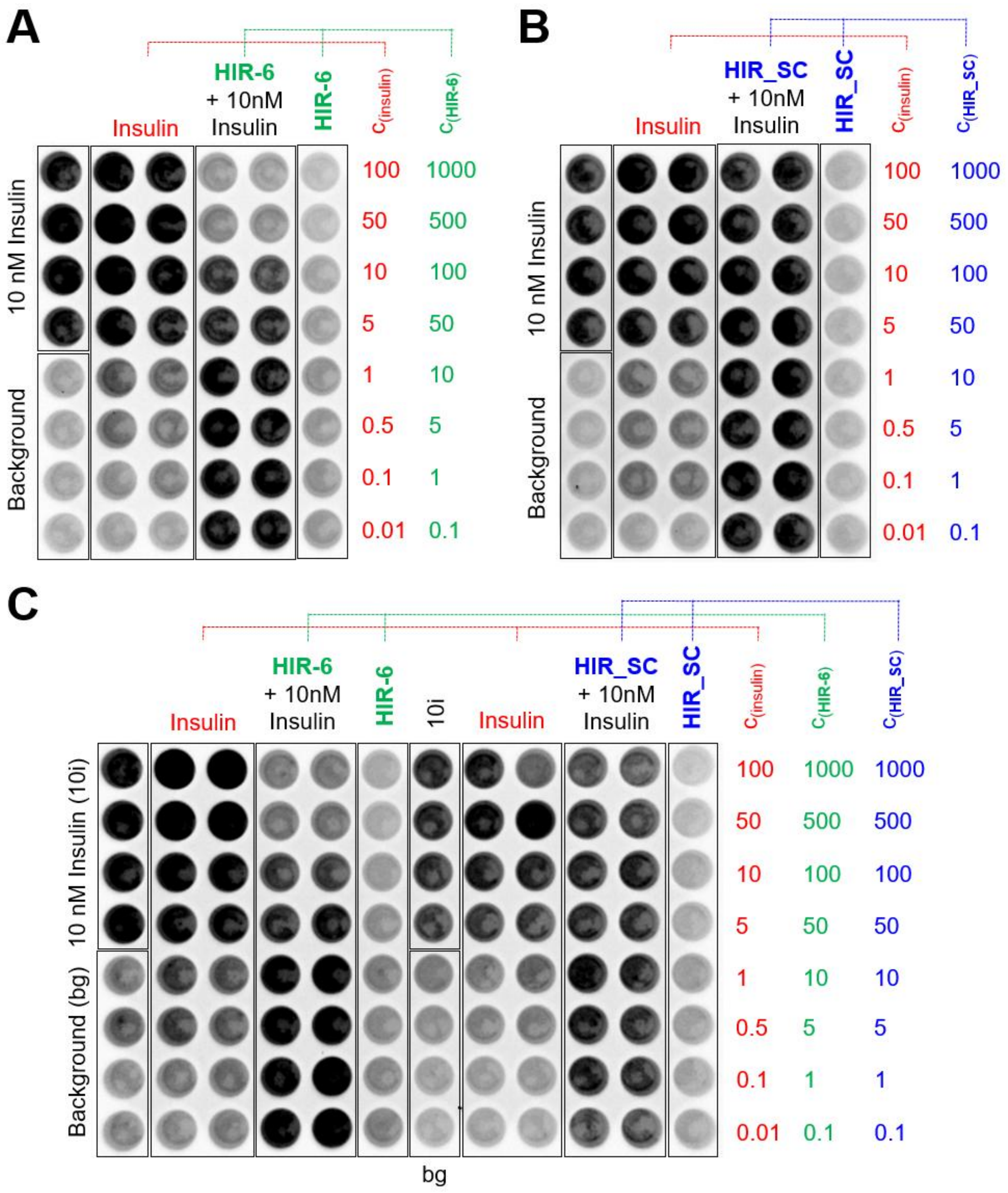

Supplementary Figure 39. Stimulation of HIR phosphorylation with HIR-6. Individual plates from In-Cell Western Assay used for Fig. 3B and Supplementary Fig. 41. The mouse fibroblasts transfected with human IR-A were treated with insulin, HIR-6 and HIR_SC at specified concentrations for 20 min. Formaldehyde fixed permeabilized cells were incubated with anti-phospho-IGF-1 Rβ (Tyr1135/1136)/IRβ (Tyr1150/1151) and developed with peroxidase-labeled anti-rabbit secondary antibody (Sigma). SuperSignal West Femto maximum sensitivity substrate was added to each well, and chemiluminescence was detected using the ChemiDoc MP Imaging System. Data were subtracted from the background values and expressed as the contribution of phosphorylation relative to the 10 nM insulin signal.

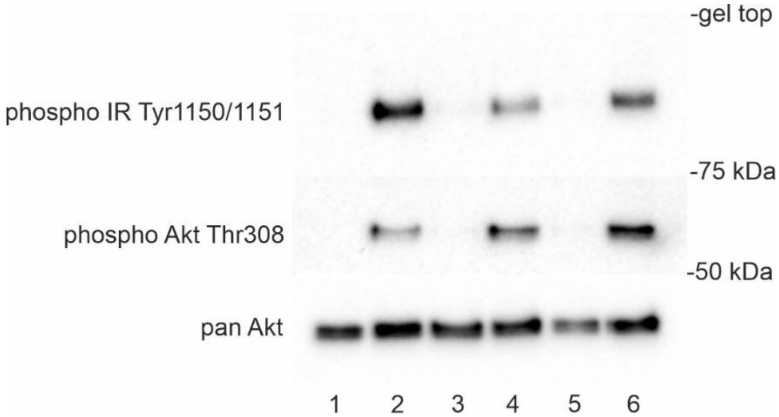

Supplementary Figure 40. Control western blots (n=1) of experiments in Supplementary Fig. 39 showing stimulation of IR-A and Akt in mouse fibroblasts derived from IGF-1 R knockout mice stably transfected with human IR-A. Cells were stimulated with 10 nM insulin (lane 2), 500 and 100 nM HIR-6 (lanes 3 and 5, respectively). The aptamers were co-stimulated with 10 nM insulin (lane 4: 500 nM HIR-6 and lane 6: 100 nM HIR-6). Control unstimulated cells are in lane 1. Membranes were cut at 75 kDa and 50 kDa standards, and respective parts were developed with anti-phospho-IGF-1 Rβ (Tyr1135/1136)/IRβ (Tyr1150/1151) (Mw above 75 kDa) and anti phospho-Akt (Thr308) (C31E5E) (Mw between 75 and 50 kDa). The same samples were stained in parallel with Akt (pan) antibody used as a loading control.

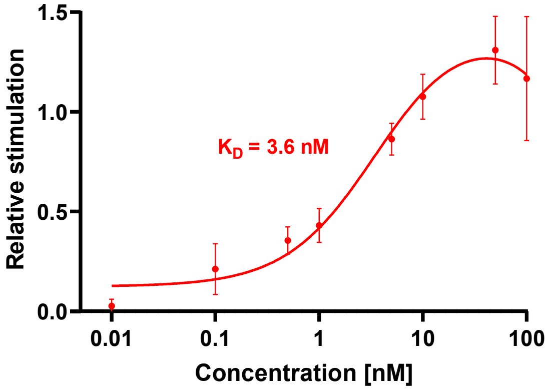

Supplementary Figure 41. Mouse fibroblast cells expressing HIR-A stimulated with increasing concentrations of insulin. K_D value was calculated from n=3, each performed as a technical duplicate. Error bars represent the mean ± s.d.

10. Cryo-electron microscopy (cryo-EM) of HIR-HIR-6 complex

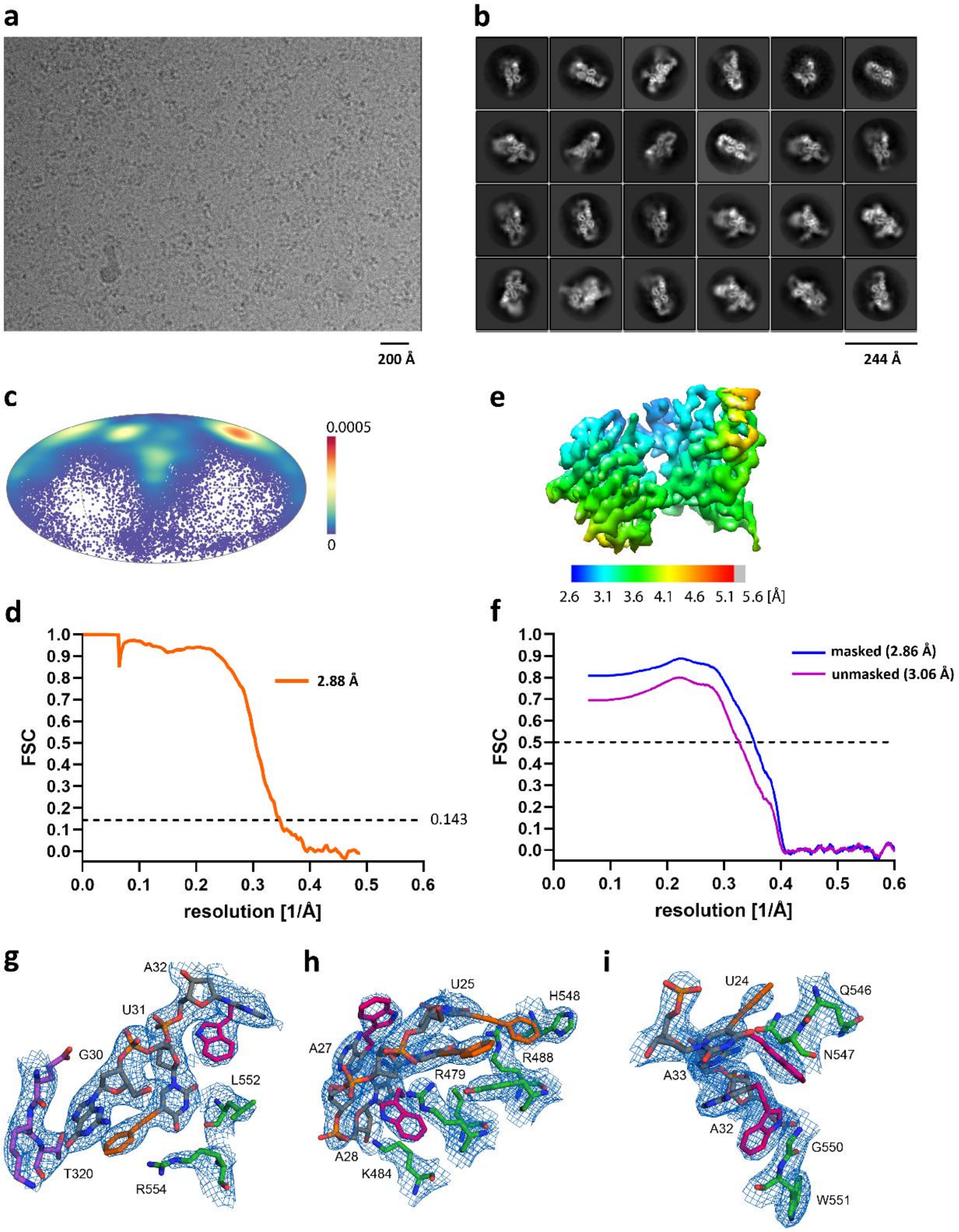

Supplementary Figure 42. Cryo-EM of HIR-HIR-6 complex. a) Micrograph of the complexes in free-standing ice after MotionCor2[6] correction at defocus of ~2.5 µm. b) 2D-class averages of HIR-HIR-6 complexes. c) Angular distribution for particles of a on globe-like plane. d) Fourier shell correlation (FSC) curves for the HIR-HIR-6 complex. The plot of the FSC between two

independently refined half-maps shows the overall resolution of the two maps as indicated by the gold standard FSC 0.143 cut-off criterion.[7] e) Surface representation of local resolution distribution focused on the FnIII-1, L2' and HIR-6 part of the HIR-HIR-6 complex. The map is colored according to the local resolution calculated within the RELION software package. Resolution is as indicated in the color bar. f) Fourier Shell Correlation (FSC) between the HIR-HIR-6 complex model (masked and unmasked) and the EM density map. FSC 0.5 cut-off criterion is indicated. g, h, i) Examples of the LocScale cryo-EM maps (isomesh level 60) contoured around sections of the views in Fig. 6a,b,c, respectively, visualizing modified DNA bases of the HIR-6 aptamer together with interacting HIR residues.

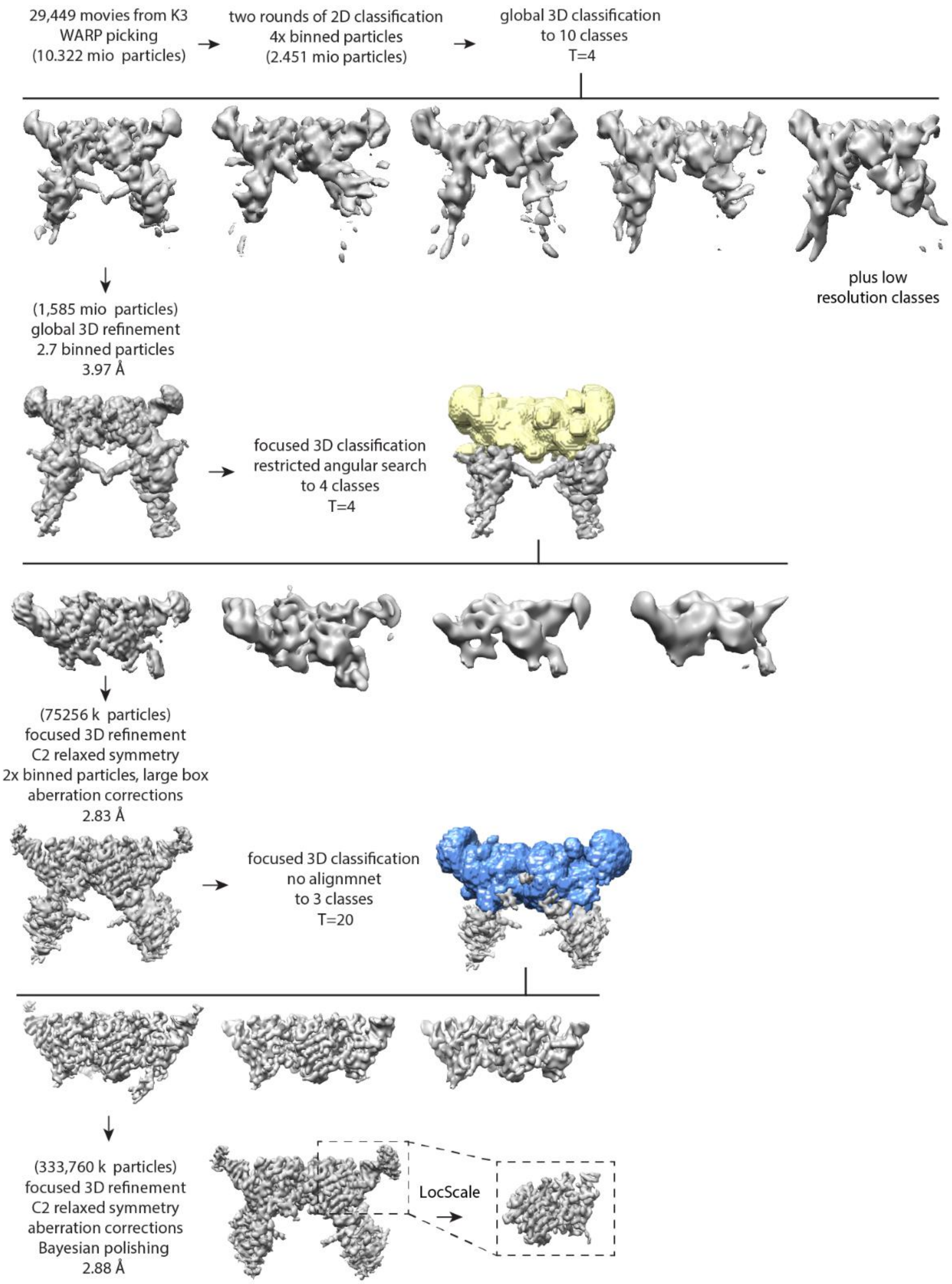

Supplementary Figure 43: Cryo-EM data processing workflow for HIR-HIR-6 complex. Cryo-EM single particle analysis data processing workflow of the HIR-HIR-6 complex. The workflow shows a summary of the 3D classification and refinement scheme, together with representative focused classification masks.

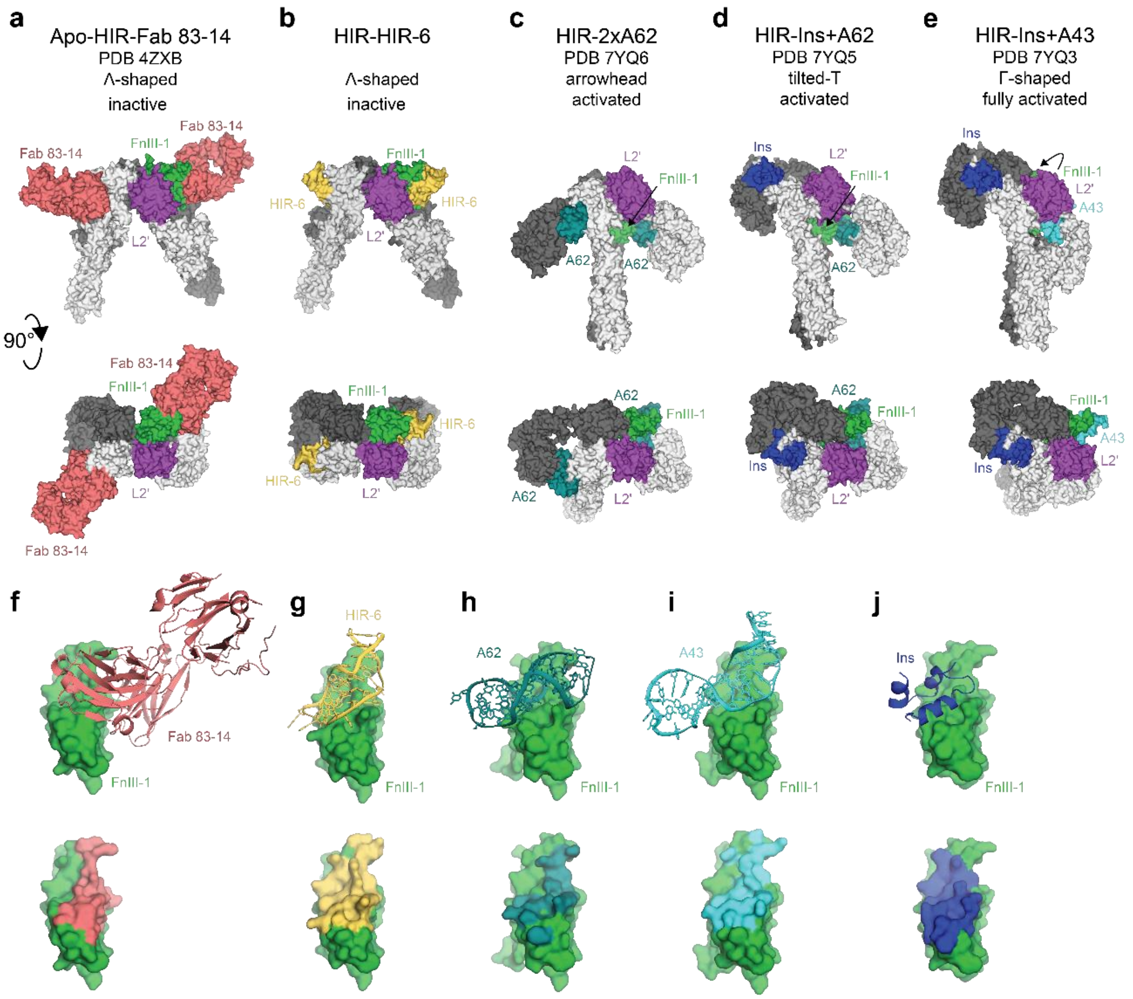

Supplementary Figure 44: Structural comparison of HIR-binding ligands. **a-e)** Surface representation (two perpendicular views) of the HIR complexes with antibody Fab 83-14[8]. and aptamers HIR-6, A62, A62 + insulin, and A43 + insulin[9], respectively. Protomers of the HIR dimer are colored in light and dark grey, the FnIII-1 and L2 domains from opposite protomers are green and purple, respectively. The HIR complexes were aligned on the L2' domain. Fab 83-14 is salmon, HIR-6 is yellow, A62 is teal, A43 is cyan, insulin molecule is blue. The individual domains of HIR in the HIR-HIR-6 complex were rigid body fitted into the low-resolution cryo-EM reconstruction of the HIR ectodomain (Fig. 5a). **f-j)** Comparison of the binding of antibody Fab 83-14, aptamers HIR-6, A62, A43, and insulin (PDB 6PXV)[10], respectively, to FnIII-1 domain together with individual binding interfaces. While stabilizing different HIR conformations, all aptamers and Fab 83-14 bind to overlapping interfaces in FnIII-1, which also overlap with the insulin-binding site 2. However, the ultimate effect on the overall HIR conformation and activation state depends also on other contacts of the aptamers with other HIR domains and their mutual orientation.[11]

Supplementary Table 13: Cryo-EM structure determination and validation statistics.

**Name of structure**	HIR-HIR6 complex
PDB ID	9SA8
EMDB ID	EMD-54689
**Data collection and processing**	
Microscope	Titan Krios
Voltage (kV)	300
Camera	Gatan K3 BioQuantum
Magnification (x)	165,000
Nominal defocus range (negative μm)	0.5-3.0
Exposure time (s)	2.0
Electron exposure (e^-/Å^2)	51
Number of frames collected (no.)	40
Number of frames processed (no.)	25
Pixel size (Å)	0.5113
Micrographs (no.)	29,449
Total particle images (no.)	10,322,244
**Refinement**	
Particles per class (no.)	333,760
Map resolution (Å), 0.143 FSC	2.88
Map sharpening B factor (Å^2)	-19.9
Map versus model cross-correlation	0.89
Map versus model cross-correlation for ligands	0.90
**Model composition**	
Non-hydrogen atoms	2,704
Protein residues	274
Nucleotide residues	10
Chains	3
Ligands	9
**B factors (Å^2)** *min/max/mean*	
Protein	26.33/129.54/59.18
Nucleotide	42.28/168.77/106.46
Ligand	33.81/106.82/57.50
**R.m.s. deviations**	
Bond lengths (Å)	0.005
Bond angles (°)	1.029
**Validation**	
MolProbity score	1.92
EMRigner score	4.46
All-atom clashscore	5.96
Rotamer outliers (%)	3.27
**Ramachandran plot**	
Favored (%)	96.62
Allowed (%)	3.38
Outliers (%)	0

10.1 HIR-6 interactions

Supplementary Table 14. HIR-6 aptamer intra-structure interactions.

<table>
<tr><th colspan="5">Canonical base pairing</th></tr>
<tr><td>C20</td><td>base</td><td>G38</td><td>base</td><td rowspan="6">Watson-Crick base-pair hydrogen bonds</td></tr>
<tr><td>G21</td><td>base</td><td>C37</td><td>base</td></tr>
<tr><td>C22</td><td>base</td><td>G36</td><td>base</td></tr>
<tr><td>G23</td><td>base</td><td>C34</td><td>base</td></tr>
<tr><td>A33</td><td>base</td><td>U25</td><td>base</td></tr>
<tr><td>A32</td><td>base</td><td>U26</td><td>base</td></tr>
<tr><th colspan="5">Canonical base π-π stacking</th></tr>
<tr><td>C20</td><td>base</td><td>G21</td><td>base</td><td rowspan="10">π-π stacking</td></tr>
<tr><td>G21</td><td>base</td><td>C22</td><td>base</td></tr>
<tr><td>C22</td><td>base</td><td>G23</td><td>base</td></tr>
<tr><td>G23</td><td>base</td><td>G36</td><td>base</td></tr>
<tr><td>U25</td><td>base</td><td>U26</td><td>base</td></tr>
<tr><td>A27</td><td>base</td><td>A28</td><td>base</td></tr>
<tr><td>A28</td><td>base</td><td>C29</td><td>base</td></tr>
<tr><td>A32</td><td>base</td><td>A33</td><td>base</td></tr>
<tr><td>G36</td><td>base</td><td>C37</td><td>base</td></tr>
<tr><td>C37</td><td>base</td><td>G38</td><td>base</td></tr>
<tr><th colspan="5">Non-canonical hydrogen bonds</th></tr>
<tr><td>A27</td><td>base</td><td>A35</td><td>base</td><td rowspan="4">Hydrogen bond</td></tr>
<tr><td>A33</td><td>base</td><td>A35</td><td>base</td></tr>
<tr><td>U25</td><td>base</td><td>A27</td><td>indole</td></tr>
<tr><td>C29</td><td>base</td><td>A32</td><td>deoxyribose O4′</td></tr>
<tr><th colspan="5">Other π interactions</th></tr>
<tr><td>G23</td><td>base</td><td>A35</td><td>indole</td><td rowspan="4">π-π stacking</td></tr>
<tr><td>U25</td><td>base</td><td>A35</td><td>indole</td></tr>
<tr><td>A33</td><td>base</td><td>A35</td><td>indole</td></tr>
<tr><td>G30</td><td>base</td><td>U31</td><td>phenyl</td></tr>
<tr><td>A32</td><td>base</td><td>A33</td><td>indole</td><td>T-shape π-π stacking</td></tr>
<tr><td>C29</td><td>base</td><td>U31</td><td>deoxyribose O4′</td><td rowspan="3">Lone electron pair - π interaction</td></tr>
<tr><td>C34</td><td>deoxyribose O4′</td><td>A35</td><td>base</td></tr>
<tr><td>U26</td><td>deoxyribose O4′</td><td>A27</td><td>indole</td></tr>
<tr><td>U26</td><td>deoxyribose C5′</td><td>A27</td><td>indole</td><td rowspan="2">CH - π interaction</td></tr>
<tr><td>U25</td><td>deoxyribose C5′</td><td>U26</td><td>phenyl</td></tr>
</table>

Supplementary Table 15. HIR-6-HIR interactions.

**Hydrogen bonds**				
U25	base	R488	side chain	Hydrogen bond
A33	base	N547	side chain	
G30	base	T320 (L2′)	side chain	
U31	base	R554	side chain	
G30	base	T320 (L2′)	main chain	
U31	base	L552	main chain	
**Salt bridges**				
C22	phosphate	K544	side chain	Salt bridge
U26	phosphate	R479	side chain	
U26	phosphate	R479	side chain	
A28	phosphate	K484	side chain	
**π interactions**				
U31	base	W551	side chain	π-π stacking
U26	phenyl	Y477	side chain	
U25	phenyl	H548	side chain	T-shape π-π stacking
G30	base	E318-K319 (L2′)	main chain	Peptide bond π-π stacking
A32	indole	G550-W551	main chain	
U24	phenyl	Q546-N547	main chain	
U26	base	L486	side chain	CH - π interaction
G30	base	E318 B	side chain	
A28	indole	R479	side chain	
A28	indole	K484	side chain	
A33	indole	L538	side chain	
U31	phenyl	R554	side chain	Cation – π interaction

11. Copies of NMR spectra of prepared compounds

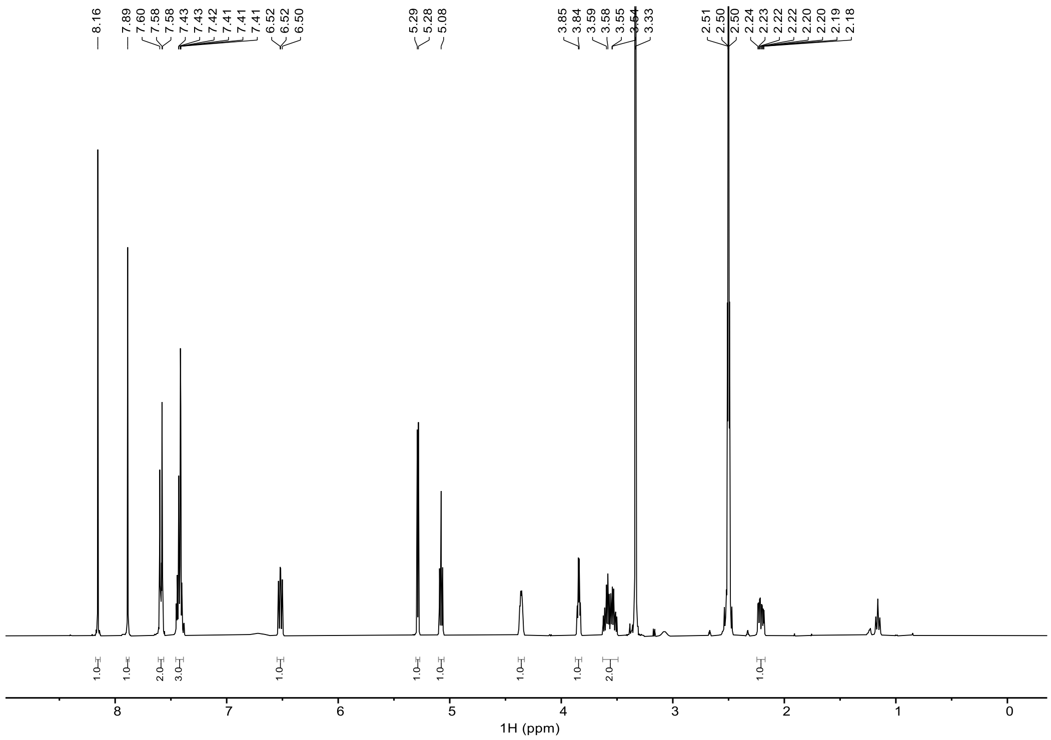

Supplementary Figure 45. ^{1}H NMR spectrum of dA^{EPh}.

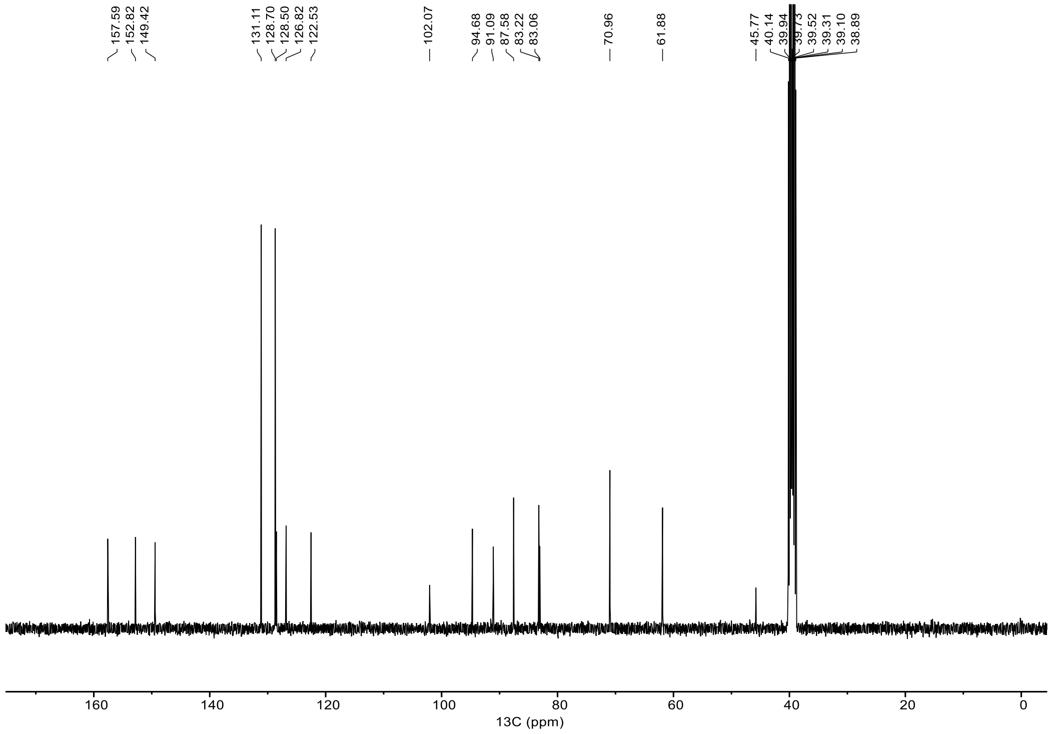

Supplementary Figure 46. ^{13}C NMR spectrum of dA^{EPh}.

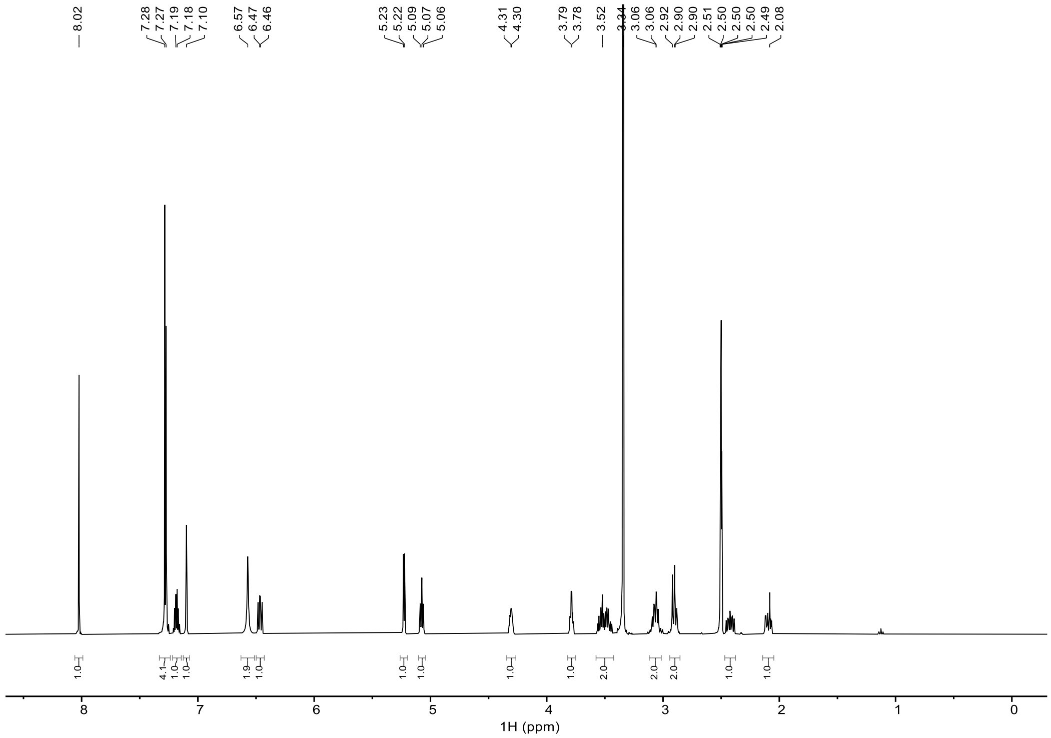

Supplementary Figure 47. ^{1}H NMR spectrum of dA^{APh}.

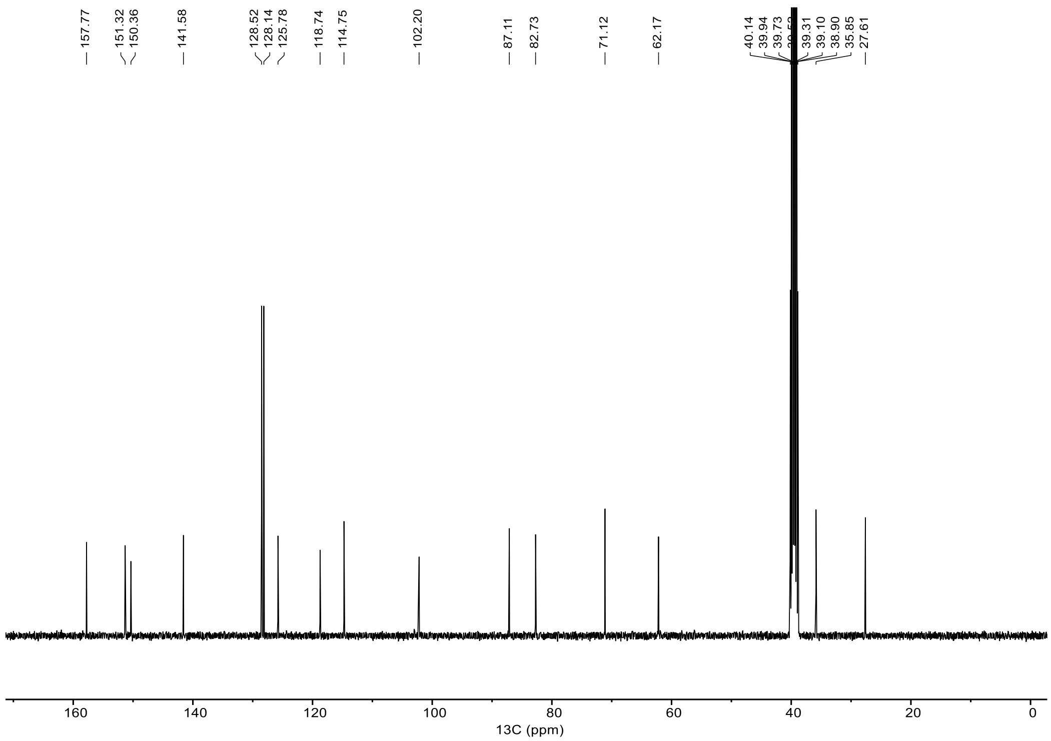

Supplementary Figure 48. ^{13}C NMR spectrum of dA^{APh}.

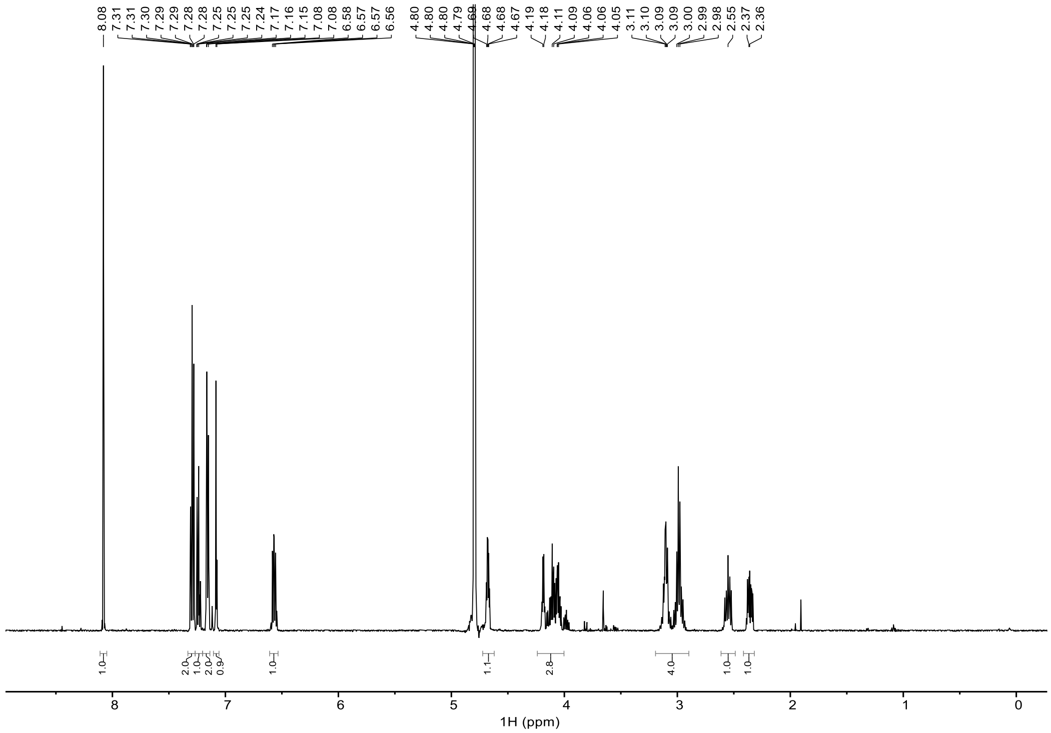

Supplementary Figure 49. ^{1}H NMR spectrum of dA^{APh}TP.

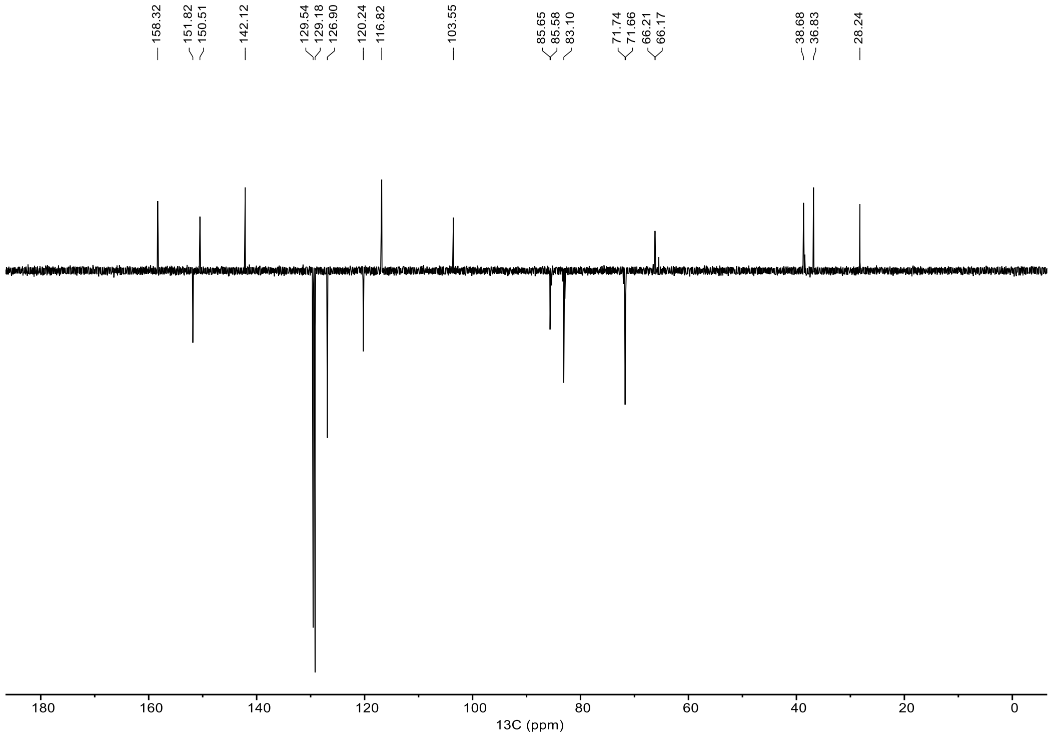

Supplementary Figure 50. ^{13}C APT NMR spectrum of dA^{APh}TP.

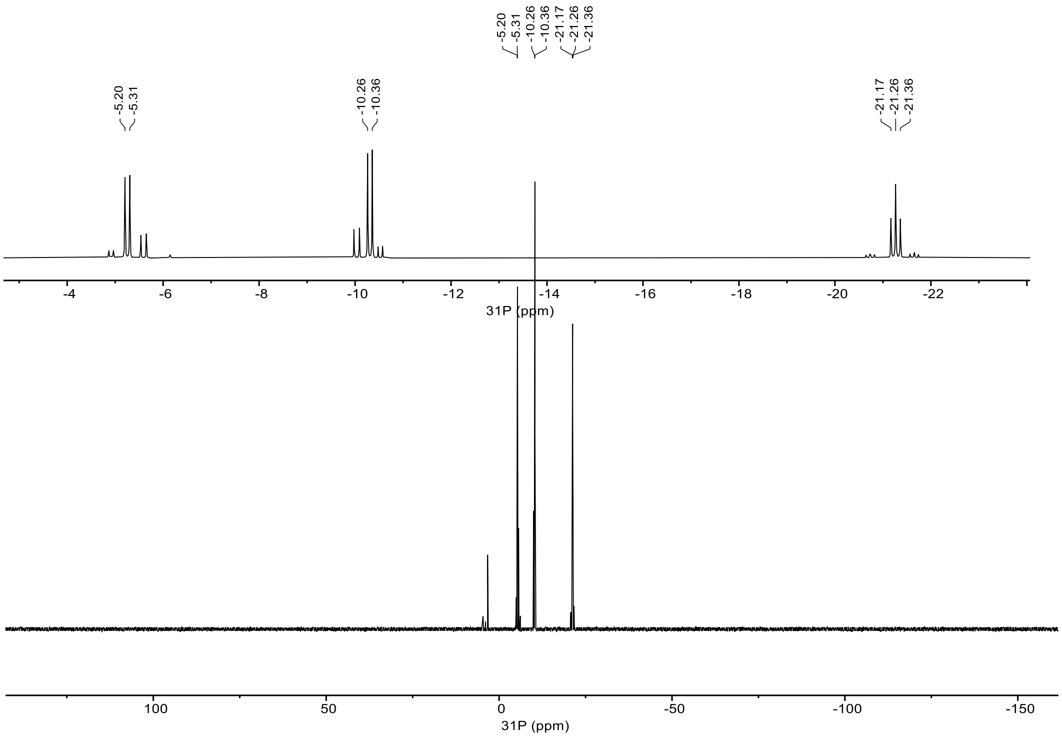

Supplementary Figure 51. ^{31}P NMR spectrum of $dA^{APh}TP$.

12. MS characterization of synthesized modified ONs

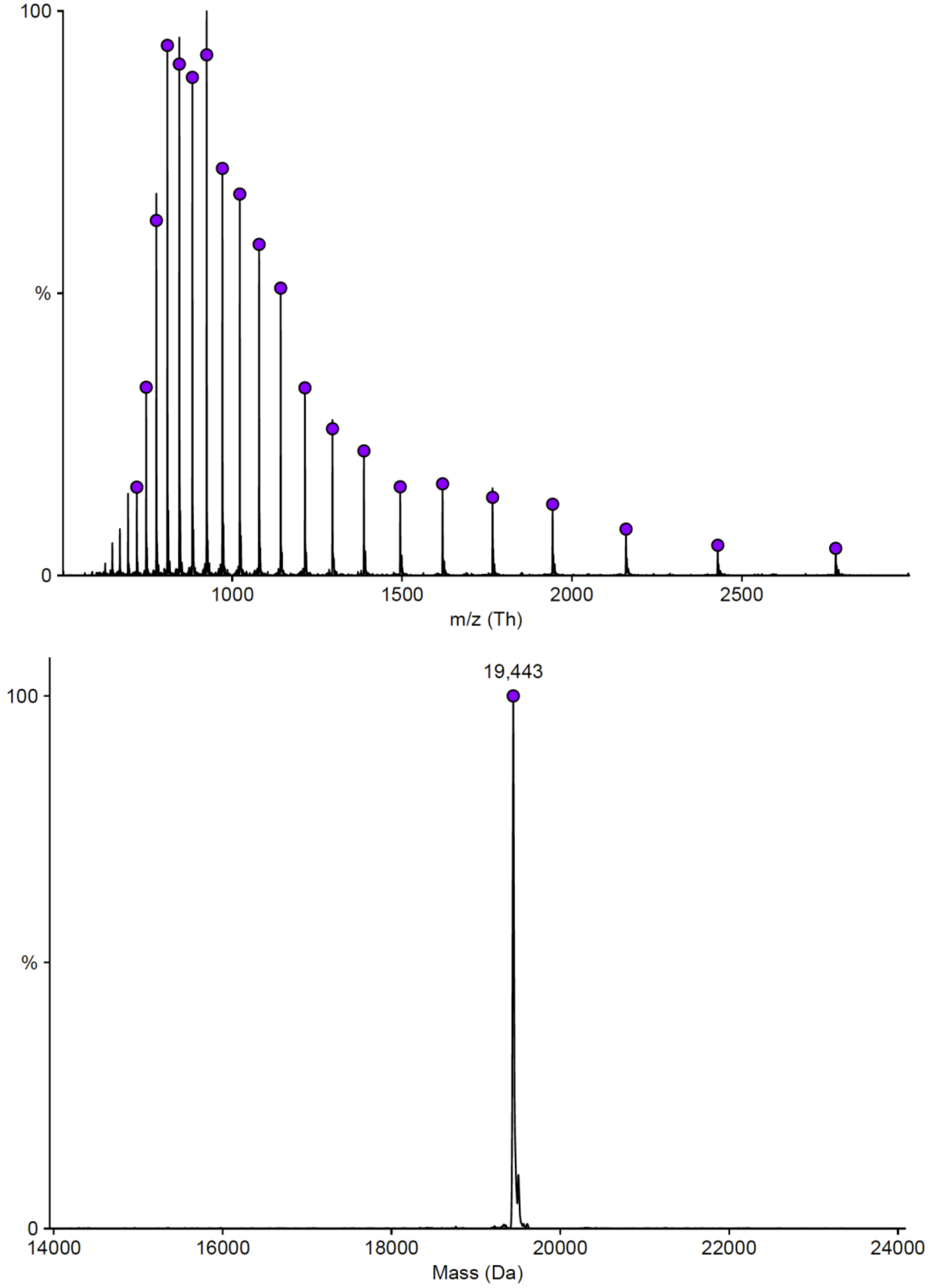

Supplementary Figure 52. Raw and deconvoluted MS spectrum of 5´-Cy5-HIR-6, calculated mass: 19445 Da, found mass: 19443 Da.

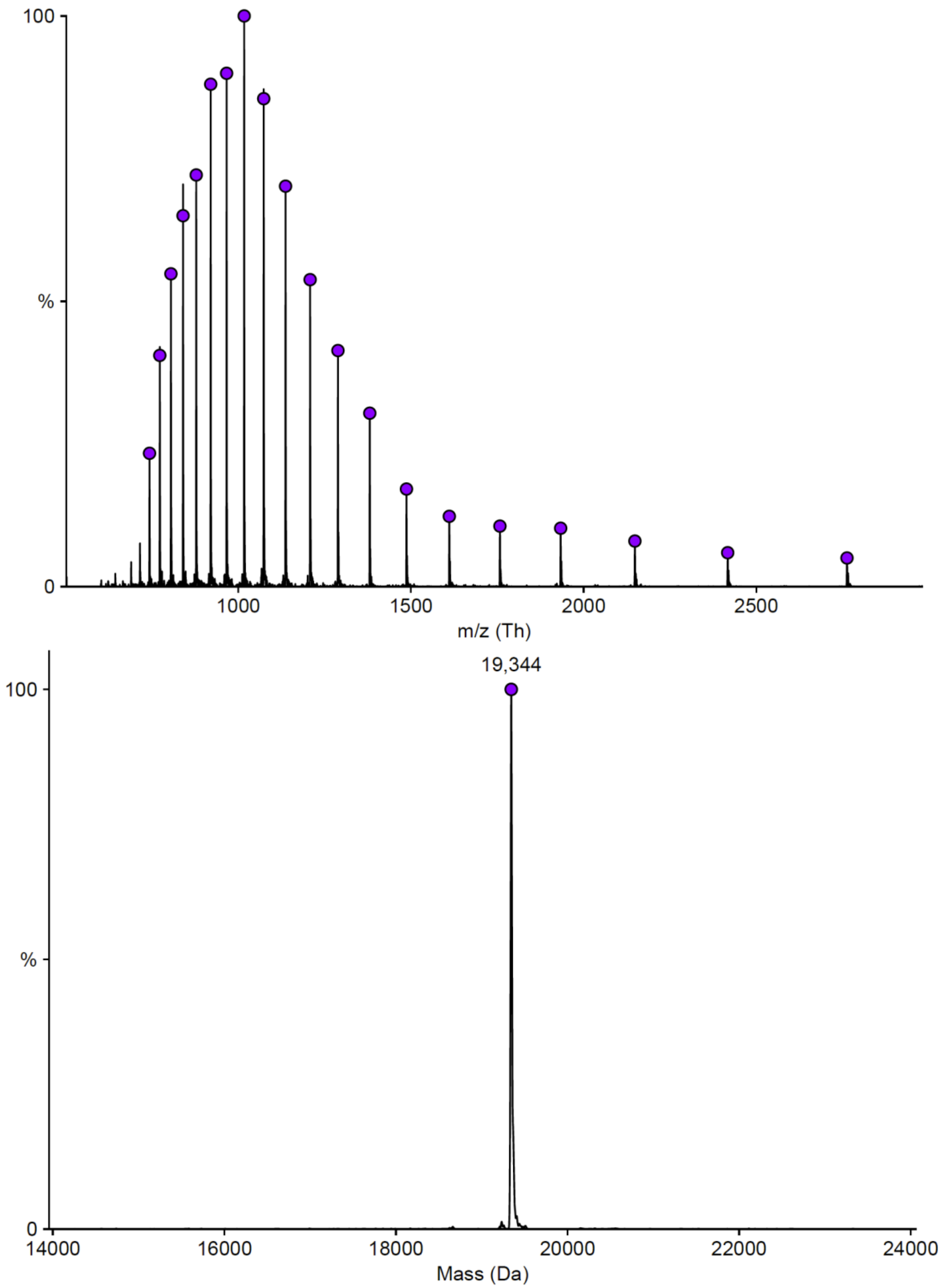

Supplementary Figure 53. Raw and deconvoluted MS spectrum of 5´-Biotin-HIR-6, calculated mass: 19349 Da, found mass: 19344 Da.

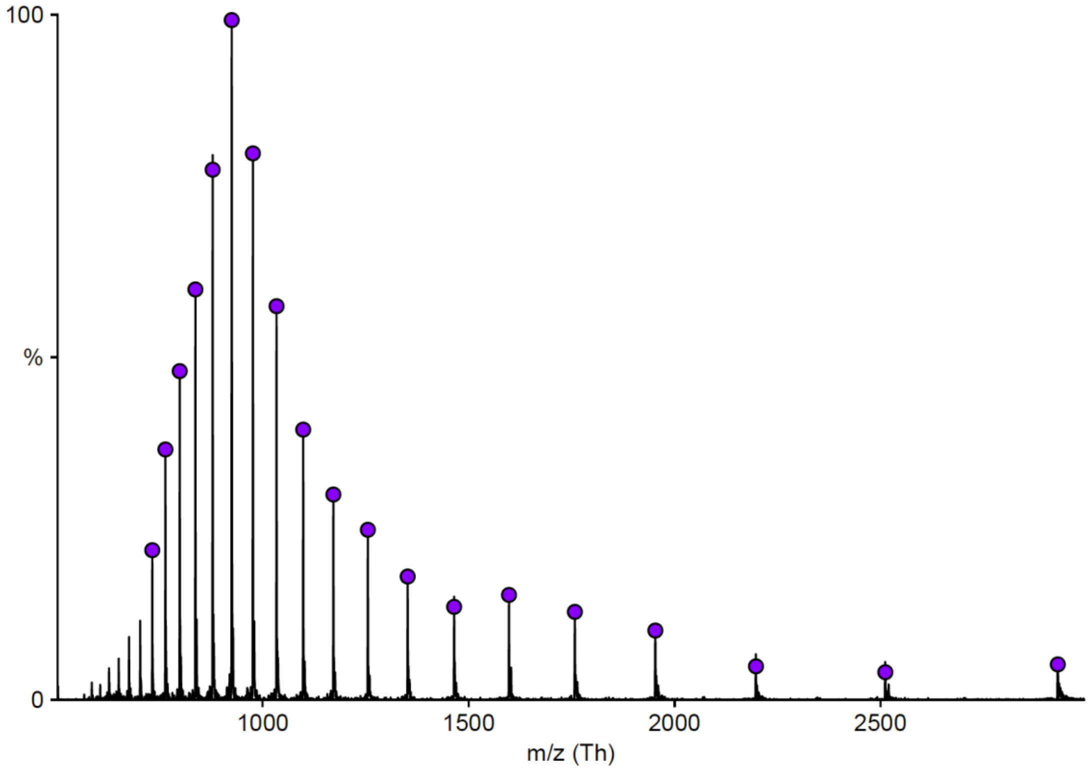

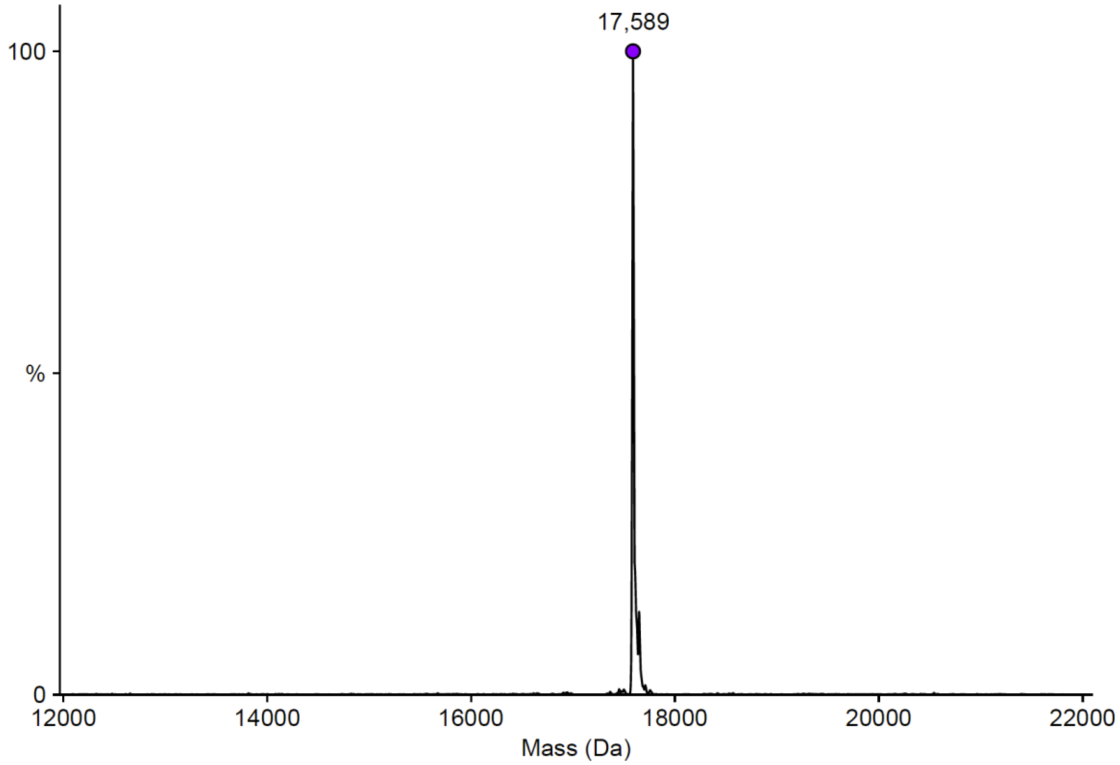

Supplementary Figure 54. Raw and deconvoluted MS spectrum of 5´-Cy5-HIR-6_T1, calculated mass: 17591 Da, found mass: 17589 Da.

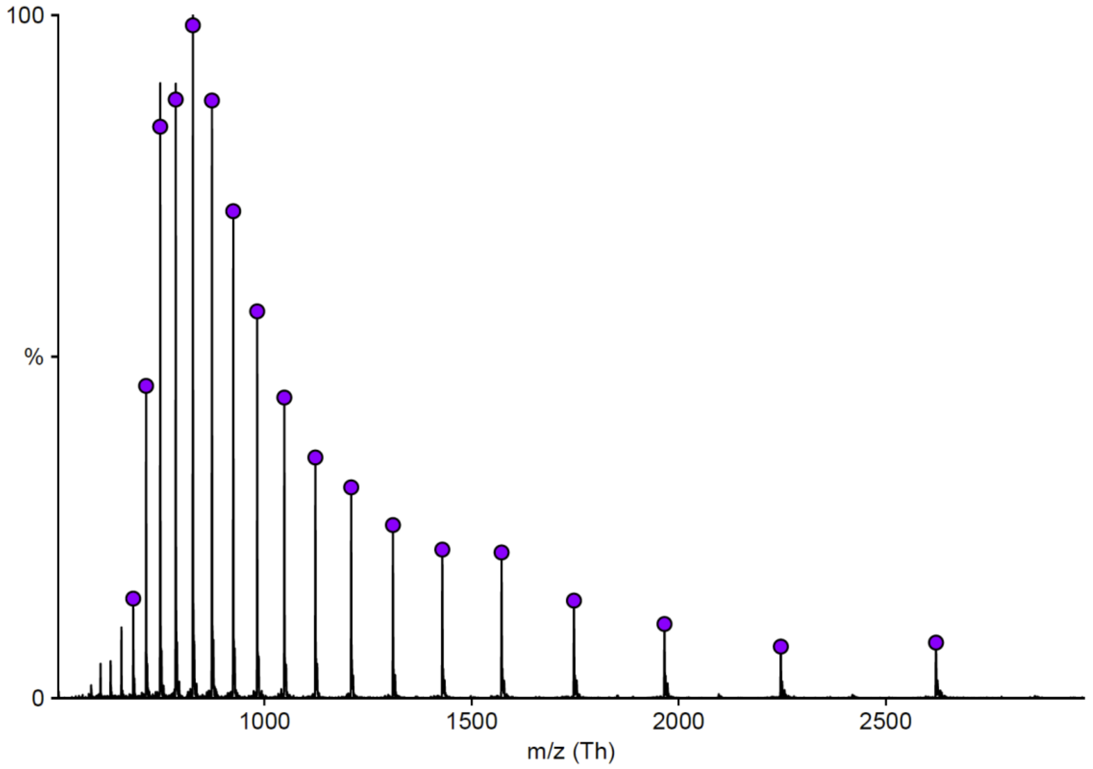

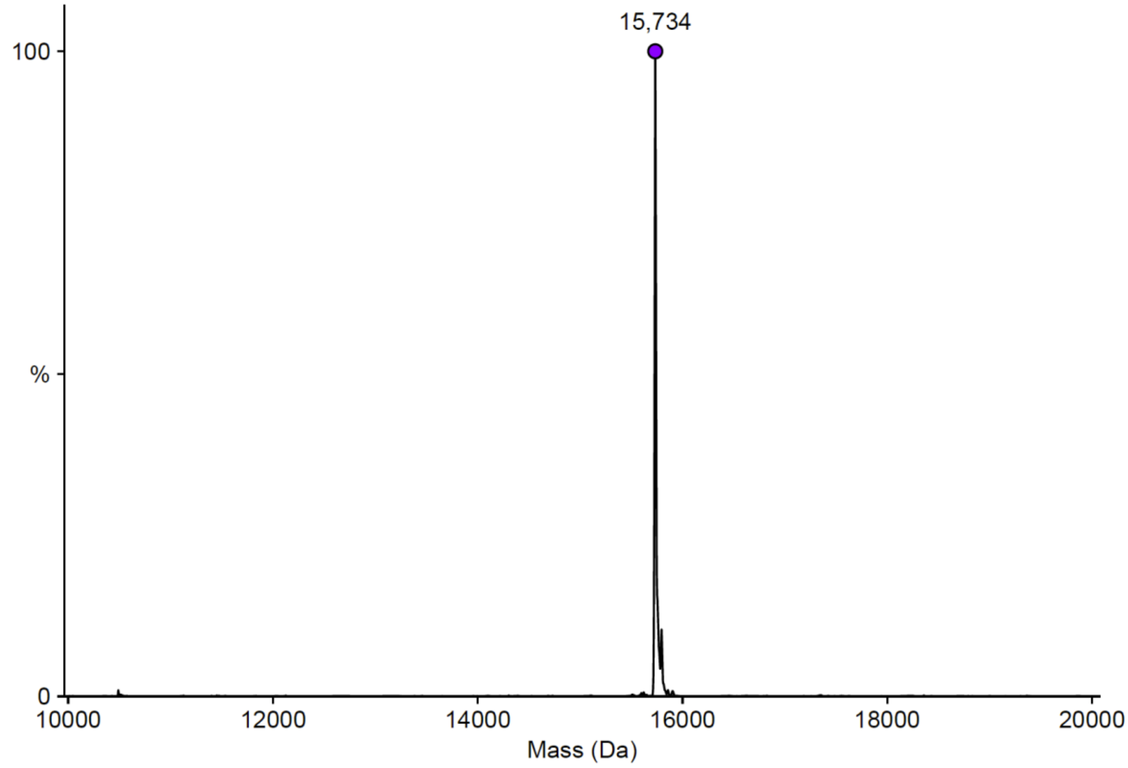

Supplementary Figure 55. Raw and deconvoluted MS spectrum of 5´-Cy5-HIR-6_T2, calculated mass: 15737 Da, found mass: 15734 Da.

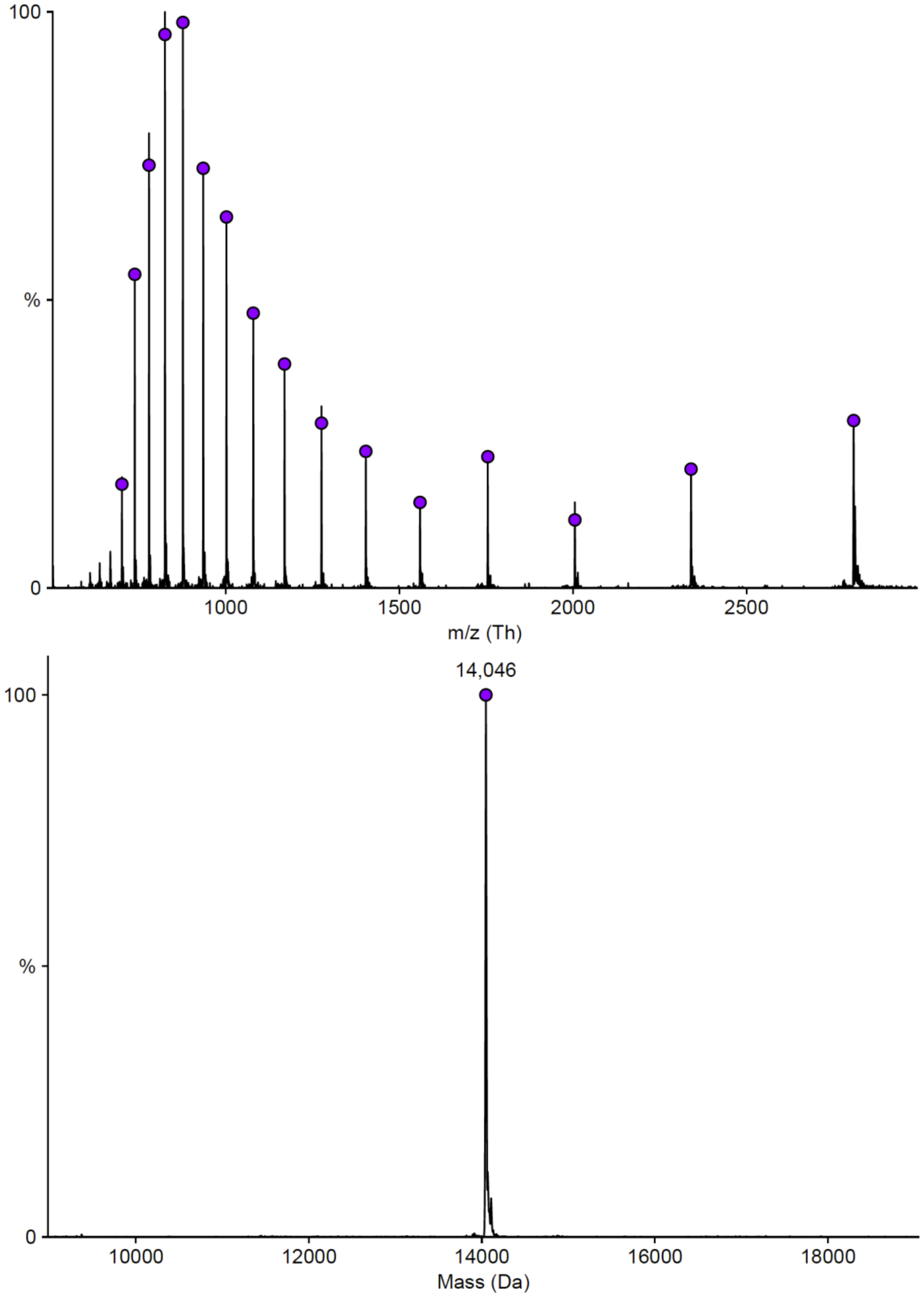

Supplementary Figure 56. Raw and deconvoluted MS spectrum of 5´-Cy5-HIR-6_T3, calculated mass: 14048 Da, found mass: 14046 Da.

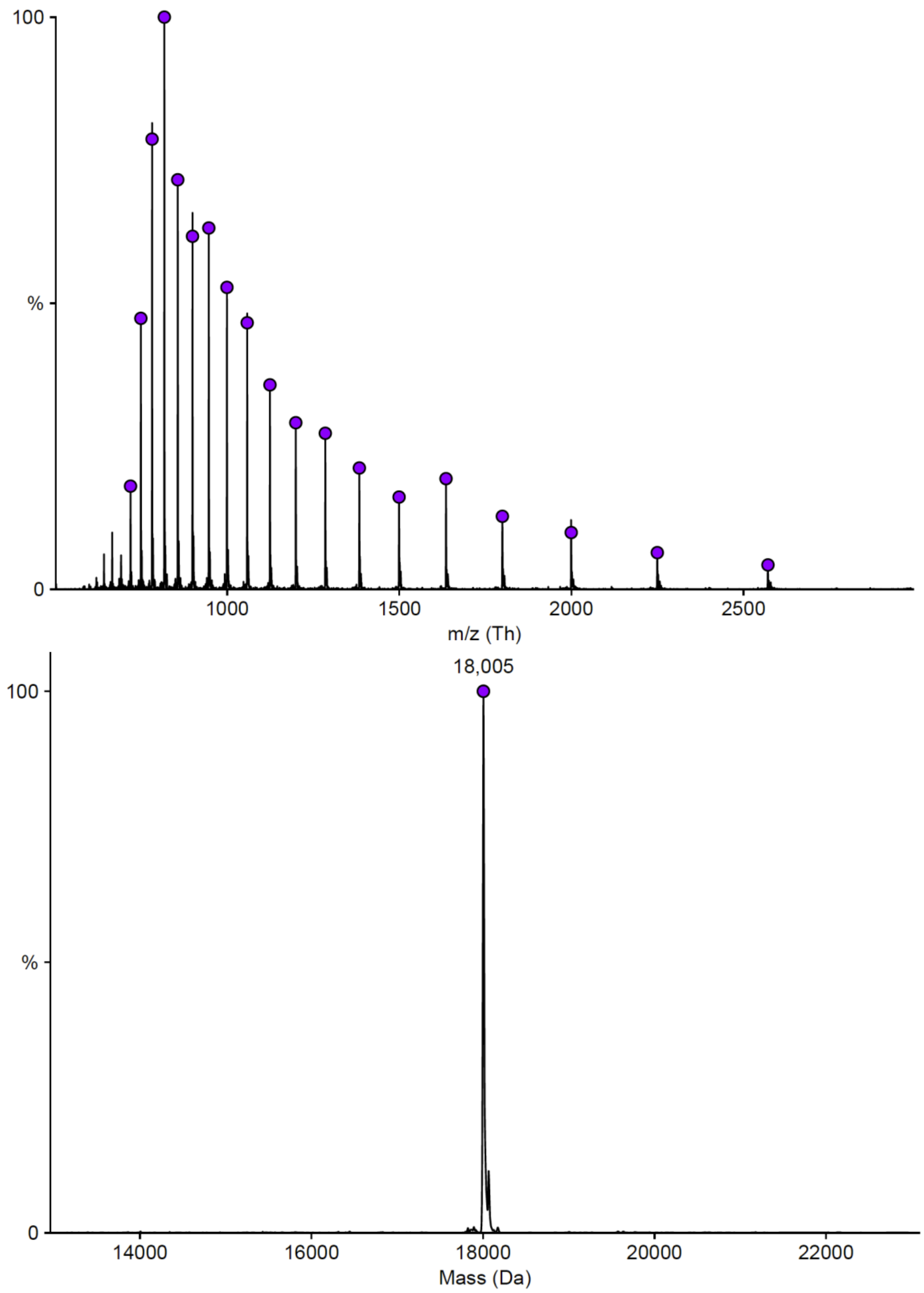

Supplementary Figure 57. Raw and deconvoluted MS spectrum of 5´-Cy5-HIR-6_T4, calculated mass: 18008 Da, found mass: 18005 Da.

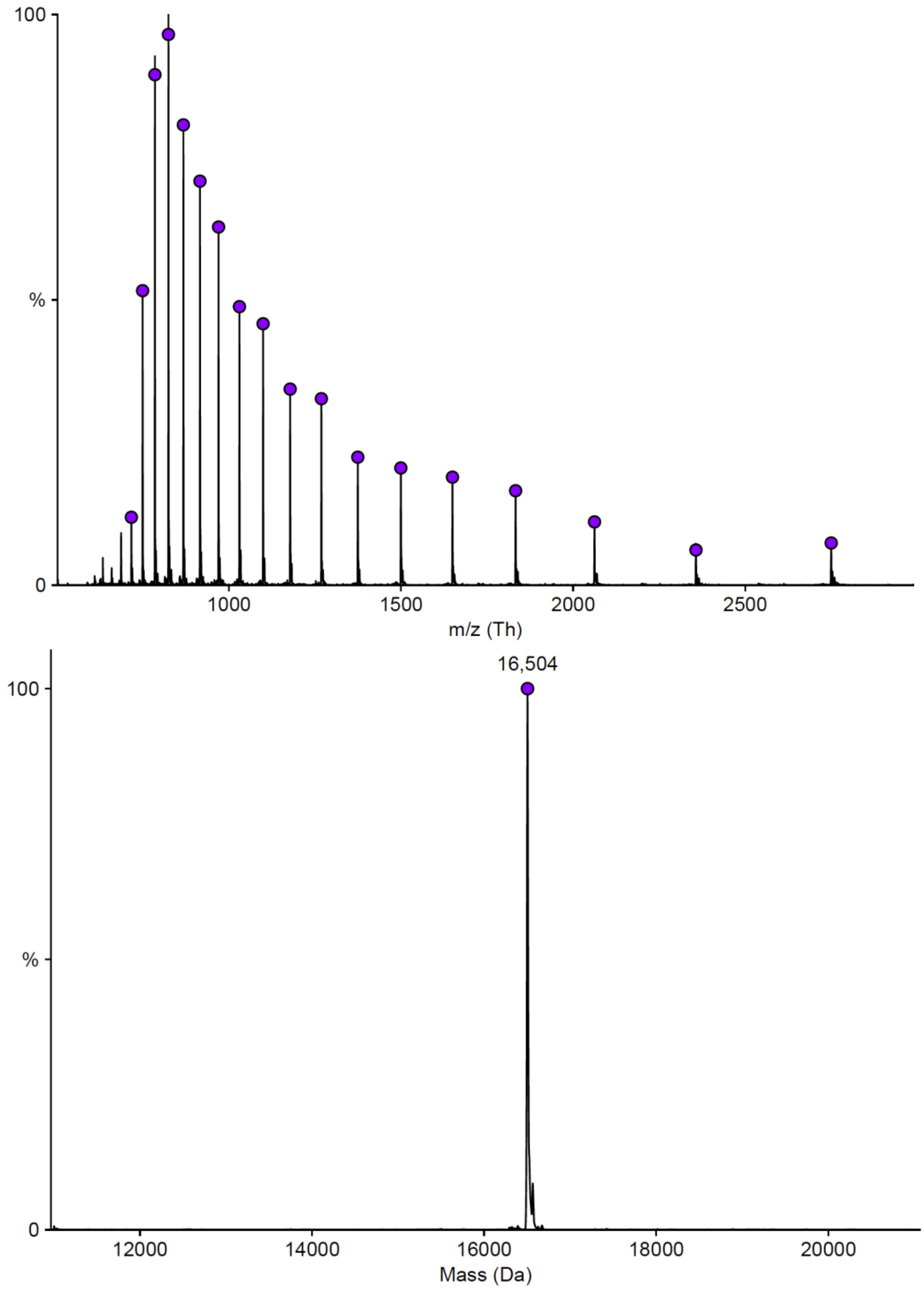

Supplementary Figure 58. Raw and deconvoluted MS spectrum of 5´-Cy5-HIR-6_T5, calculated mass: 16507 Da, found mass: 16504 Da.

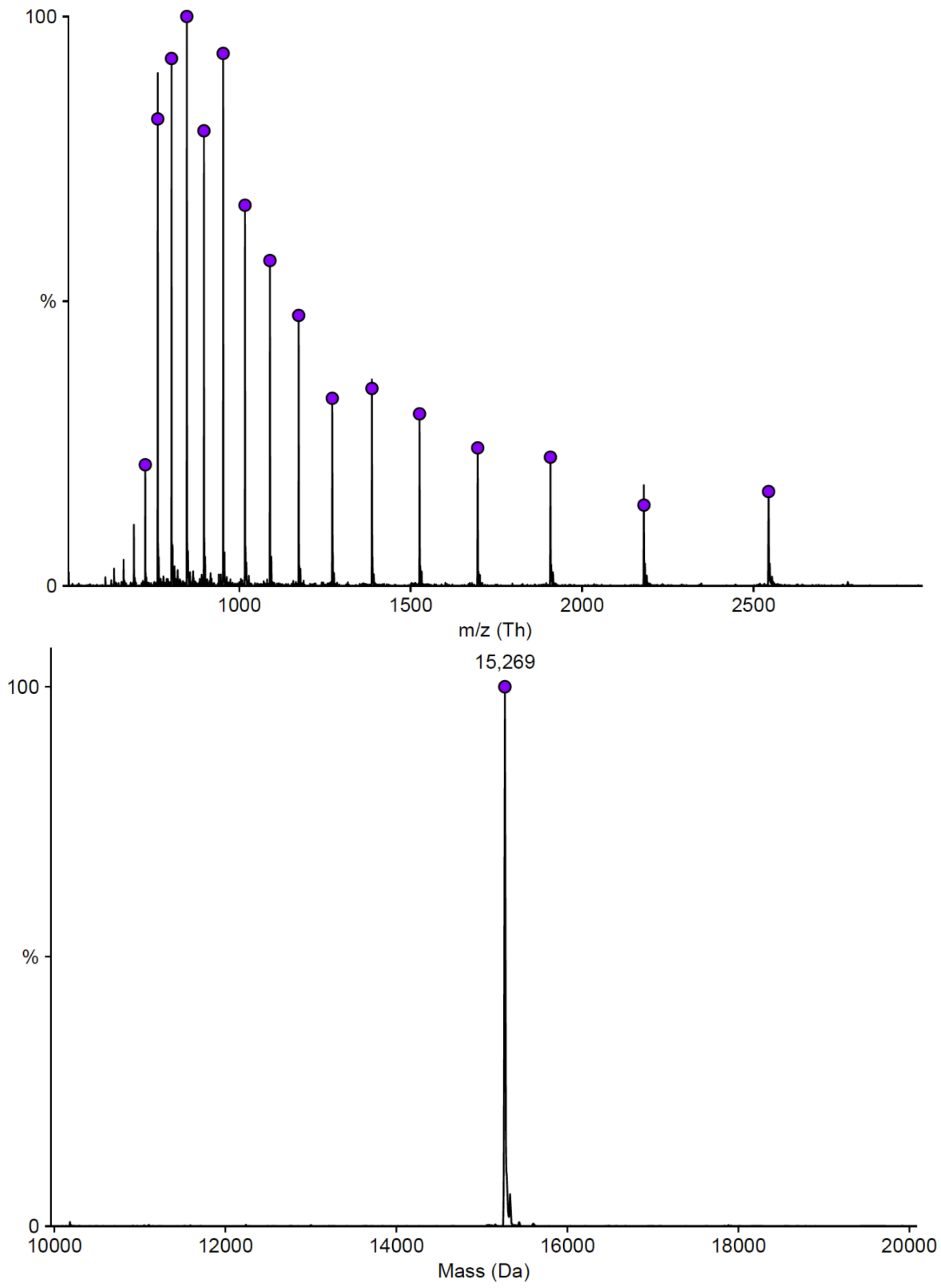

Supplementary Figure 59. Raw and deconvoluted MS spectrum of 5´-Cy5-HIR-6_T6, calculated mass: 15271 Da, found mass: 15269 Da.

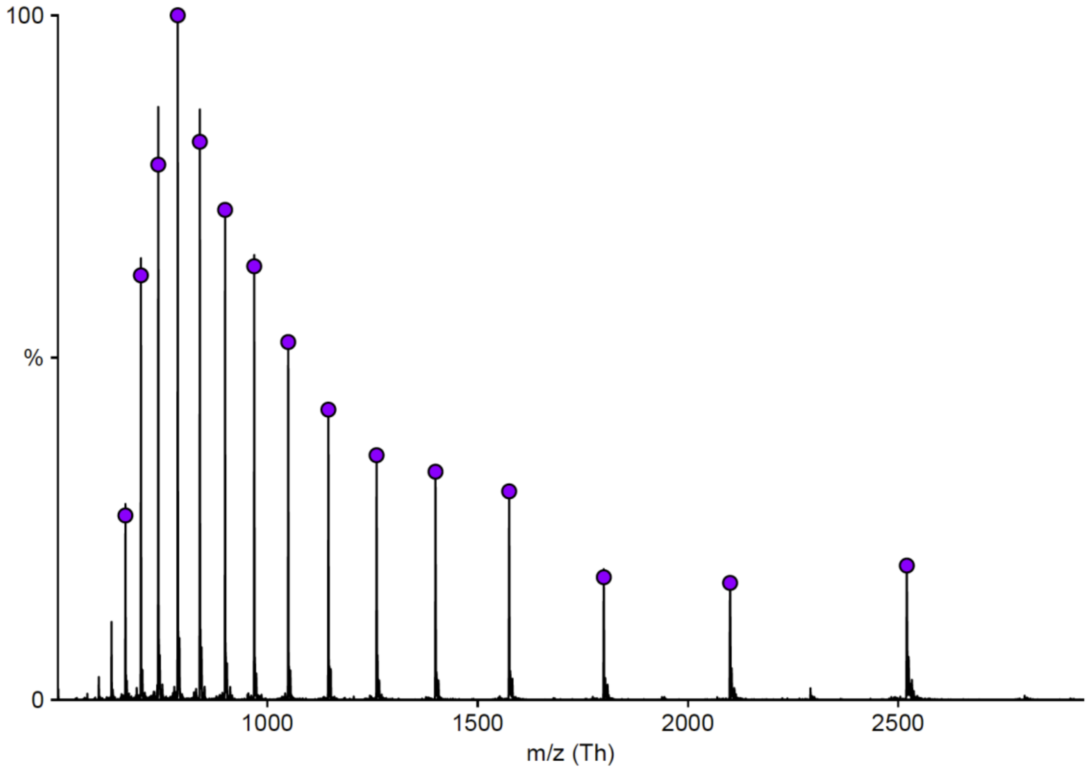

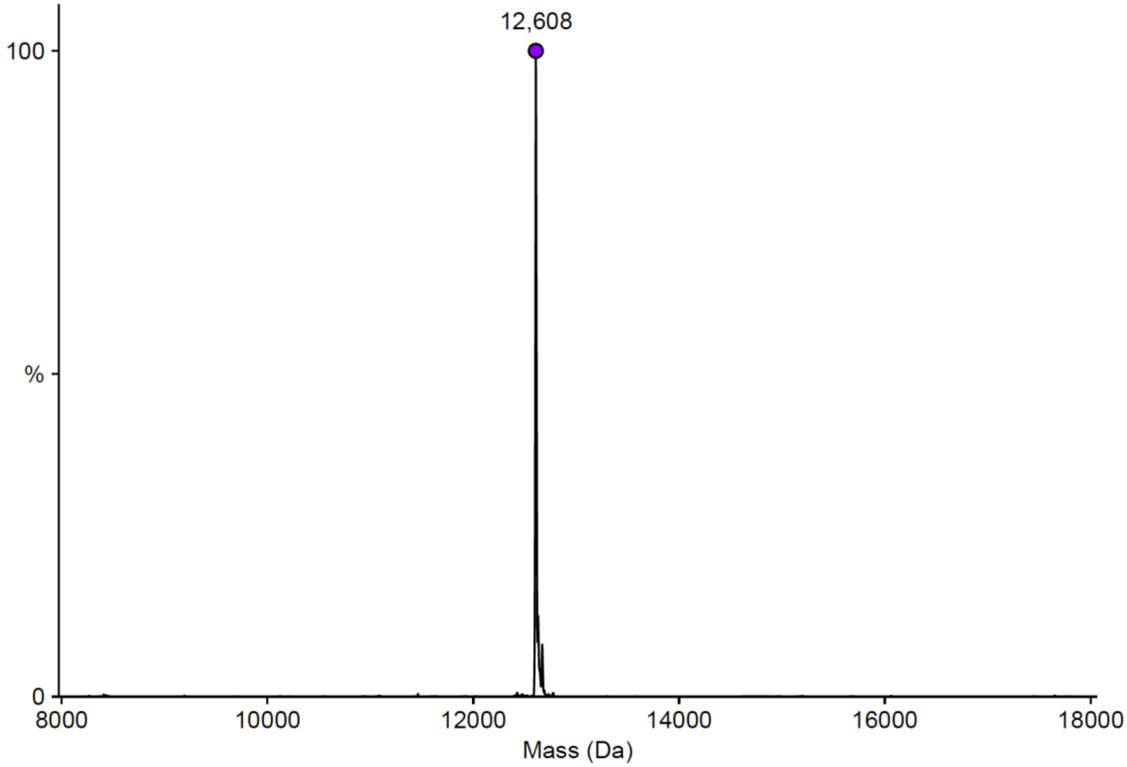

Supplementary Figure 60. Raw and deconvoluted MS spectrum of 5´-Cy5-HIR-6_T7, calculated mass: 12611 Da, found mass: 12608 Da.

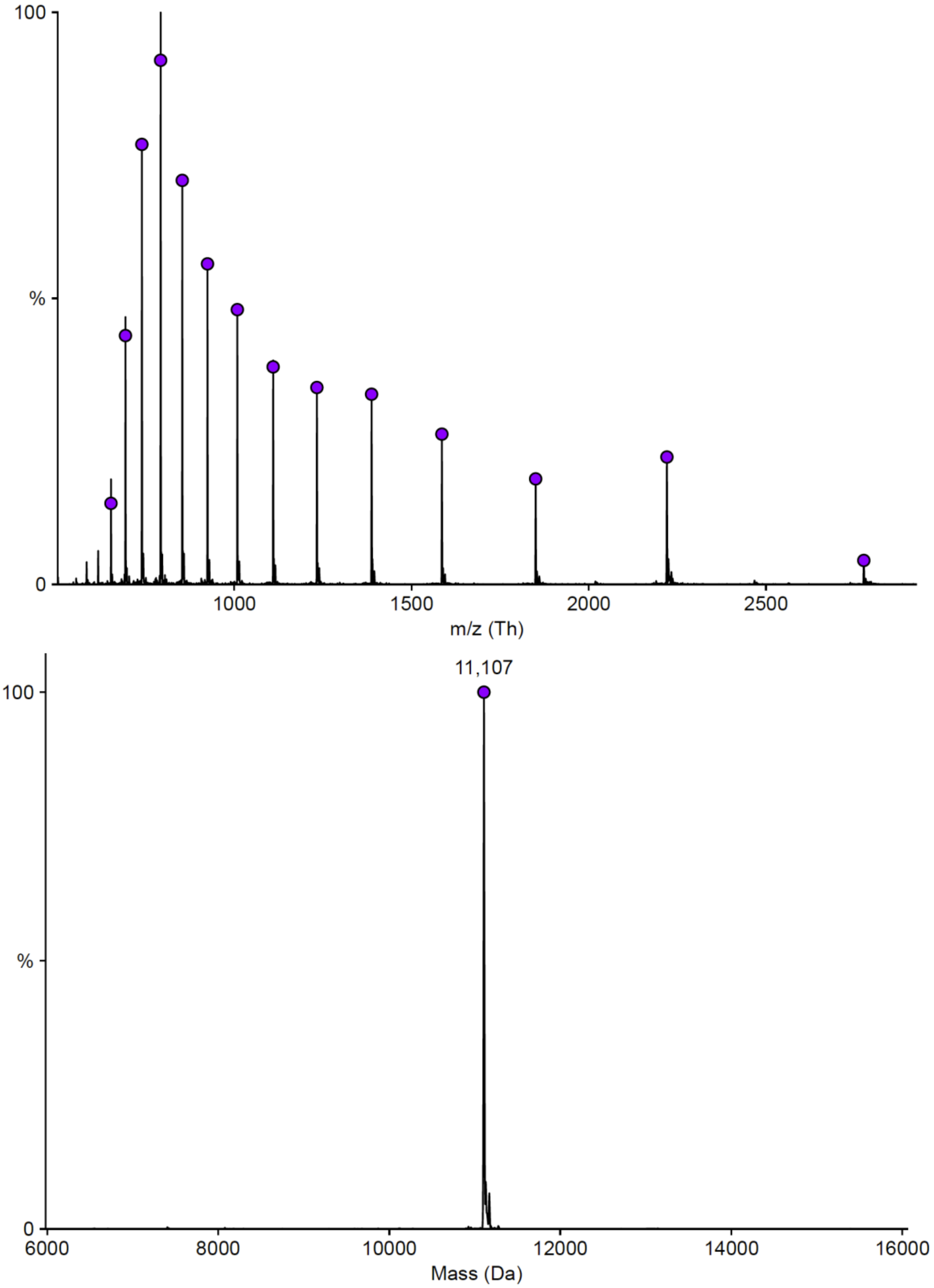

Supplementary Figure 61. Raw and deconvoluted MS spectrum of 5´-Cy5-HIR-6_T8, calculated mass: 11110 Da, found mass: 11107 Da.

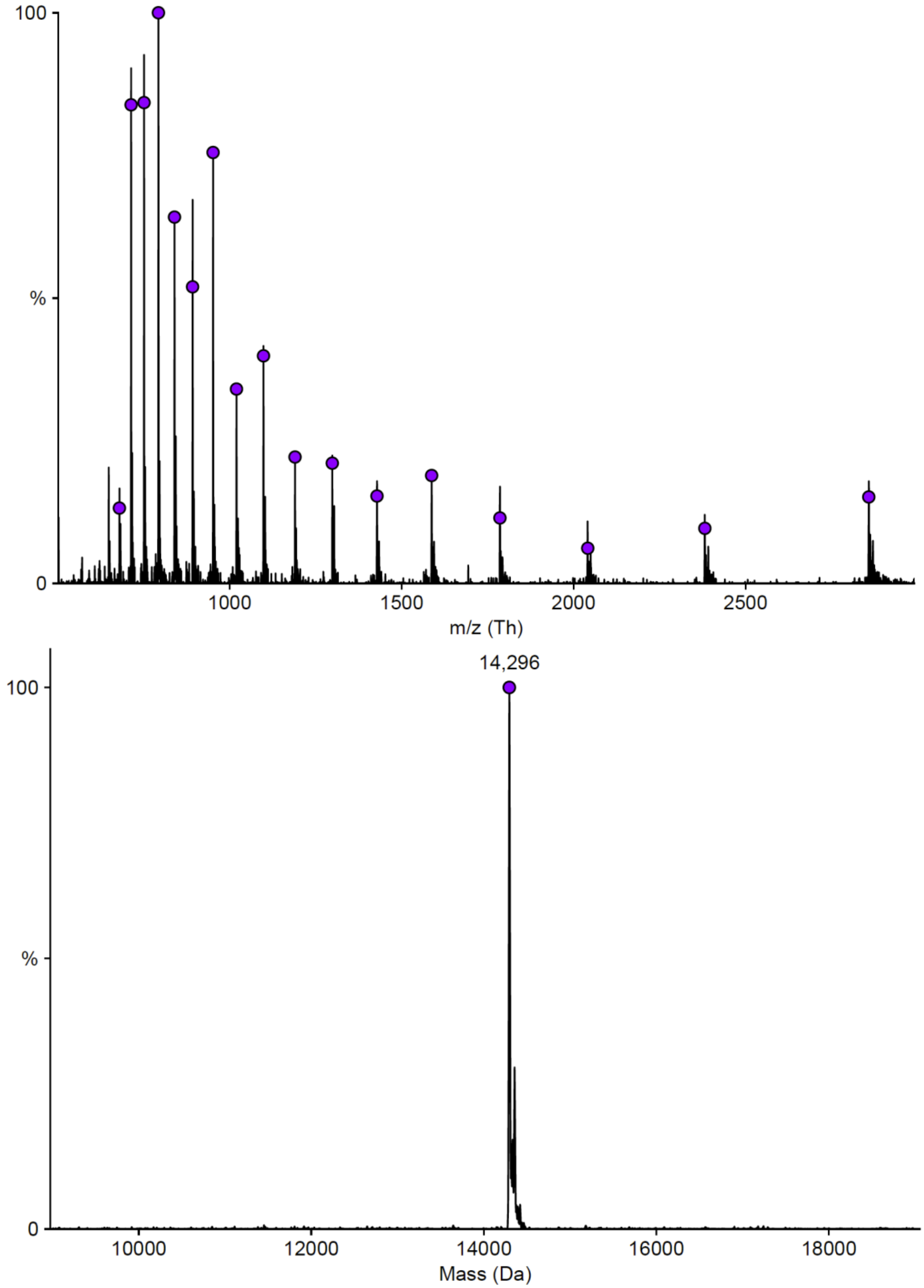

Supplementary Figure 62. Raw and deconvoluted MS spectrum of 5´-Cy5-HIR-6_T9, calculated mass: 14299 Da, found mass: 14296 Da.

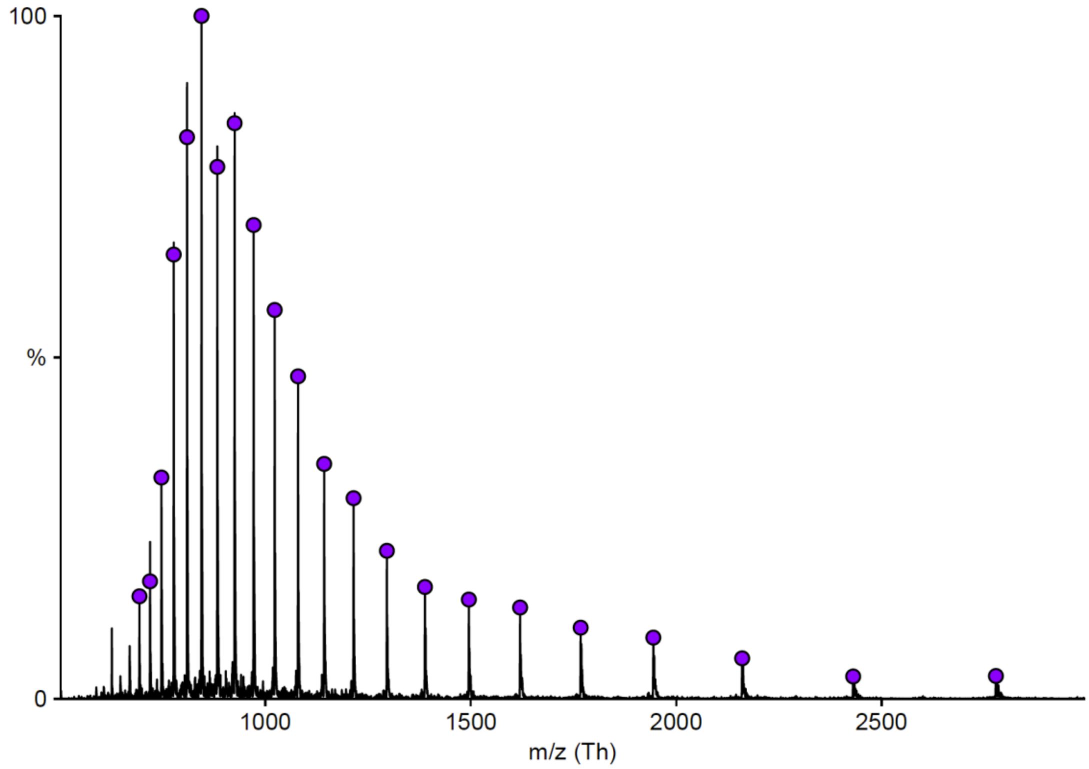

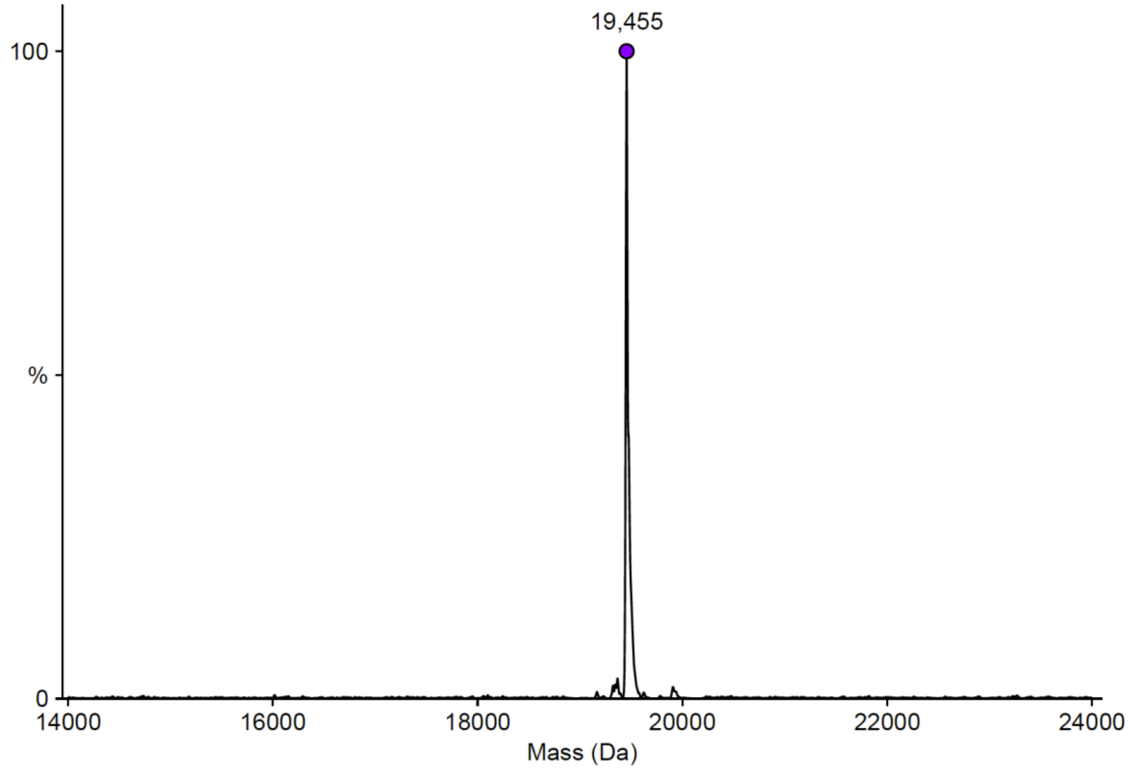

Supplementary Figure 63. Raw and deconvoluted MS spectrum of 5´-Cy5-HIR-6_V1, calculated mass: 19457 Da, found mass: 19455 Da.

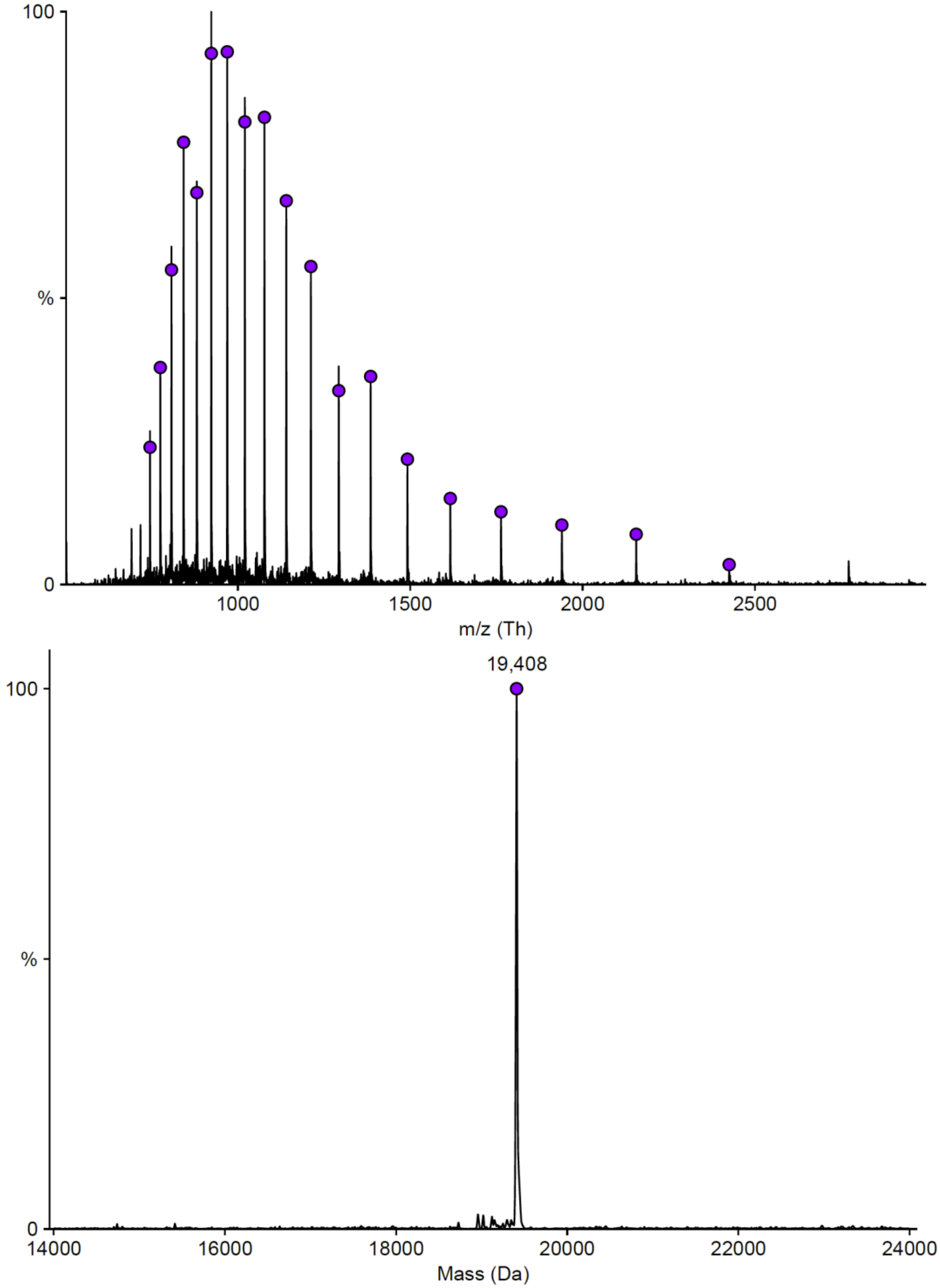

Supplementary Figure 64. Raw and deconvoluted MS spectrum of 5´-Cy5-HIR-6_V2, calculated mass: 19413 Da, found mass: 19408 Da.

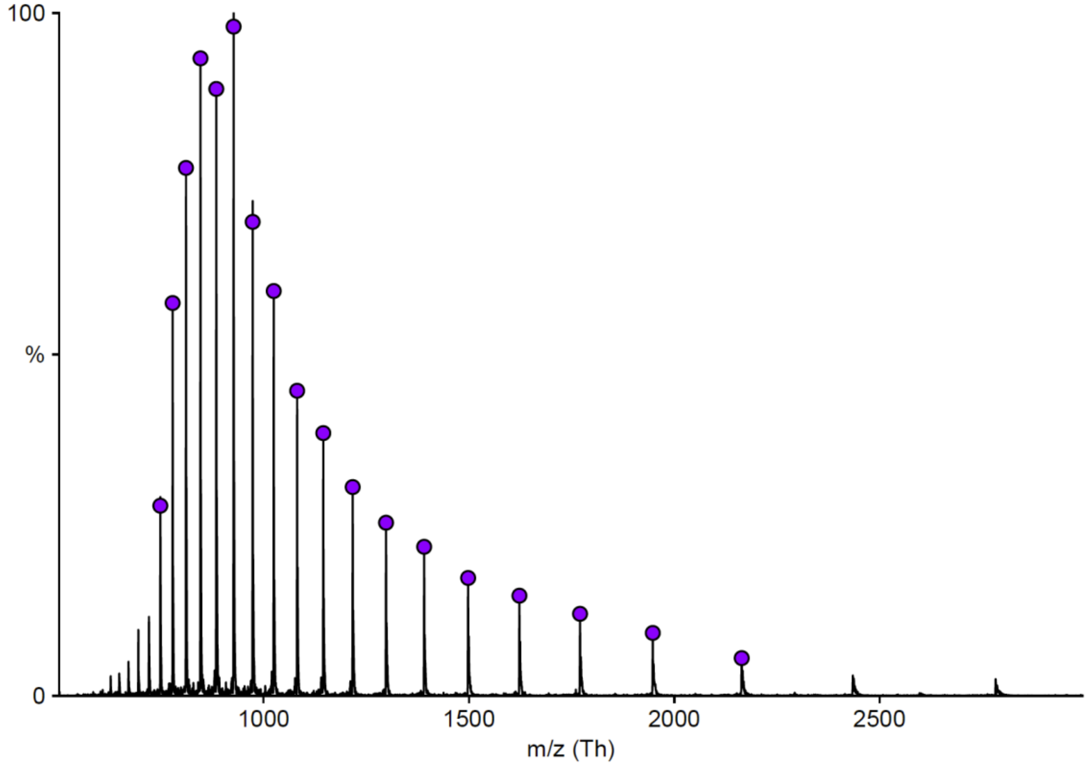

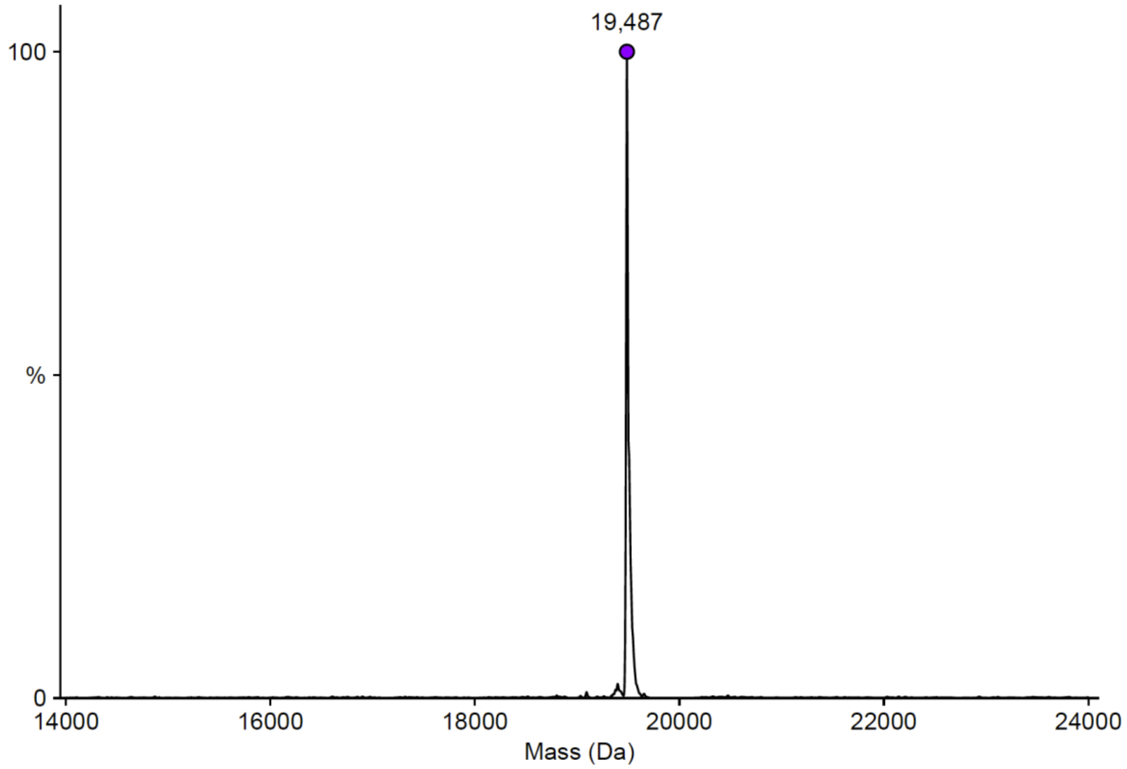

Supplementary Figure 65. Raw and deconvoluted MS spectrum of 5´-Cy5-HIR-6_V3, calculated mass: 19490 Da, found mass: 19487 Da.

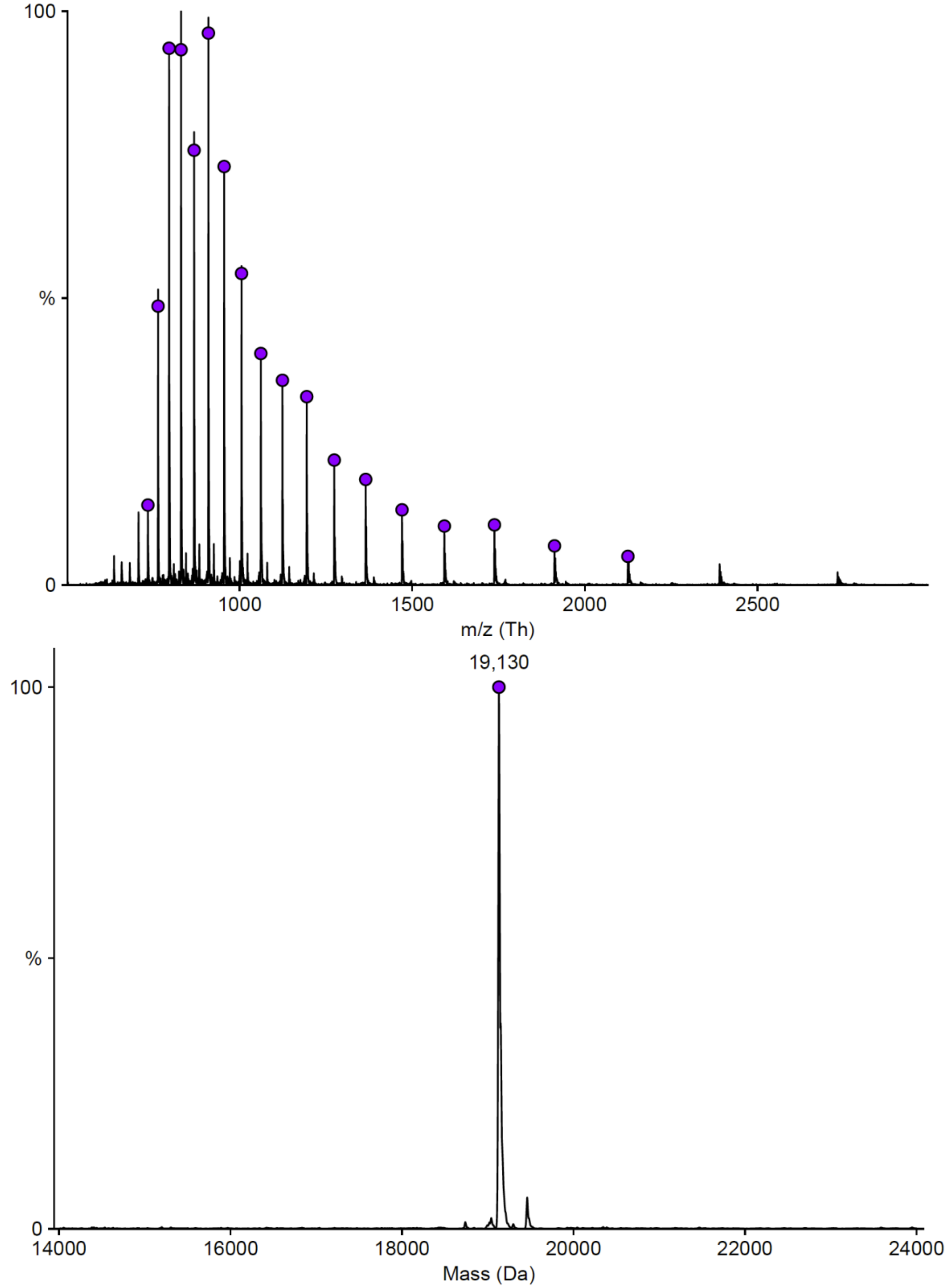

Supplementary Figure 66. Raw and deconvoluted MS spectrum of 5´-Cy5-HIR-6_V4, calculated mass: 19133 Da, found mass: 19130 Da.

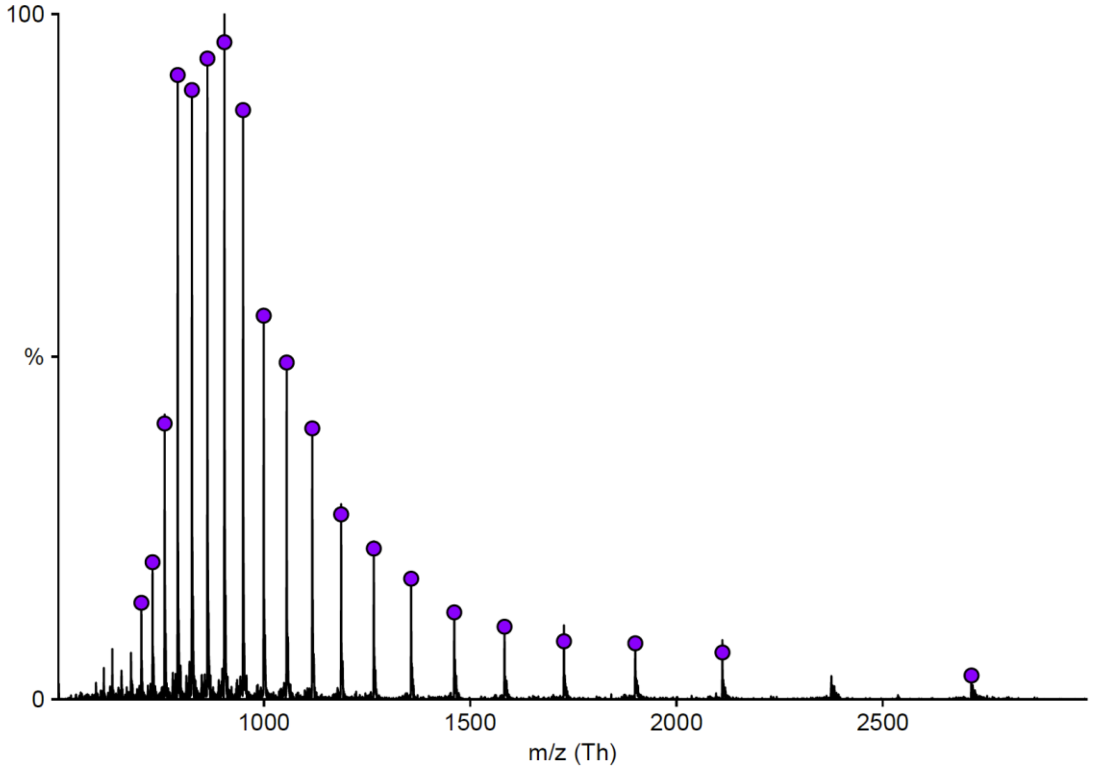

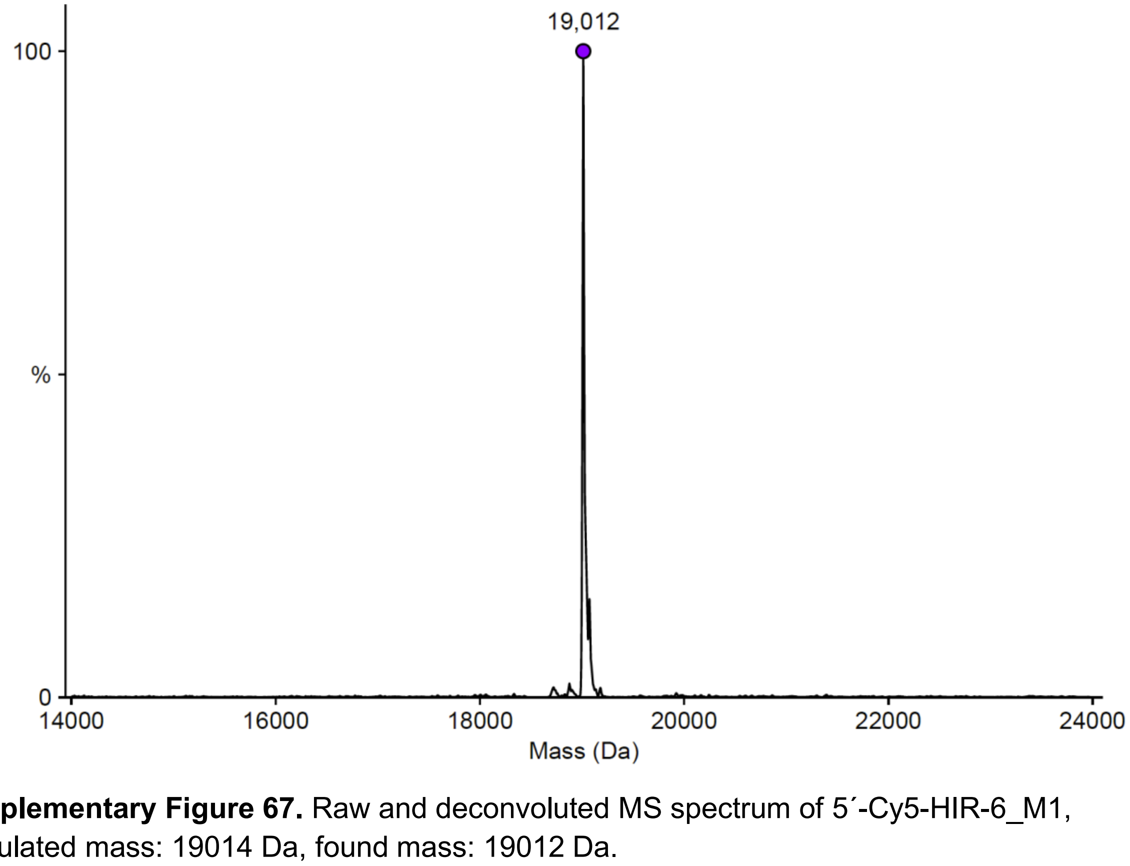

Supplementary Figure 67. Raw and deconvoluted MS spectrum of 5´-Cy5-HIR-6_M1, calculated mass: 19014 Da, found mass: 19012 Da.

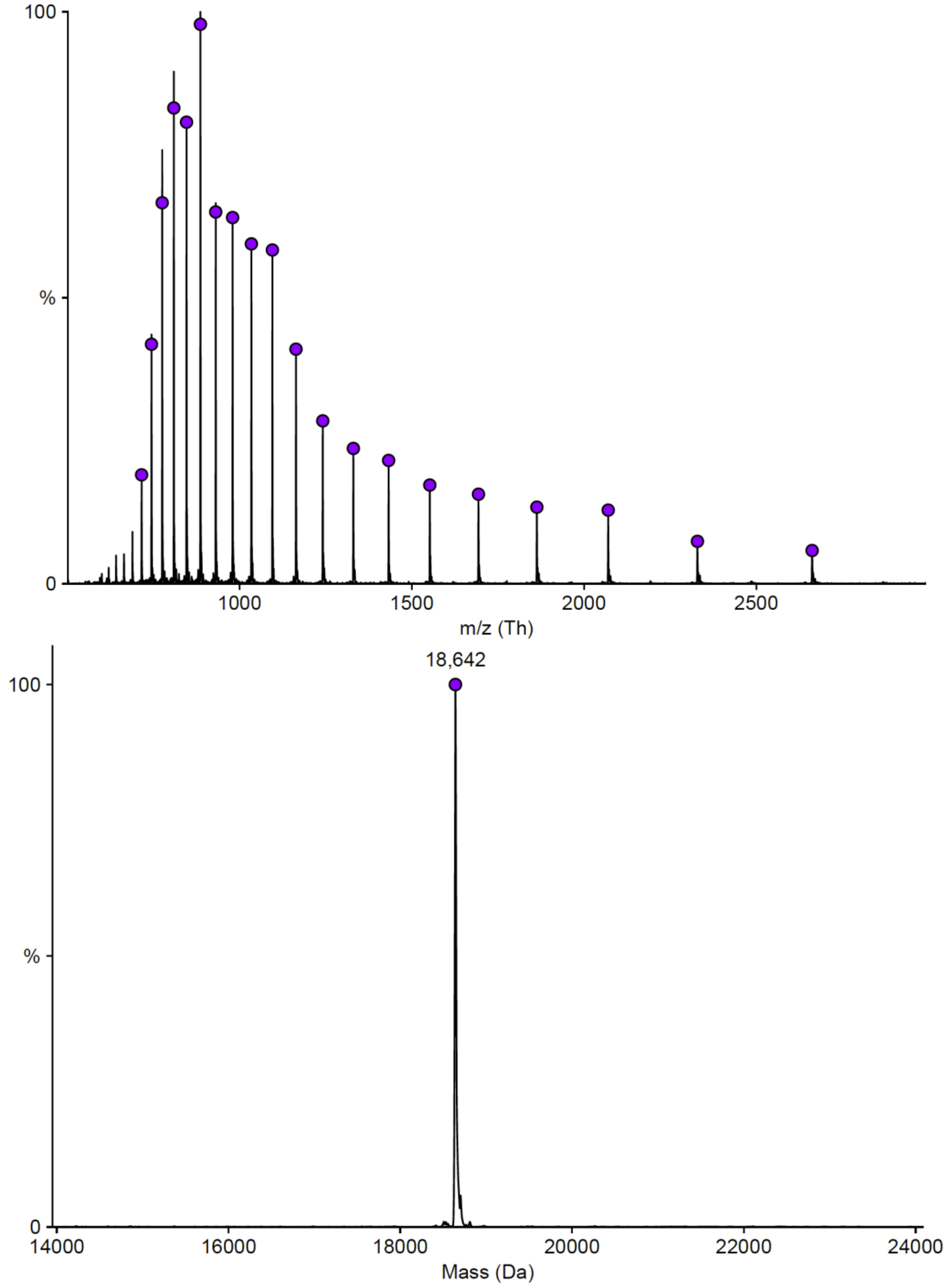

Supplementary Figure 68. Raw and deconvoluted MS spectrum of 5´-Cy5-HIR-6_M2, calculated mass: 18644 Da, found mass: 18642 Da.

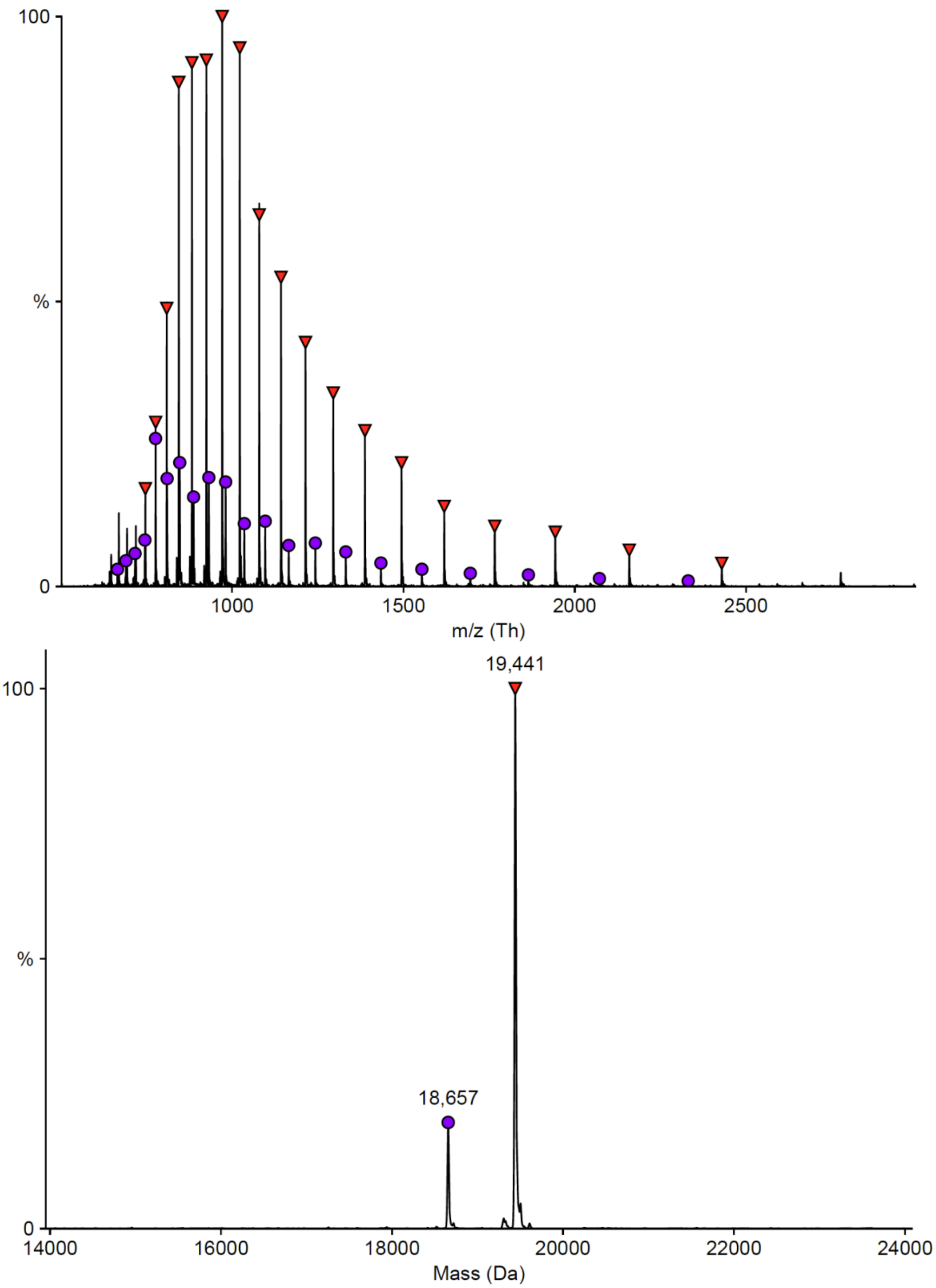

Supplementary Figure 69. Raw and deconvoluted MS spectrum of 5´-Cy5-HIR_SC, calculated mass: 19445 Da, found mass: 19441 Da. The mass of 18657 Da corresponds to the N-2 product (calculated mass: 18661 Da).

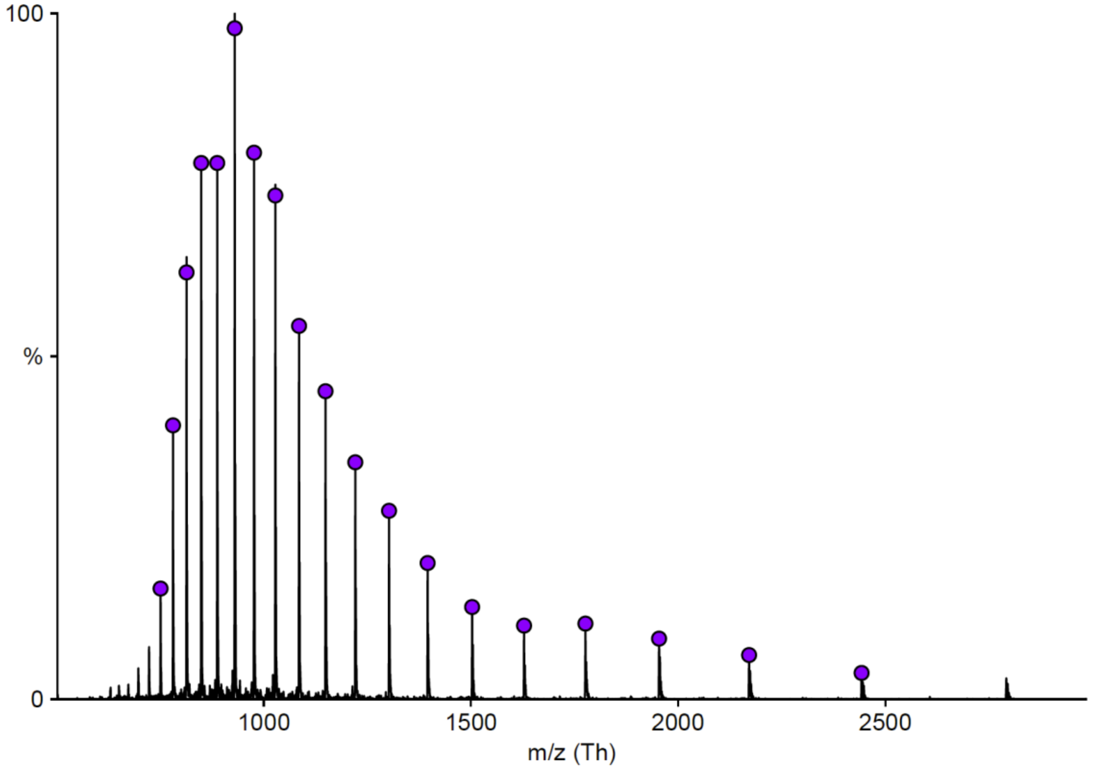

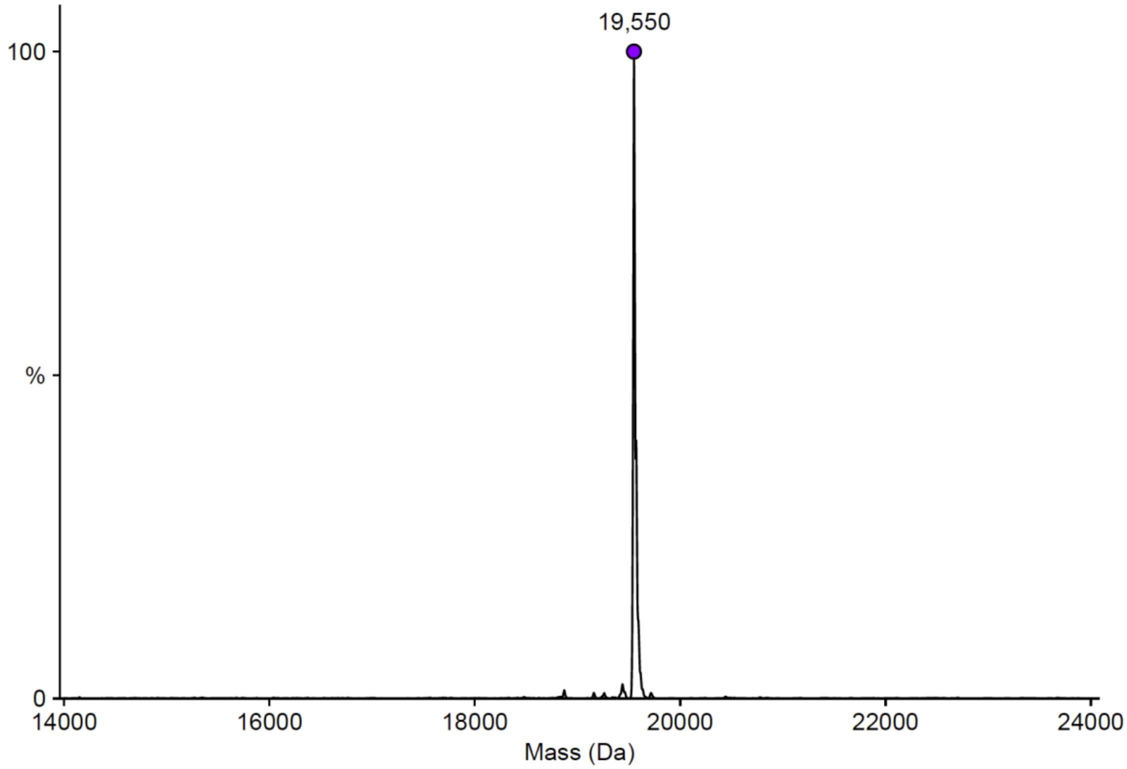

Supplementary Figure 70. Raw and deconvoluted MS spectrum of 5´-Cy5-HIR-8, calculated mass: 19557 Da, found mass: 19550 Da.

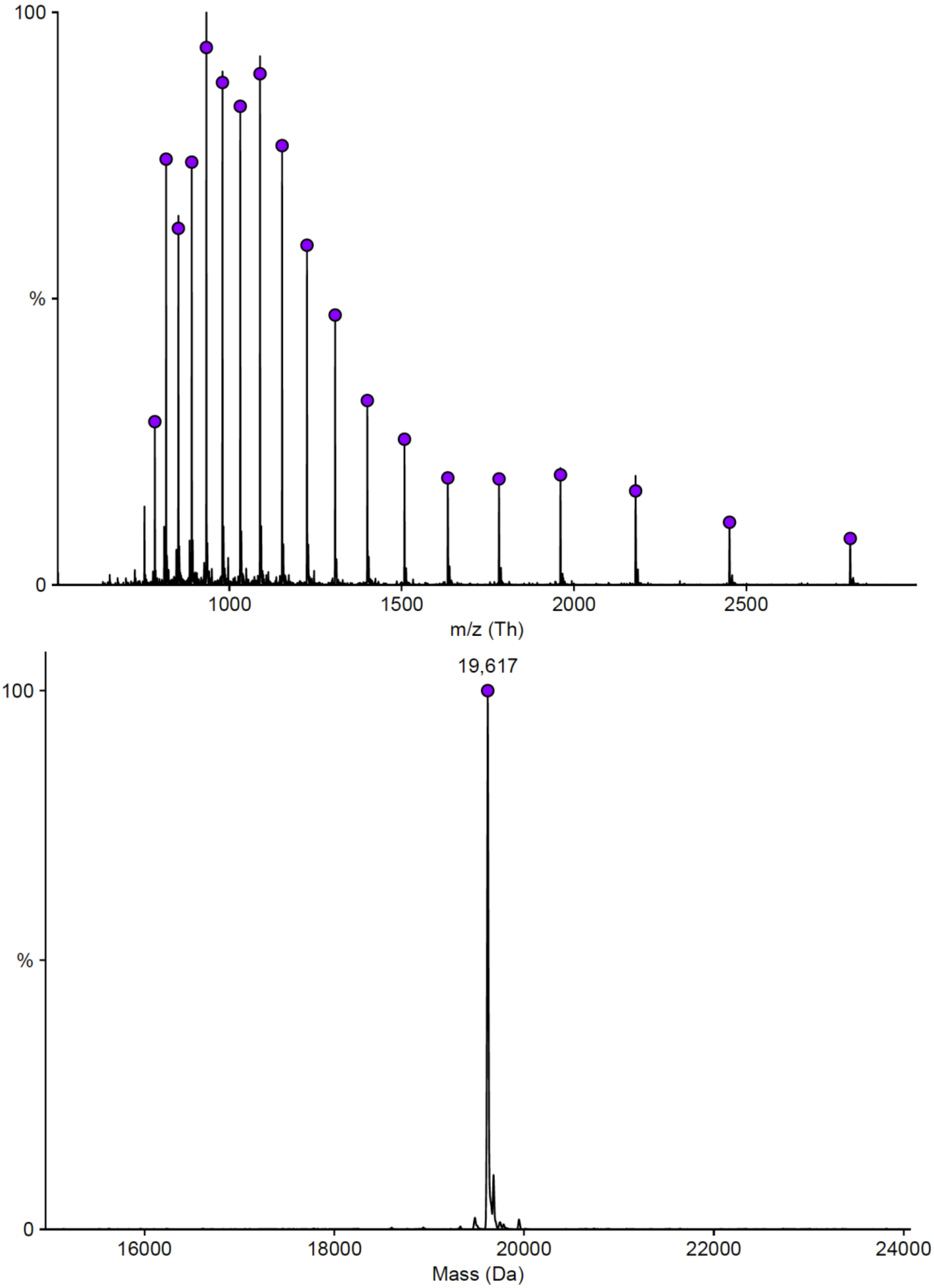

Supplementary Figure 71. Raw and deconvoluted MS spectrum of 5´-Cy5-DIR-5, calculated mass: 19618 Da, found mass: 19617 Da.

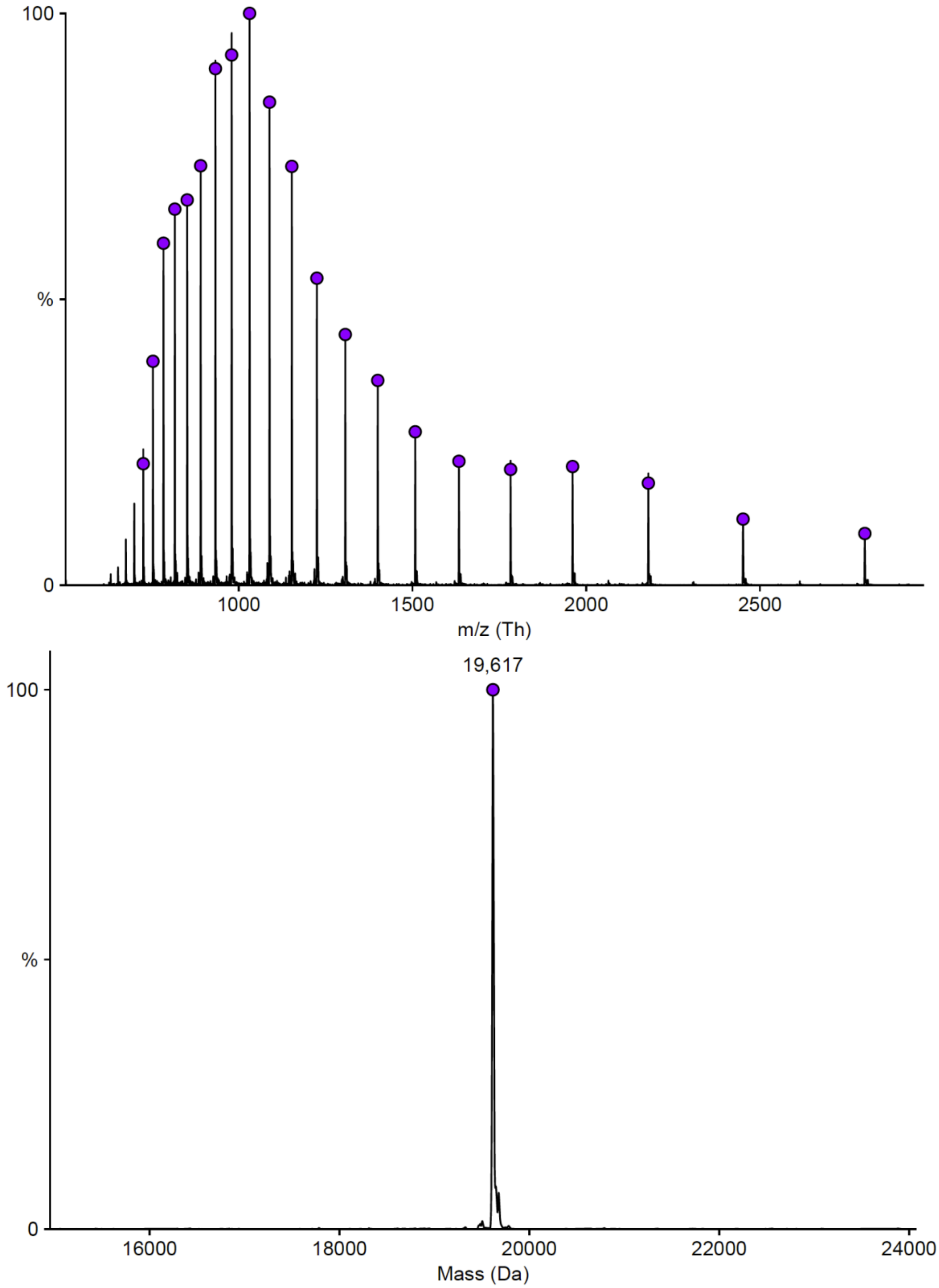

Supplementary Figure 72. Raw and deconvoluted MS spectrum of 5´-Cy5-DIR_SC, calculated mass: 19618 Da, found mass: 19617 Da.

13. Supplementary References

1. Ondruš, M., Sýkorová, V., Bednárová, L., Pohl, R., Hocek, M.: "Enzymatic Synthesis of Hypermodified DNA Polymers for Sequence-Specific Display of Four Different Hydrophobic Groups" Nucleic Acids Res. **48**, 11982-11993 (2020).

2. Altschul, S. F., Gish, W., Miller, W., Myers, E. W. & Lipman, D. J. Basic local alignment search tool. *J. Mol. Biol.* **215**, 403–410 (1990).

3. Edgar, R. C. MUSCLE: multiple sequence alignment with high accuracy and high throughput. *Nucleic Acids Res.* **32**, 1792–1797 (2004).

4. Kumar, S. et al. MEGA12: molecular evolutionary genetics analysis version 12 for adaptive and green computing. *Mol. Biol. Evol.* **41**, 1–9 (2024).

5. Waterhouse, A. M., Procter, J. B., Martin, D. M. A., Clamp, M. & Barton, G. J. Jalview Version 2—a multiple sequence alignment editor and analysis workbench. *Bioinformatics* **25**, 1189–1191 (2009).

6. Zheng, S. Q. et al. MotionCor2: anisotropic correction of beam-induced motion for improved cryo-electron microscopy. *Nat. Methods* **14**, 331–332 (2017).

7. Rosenthal, B. & Henderson, R. Optimal determination of particle orientation, absolute hand, and contrast loss in single-particle electron cryomicroscopy. *J. Mol. Biol.* **333**, 721–745 (2003).

8. Croll, T. I. et al. Higher-resolution structure of the human insulin receptor ectodomain: multi-modal inclusion of the insert domain. *Structure* **24**, 469–476 (2016).

9. Kim, J. et al. Functional selectivity of insulin receptor revealed by aptamer-trapped receptor structures. Nat. Commun. **13**, 6500 (2022).

10. Uchikawa, E., Choi, E., Shang, G., Yu, H. & Bai, X. C. Activation mechanism of the insulin receptor revealed by cryo-EM structure of the fully liganded receptor-ligand complex. Elife **8**. https://doi.org/10.7554/eLife.48630 (2019).

11. Yunn, N.-O., Kim, J., Ryu, S. H. & Cho, Y. A stepwise activation model for the insulin receptor. Exp. Mol. Med. **55**, 2147–2161 (2023).
