# Supplementary material for: Expedient single-round selection of hyper-modified aptamer targeting insulin receptor from over-represented dually nucleobase-modified DNA libraries
Source: Nat Commun. 2026 May 27;17:6895. doi: 10.1038/s41467-026-73676-y (PMC13389011; doi:10.1038/s41467-026-73676-y)
Supplement: Supplementary file 2 — Reporting Summary [file 41467_2026_73676_MOESM2_ESM.pdf]

nature portfolio

Corresponding author(s): Marek Ondruš, Michal Hocek

Last updated by author(s): May 7, 2026

Reporting Summary

Nature Portfolio wishes to improve the reproducibility of the work that we publish. This form provides structure for consistency and transparency in reporting. For further information on Nature Portfolio policies, see our Editorial Policies and the Editorial Policy Checklist.

Statistics

For all statistical analyses, confirm that the following items are present in the figure legend, table legend, main text, or Methods section.

n/a	Confirmed	
☐	☒	The exact sample size (*n*) for each experimental group/condition, given as a discrete number and unit of measurement
☐	☒	A statement on whether measurements were taken from distinct samples or whether the same sample was measured repeatedly
☒	☐	The statistical test(s) used AND whether they are one- or two-sided<br>*Only common tests should be described solely by name; describe more complex techniques in the Methods section.*
☒	☐	A description of all covariates tested
☒	☐	A description of any assumptions or corrections, such as tests of normality and adjustment for multiple comparisons
☐	☒	A full description of the statistical parameters including central tendency (e.g. means) or other basic estimates (e.g. regression coefficient) AND variation (e.g. standard deviation) or associated estimates of uncertainty (e.g. confidence intervals)
☒	☐	For null hypothesis testing, the test statistic (e.g. *F*, *t*, *r*) with confidence intervals, effect sizes, degrees of freedom and *P* value noted<br>*Give P values as exact values whenever suitable.*
☒	☐	For Bayesian analysis, information on the choice of priors and Markov chain Monte Carlo settings
☒	☐	For hierarchical and complex designs, identification of the appropriate level for tests and full reporting of outcomes
☒	☐	Estimates of effect sizes (e.g. Cohen's *d*, Pearson's *r*), indicating how they were calculated

Our web collection on statistics for biologists contains articles on many of the points above.

Software and code

Policy information about availability of computer code

Data collection	CFX96 Real-time System thermal cycler (Bio-Rad), Nanophotometer N60 (IMPLEN), Typhoon FLA 9500 (GE Healthcare Life Sciences), 1290 Infinity II Bio system (Agilent), Spark multimode microplate reader (Tecan), MonolithX MM-286 (NanoTemper Technologies), Octet RED96 (ForteBio), ChemiDoc MP Imaging System (Bio-Rad), 2470 WIZARD2 Automatic Gama Counter (PerkinElmer), Titan Krios (FEI) + Serial EM 4.1, Avance III HD (Bruker)
Data analysis	GraphPad Prism (v5, v8.01), BIAevaluation software 4.1 (Biacore), Cutadapt v4.4 , fastq-join v1.3.1, NGmerge v0.1, Seqkit v2.5.1, FASTX-Toolkit v0.0.13, FASTAptameR 2.0, MEGA12, Jalview2.11.5.0, Pymol 3.0.2, UCSF ChimeraX 1.6.1, MestReNova (14.3.3-33362), MotionCor2 v1.3.1, CTFFIND4.1, TOPAZ v0.2.5, RELION 4.0, CCP-EM 1.5, LocScale 0.1, cryoEF v1.1.06, Molprep 11.7.02, Coot 0.8.9.1, AceDRG version 277 under CCP4i2 8.0.017, CCP4 Interface 8.0.010, Refmac 5.8.0405, Phenix 1.21-5207, PDBe PISA v1.52, UniDec (8.0.1), BLAST (2.17.0), Python(3.12.4) packages: pandas(2.3.1), numpy(2.2.5), logomaker(0.8), matplotlib(3.8.4), tqdm(4.67.1), seaborn(0.13.2). R(4.5.3) packages: ggplot2(4.0.3), dplyr(1.2.1), magrittr(2.0.5), OpenLab Chemstation (2.3.54).

For manuscripts utilizing custom algorithms or software that are central to the research but not yet described in published literature, software must be made available to editors and reviewers. We strongly encourage code deposition in a community repository (e.g. GitHub). See the Nature Portfolio guidelines for submitting code & software for further information.

Data

Policy information about availability of data

All manuscripts must include a data availability statement. This statement should provide the following information, where applicable:

- Accession codes, unique identifiers, or web links for publicly available datasets
- A description of any restrictions on data availability
- For clinical datasets or third party data, please ensure that the statement adheres to our policy

The source data supporting the findings of this study are available in the Supplementary Information and Source Data files, as well as in the BioStudies public repository under accession code S-BSST2268 (https://doi.org/10.6019/S-BSST2268). Model coordinates and maps for the HIR-HIR-6 complex were deposited in the PDB and MEDB databases under accession codes 9SA8 (https://doi.org/10.2210/pdb9SA8/pdb) and EMD-54689 (https://www.ebi.ac.uk/pdbe/entry/emdb/EMD-54689), respectively.

Research involving human participants, their data, or biological material

Policy information about studies with human participants or human data. See also policy information about sex, gender (identity/presentation), and sexual orientation and race, ethnicity and racism.

Reporting on sex and gender	Not relevant, since no experiments on humans were performed in this study.
Reporting on race, ethnicity, or other socially relevant groupings	Not relevant, since no experiments on humans were performed in this study.
Population characteristics	Not relevant, since no experiments on humans were performed in this study.
Recruitment	Not relevant, since no experiments on humans were performed in this study.
Ethics oversight	Not relevant, since no experiments on humans were performed in this study.

Note that full information on the approval of the study protocol must also be provided in the manuscript.

Field-specific reporting

Please select the one below that is the best fit for your research. If you are not sure, read the appropriate sections before making your selection.

☒ Life sciences ☐ Behavioural & social sciences ☐ Ecological, evolutionary & environmental sciences

For a reference copy of the document with all sections, see nature.com/documents/nr-reporting-summary-flat.pdf

Life sciences study design

All studies must disclose on these points even when the disclosure is negative.

Sample size	No sample size calculations were performed. Since obtained data from all replicates, when applicable, were consistent, no further replications were carried out.
Data exclusions	2 experimental points from Fig. 1D (candidate 1 and candidate 5 of L2) were excluded as outliers.
Replication	The number of times each experiment was replicated is indicated in the figure captions.
Randomization	Randomization was not performed. Biochemical experiments were performed with purified and characterized (bio)molecules. At least two cell batches were tested and the results were consistent.
Blinding	Blinding was not relevant because animal or human participants were no used in this study and no allocations to experimental groups were performed because it is not typically required in aptamer selection experiments.

Reporting for specific materials, systems and methods

We require information from authors about some types of materials, experimental systems and methods used in many studies. Here, indicate whether each material, system or method listed is relevant to your study. If you are not sure if a list item applies to your research, read the appropriate section before selecting a response.

Materials & experimental systems

n/a	Involved in the study
[ ]	[x] Antibodies
[ ]	[x] Eukaryotic cell lines
[x]	[ ] Palaeontology and archaeology
[x]	[ ] Animals and other organisms
[x]	[ ] Clinical data
[x]	[ ] Dual use research of concern
[x]	[ ] Plants

Methods

n/a	Involved in the study
[x]	[ ] ChIP-seq
[x]	[ ] Flow cytometry
[x]	[ ] MRI-based neuroimaging

Antibodies

Antibodies used	Phospho-IGF-I Receptor β (Tyr1135/1136)/Insulin Receptor β (Tyr1150/1151) (19H7) Rabbit (Cell Signaling Technology, 3024)<br>Akt (pan) (C67E7) Rabbit (Cell Signaling Technology, 4691)<br>Phospho-Akt (Thr308) (C31E5E) Rabbit (Cell Signaling Technology, 2965)
Validation	Antibodies were comercially acquired and not further validated in our laboratory.

Eukaryotic cell lines

Policy information about cell lines and Sex and Gender in Research

Cell line source(s)	Human IM-9 lymphocytes (ATCC, CCL-159TM) and mouse embryonic fibroblasts derived from IGF-1R knockout mice stably transfected with human IR-A provided by A. Belfiore (Catanzaro, Italy) and R. Baserga (Philadelphia, Pennsylvania, USA).
Authentication	Cell lines were obtained from trusted source and not further authenticated in our laboratory.
Mycoplasma contamination	Cell lines are tested on a regular basis and infected cell lines are discarded.
Commonly misidentified lines (See ICLAC register)	None were used.

Plants

Seed stocks	Not relevant, as no experiments on plants were performed in this study.
Novel plant genotypes	Not relevant, as no experiments on plants were performed in this study.
Authentication	Not relevant, as no experiments on plants were performed in this study.
