# Supplementary material for: Expedient single-round selection of hyper-modified aptamer targeting insulin receptor from over-represented dually nucleobase-modified DNA libraries
Source: Nat Commun. 2026 May 27;17:6895. doi: 10.1038/s41467-026-73676-y (PMC13389011; doi:10.1038/s41467-026-73676-y)
Supplement: Supplementary file 3 — Transparent Peer Review file [file 41467_2026_73676_MOESM3_ESM.pdf]

nature portfolio

Peer Review File

Expedient Single-round Selection of Hyper-modified Aptamer Targeting Insulin Receptor from Over-represented Dually Nucleobase-modified DNA Libraries

Corresponding Author: Professor Michal Hocek

This file contains all reviewer reports in order by version, followed by all author rebuttals in order by version.

Version 0:

Reviewer comments:

Reviewer #1

(Remarks to the Author)
In this article, the authors report a one-step, modified aptamer selection method. First, modified libraries were prepared by primer extension reactions using a combination of modified dTTP and dATP which display different linker arm compositions. PEX reactions were performed on a short, randomized library (N15) to ensure a complete sequence coverage during the selection step. The resulting modified libraries were then subjected to a single-round, two partition step selection protocol with Human Insulin Receptor (HIR) as target. NGS analysis of the pooled binding libraries displayed significant enrichment only in the selection experiments carried out with modified libraries. Evaluation of binding of twenty-nine representative sequences from the main clusters resulted in the identification of two candidates which were chemically synthesized and characterized. Both sequences bound well to the target and one sequence, HIR-6, displayed a Kd value in the very low nM range. This sequence required all modified nucleotides for binding and displayed a high specificity since no binding to unrelated proteins could be detected. In addition, aptamer HIR-6 could outcompete insulin for binding to HIR. Finally, the authors resolved the cryo-EM structure of the aptamer-HIR protein target which allowed to better understand the binding mechanism. The manuscript is well-written, all experiments were carried out in a competent manner, and the supporting information is of excellent quality. This is a very interesting article that will be helpful to accelerate identification of (modified) aptamers and will thus be of interest to a broad readership. Publication in Nature Communications is therefore recommended pending some revisions:

- The main aim, i.e. isolating highly potent aptamers in a single round SELEX, was clearly achieved. Nonetheless, the authors cannot exclude that by carrying out additional rounds of SELEX (instead of just one or using a 21% mutagenized HIR-6 sequence) yet more potent sequences could have been identified. Hence a traditional SELEX with one of the modified libraries should be carried out and directly compared to the single step protocol. This is very important to further evaluate the efficiency and advantages of the one round mod-SELEX at least in this particular target case.
- The authors have selected an N15 randomized library but how would a slightly longer e.g. N22 library affect the outcome of the one-step selection? An N22 would be a good compromise since it still allows for a complete sequence coverage but allow more configurational possibilities than the smaller N15. Such an additional selection experiment will provide further proof that the modifications compensate for the smaller library size.
- Discussion: a recent hydrogel-based, one-step aptamer selection method allowed for the identification of low nM (comparable to that of HIR-6) unmodified aptamers (Nat Biotechnol 2024, 42, 1224-1231). This method used a much larger randomized domain (N40) and no chemical modifications. This should be included in the discussion (and potentially in the introduction) and both methods should be compared.

Reviewer #2

(Remarks to the Author)
This study introduces a strategy that uses modified DNA libraries to isolate high-affinity aptamers without iterative cycles. Applying this to the human insulin receptor (HIR) could yields modified aptamers, named HIR-6 and HIR-8, with high affinity and specificity for the HIR target (sub-nanomolar to picomolar binding affinity).
Cryo-EM at 2.9 Å enabled to resolve a complex in which HIR-6 is bound to FnIII-1 and L2 domains of HIR, revealing a stem-

loop structure featuring a hypermodified loop stabilized by both canonical and non-canonical interactions. Particularly noteworthy are H-bonding, T-shaped π-stacking, and CH–π contacts, that establish how the modified moieties shape aptamer geometry. The mapping of protein-aptamer contacts supports the claim that the modifications underpin both affinity and specificity. Covariation analysis and truncation results both converge on the architecture observed in cryo-EM, providing strong internal consistency to the structural model.
Overall, the structural work is well integrated with biochemical, sequencing, and mutational data, providing a coherent mechanistic explanation for high affinity, specificity, and antagonistic effect. Some modifications can improve the manuscript for publication.

Points for clarification and improvement
1. While global resolution (2.9 Å) is reported, the manuscript would benefit from explicit presentation of local resolution, map-model FSC curves, and validation parameters (Ramachandran, EMRinger, MolProbity, clashscore). These exist in Supplementary part, but a more explicit summary in the main text would strengthen structural rigor.
2. The interpretation of indole and phenyl group positioning is central to the mechanistic claims. The text refers to clear visualization of these groups, and shown in S24 f,g. However, including map snapshots with contoured densities around modified bases and the interacting residues would enhance confidence.
3. The cryo-EM model includes nucleotides C20–G38 (19 nt of the aptamer), which is appropriate given disorder of termini. Still, a brief rationale (beyond poor density) on whether the unmodeled regions adopt transient interactions or remain flexible would be helpful for readers interpreting the functional contributions of truncated regions.
4. The discussion briefly mentions the A62 and A43 aptamers, but a more broad and integrative comparison of binding footprints, conformational effects on HIR, and differences in receptor activation states would enrich the structural interpretation.
5. The low-resolution overview (Fig. 5A) indicates two aptamers bound per receptor dimer. It would be useful to comment on whether both sites exhibit equivalent binding geometry or whether heterogeneity was observed during classification.

Reviewer #3

(Remarks to the Author)
The authors utilized base-modified monomers to construct chemically expanded random-sequence libraries, performed multiple parallel selections, and, through a single round of selection (SRS) supplemented with NGS sequencing, identified several chemically modified aptamers binding to the human insulin receptor (HIR). The best-performing aptamer exhibited binding affinity in the low nanomolar to hundred-picomolar range. The aptamer demonstrated binding specificity. Specific sequences and chemical modifications were also shown to be essential for the aptamer's binding ability. The aptamer was able to inhibit insulin-induced activation of HIR with an IC50 around 100 nM. The authors predicted the aptamer's secondary structure via covariation analysis. Finally, the authors determined the structure of the aptamer-HIR complex using cryo-EM, highlighting the importance of specific chemical modifications (phenyl and indole rings) in the interaction between the aptamer and HIR.

The manuscript presents a comprehensive and well-executed study. The authors clearly demonstrate the application of a novel SRS strategy using chemically expanded libraries to successfully identify high-affinity aptamers targeting HIR. This work is particularly impressive given the inherent challenges in selecting modified aptamers. The lead aptamer exhibits outstanding properties, including picomolar affinity, exquisite specificity, and potent functional antagonism, that rival or surpass those obtained through conventional multi-round SELEX. Furthermore, the study extends beyond typical aptamer characterization by providing deep mechanistic insights through covariation analysis and, most notably, a high-resolution cryo-EM structure of the aptamer-receptor complex, which elegantly elucidates the critical roles of the dual hydrophobic modifications.

I am supportive of publication in Nature Communications, pending satisfactory revision to address the specific points outlined below.

1. Potential aptamers "were screened for affinity to HIR using a fluorescent Ni-plate binding (FNBA) assay". It is recommended that the authors briefly explain the principle of this method in the main text to help readers understand the relationship between fluorescence values and aptamer affinity in figures like Fig. 1D.
2. In Fig. 1H, why aren't the data grouped by cluster? Presenting them by cluster would better illustrate the statement that "Sequences from cluster 1 … exhibited the highest binding affinity compared to other clusters".
3. Are the 25 candidates in Fig. 1H all from the L2' library selection? The previous selections using L2 and L3 also yielded aptamers. Have the affinities of aptamers from these different libraries been compared under identical conditions? Are HIR-6 and HIR-8, chosen for follow-up studies, among the 25 candidates from the L2' selection?
4. In Fig. 2B, the authors tested unmodified and scrambled versions of HIR-6 and HIR-8. Does "unmodified" refer to molecules with the same sequence but with the modification groups on A and U removed? Similarly, does "scrambled" refer to molecules with the chemical modification groups and proportions kept but the sequence order randomized? Fig. 2B presents the unmodified and scrambled versions for HIR-6, but only the unmodified version for HIR-8, not the scrambled version. Why is that?
5. In Fig. 3B, for the red curve, the x-axis concentration represents insulin concentration. However, for the blue and black curves, the insulin concentration is fixed, and the x-axis concentration represents aptamer concentration. This presentation could easily cause confusion or misunderstanding for readers.

Reviewer #4

(Remarks to the Author)
The authors present a single-round selection strategy for discovering high-affinity DNA aptamers. Unlike conventional SELEX, which requires multiple rounds of enrichment, this method employs a short, chemically diverse library to isolate potent binders in a single round. Using this approach, the authors successfully identify HIR-6, a modified DNA aptamer that binds the human insulin receptor with picomolar affinity and high specificity. Cryo-EM analysis reveals that HIR-6 competes with insulin at binding site 2, stabilizes the receptor in its autoinhibited conformation, and functions as an insulin antagonist by blocking insulin-stimulated autophosphorylation. Overall, this is an interesting study, and the approach has potential to streamline therapeutic aptamer discovery. The cryo-EM work appears to be of high quality. However, several concerns should be addressed before the manuscript is suitable for publication in Nature Communications:

1. The authors report that HIR-6 is highly specific for the human insulin receptor and does not bind dog or cat insulin receptors or the human IGF1 receptor, despite their high sequence similarity. To demonstrate that this newly developed platform is broadly applicable for aptamer discovery, the authors should apply the same pipeline to isolate aptamers against one of these related receptors (e.g., dog IR, cat IR, or human IGF1R). Although solving an additional cryo-EM structure is unnecessary, the authors should provide binding and functional assays showing that the method can generate specific aptamers for targets beyond the human insulin receptor.

2. It is somewhat disappointing that HIR-6 acts as an antagonist, as insulin receptor inhibitors have limited therapeutic value. The authors should explore whether HIR-6 can be converted into an agonist, for example by linking two HIR-6 molecules to generate a dimeric form, similar to the strategy described in PMID: 40603733. An insulin receptor agonist could potentially serve as an insulin alternative. Demonstrating agonist activity would substantially enhance the impact of the study.

3. The methods section lacks essential information on the expression and purification of the proteins used in this study (human, dog, and cat insulin receptors; human IGF1 receptor; etc.). In addition, the manuscript provides no biochemical characterization of these proteins (e.g., size-exclusion chromatography profiles and SDS-PAGE analyses). This is concerning, as accurate binding affinity measurements depend strongly on protein quality and behavior. The authors should include complete protein production details in the method section and provide supporting protein biochemical data.

Version 1:

Reviewer comments:

Reviewer #1

(Remarks to the Author)
This manuscript was evaluated by four reviewers (I was reviewer#1) who were rather favorable for publication but still raised numerous issues. The authors have now addressed all the concerns and comments raised by the reviewers and following these comments have included additional experiments. Notably, the authors have carried out an additional selection experiment against DIR as target (as recommended by reviewer#4) and evaluated other short libraries (as recommended by reviewer#1). In addition, the authors addressed the concerns raised by reviewer#2 on the structural analysis of the aptamer-protein complex and the response to reviewer#3 was also satisfactory. Overall, the authors have addressed all concerns and significantly improved the quality of manuscript which can be considered for publication in Nature Communications without additional revisions.

Reviewer #2

(Remarks to the Author)
The authors have addressed all my previous concerns very effectively. The inclusion of additional validation metrics, improved visualization of the density, clearer justification of the modeled regions, and expanded structural comparisons significantly strengthen the manuscript. I have no further concerns, and I consider the manuscript suitable for publication in Nature Communications.

Reviewer #3

(Remarks to the Author)
The authors have satisfactorily addressed my concerns. I support publication in Nature Communications.

Reviewer #4

(Remarks to the Author)
I am satisfied with the revision.

Open Access This Peer Review File is licensed under a Creative Commons Attribution 4.0 International License, which permits use, sharing, adaptation, distribution and reproduction in any medium or format, as long as you give appropriate credit to the original author(s) and the source, provide a link to the Creative Commons license, and indicate if changes were made.
In cases where reviewers are anonymous, credit should be given to 'Anonymous Referee' and the source.
The images or other third party material in this Peer Review File are included in the article’s Creative Commons license, unless indicated otherwise in a credit line to the material. If material is not included in the article’s Creative Commons license and your intended use is not permitted by statutory regulation or exceeds the permitted use, you will need to obtain permission directly from the copyright holder.
To view a copy of this license, visit https://creativecommons.org/licenses/by/4.0/

Response to reviewers and list of changes

Nature Communications manuscript NCOMMS-25-85604

Over-Represented DNA Libraries Containing Two Hydrophobic (Het)aryl-Linked Nucleotides for Expedient Single-Round Selection of a Specific Aptamer Targeting Human Insulin Receptor

REVIEWER COMMENTS

Reviewer #1 (Remarks to the Author):

In this article, the authors report a one-step, modified aptamer selection method. First, modified libraries were prepared by primer extension reactions using a combination of modified dTTP and dATP which display different linker arm compositions. PEX reactions were performed on a short, randomized library (N15) to ensure a complete sequence coverage during the selection step. The resulting modified libraries were then subjected to a single-round, two partition step selection protocol with Human Insulin Receptor (HIR) as target. NGS analysis of the pooled binding libraries displayed significant enrichment only in the selection experiments carried out with modified libraries. Evaluation of binding of twenty-nine representative sequences from the main clusters resulted in the identification of two candidates which were chemically synthesized and characterized. Both sequences bound well to the target and one sequence, HIR-6, displayed a Kd value in the very low nM range. This sequence required all modified nucleotides for binding and displayed a high specificity since no binding to unrelated proteins could be detected. In addition, aptamer HIR-6 could outcompete insulin for binding to HIR. Finally, the authors resolved the cryo-EM structure of the aptamer-HIR protein target which allowed to better understand the binding mechanism. The manuscript is well-written, all experiments were carried out in a competent manner, and the supporting information is of excellent quality. This is a very interesting article that will be helpful to accelerate identification of (modified) aptamers and will thus be of interest to a broad readership. Publication in Nature Communications is therefore recommended pending some revisions:
We thank the reviewer for the very positive assessment of our paper.

- The main aim, i.e. isolating highly potent aptamers in a single round SELEX, was clearly achieved. Nonetheless, the authors cannot exclude that by carrying out additional rounds of SELEX (instead of just one or using a 21% mutagenized HIR-6 sequence) yet more potent

sequences could have been identified. Hence a traditional SELEX with one of the modified libraries should be carried out and directly compared to the single step protocol. This is very important to further evaluate the efficiency and advantages of the one round mod-SELEX at least in this particular target case.

Response: Direct comparison of SRS and multiple-round SELEX is not feasible because of the differences in the size and diversity of the library. In our SRS we use rather small over-represented libraries with short randomized region (N13-17), whereas the classical SELEX uses much larger libraries (~N40). Performing multiple-round SELEX with the very short library would not bring any advantage – it would rather combine disadvantages of both approaches (laborious procedure and less-diverse library), while multiple-round SELEX from large library would probably lead to completely different aptamers. However, there are several literature examples of multiple-round SELEX for aptamers against HIR even with modified nucleotides and our **HIR-6** aptamer has the same or better affinity and higher specificity.

Revision: we have modified the last paragraph of the Discussion to highlight the differences and advantages of the SRS approach over standard SELEX.

- The authors have selected an N15 randomized library but how would a slightly longer e.g. N22 library affect the outcome of the one-step selection? An N22 would be a good compromise since it still allows for a complete sequence coverage but allow more configurational possibilities than the smaller N15. Such an additional selection experiment will provide further proof that the modifications compensate for the smaller library size.

Response: We thank for this important suggestion. The N22 library is too large for SRS as it contains only ~10 copies of each sequence, but we agree that we should have studied the scope of the randomized region size and its feasibility for SRS. Therefore, we have synthesized a set of N13, N17, N19 and N22 libraries and used them for SRS. The results obtained show that N13 and N17 contained enough sequence over-representation for a rapid aptamer identification, while N19 and longer were too large and insufficiently represented and did not show any enrichment in the SRS approach (Supplementary Figs. S12-S16).

Revision: We added a paragraph titled "Number of randomized positions matters – higher sequence over-representation determines single-round performance of modified libraries" and experiment-related figures (Supplementary Figs. S12-16, S26-27, S33 and Tables S9-10) to SI.

- Discussion: a recent hydrogel-based, one-step aptamer selection method allowed for the identification of low nM (comparable to that of HIR-6) unmodified aptamers (Nat Biotechnol 2024, 42, 1224-1231). This method used a much larger randomized domain (N40) and no chemical modifications. This should be included in the discussion (and potentially in the introduction) and both methods should be compared.

Response: We thank the reviewer for bringing this important work to our attention. Indeed, the PEG hydrogel-based method can achieve a high partitioning efficiency of binding sequences from the non-binding sequences. It is difficult to compare the method to our approach but certainly it deserves to be mentioned and cited.

Revision: The following sentences were added to the main manuscript:

"More recently, high partition efficiency was alternatively achieved using a non-fouling PEG hydrogel that minimizes nonspecific adsorption."

"However, non-exponential enrichment may be increased by enhancing partition efficiency with the PEG-based hydrogel matrix, as shown for conventional unmodified libraries. Therefore, a combination of these two technological advances can be further beneficial for SRSs."

Reviewer #2 (Remarks to the Author):

This study introduces a strategy that uses modified DNA libraries to isolate high-affinity aptamers without iterative cycles. Applying this to the human insulin receptor (HIR) could yields modified aptamers, named HIR-6 and HIR-8, with high affinity and specificity for the HIR target (sub-nanomolar to picomolar binding affinity).
Cryo-EM at 2.9 Å enabled to resolve a complex in which HIR-6 is bound to FnIII-1 and L2 domains of HIR, revealing a stem-loop structure featuring a hypermodified loop stabilized by both canonical and non-canonical interactions. Particularly noteworthy are H-bonding, T-shaped π-stacking, and CH–π contacts, that establish how the modified moieties shape aptamer geometry. The mapping of protein-aptamer contacts supports the claim that the modifications underpin both affinity and specificity. Covariation analysis and truncation results both converge on the architecture observed in cryo-EM, providing strong internal consistency to the structural model.
Overall, the structural work is well integrated with biochemical, sequencing, and mutational data, providing a coherent mechanistic explanation for high affinity, specificity, and antagonistic effect. Some modifications can improve the manuscript for publication.
We thank the reviewer for the positive comments and constructive feedback.

Points for clarification and improvement

1. While global resolution (2.9 Å) is reported, the manuscript would benefit from explicit presentation of local resolution, map-model FSC curves, and validation parameters (Ramachandran, EMRinger, MolProbity, clashscore). These exist in Supplementary part, but a more explicit summary in the main text would strengthen structural rigor.

Response: We agree with the reviewer that this will strengthen the structural rigor for the readers.

Revision: We added the EMRinger statistics (EMRigner score 4.46) into Table S13 and into the methods section (SI lines 904-906). We added the map to model FSC curves to Figure S41 (unmasked map to model FSC is 2.86 Å at the 0.5 threshold). We also briefly stated the most important model validation statistics in the main text (lines 429-430).

2. The interpretation of indole and phenyl group positioning is central to the mechanistic claims. The text refers to clear visualization of these groups, and shown in S24 f,g. However, including map snapshots with contoured densities around modified bases and the interacting residues would enhance confidence.

Response: We thank the reviewer for this helpful comment.

Revision: We replaced panels f and g in Figure S41 (previously S24), which showed contoured densities around two individual modified bases, with new panels g, h, and i, which show densities around sections of the views shown in Fig. 6a,b,c. Multiple modified bases are now depicted together with interacting residues. We believe that the high atom inclusion (0.8290, PDB validation report), relatively high overall Q-score (0.578, PDB validation report), and the EMRigner score (4.46, Table S13) together with other reported metrics indicate high confidence of the proposed atomic model.

3. The cryo-EM model includes nucleotides C20–G38 (19 nt of the aptamer), which is appropriate given disorder of termini. Still, a brief rationale (beyond poor density) on whether the unmodeled regions adopt transient interactions or remain flexible would be helpful for readers interpreting the functional contributions of truncated regions.

Response: In order to rigorously interpret the obtained cryo-EM densities we only modeled the well-defined parts of the **HIR-6** aptamer. Beyond the C20–G38 base pair the atomic details are not well resolved. There is a blurred density which supports the base pairing of T19-A39 but is not suitable for model building. The base pairing of T19-A39 observed in the density corelates with the mutual proximity of these two residues identified in the

covariation analysis (Figure 4D). Beyond T19-A39, the cryo-EM density is not interpretable. Using the cryo-EM data, we are not able to determine whether there are or are not other additional interactions in the unmodeled regions of the **HIR-6** aptamer. In reflection to this comment, we also improved the diagram in Figure 4D to depict more clearly the proximity of aptamer bases based on the covariation analysis.

Revision: We altered the text accordingly in the results and methods section (lines 433-435, SI line 886).

4. The discussion briefly mentions the A62 and A43 aptamers, but a more broad and integrative comparison of binding footprints, conformational effects on HIR, and differences in receptor activation states would enrich the structural interpretation.

Response: We thank the referee for this comment.

Revision: As suggested, we added Supplementary Figure S43 and a new discussion paragraph (lines 637-647), where we depict and compare the binding footprints of **HIR-6** and aptamers A62 and A43, together with the conformational effects on HIR and the related receptor activation states. We also added to the comparison antibody Fab 83-14 that stabilizes the inactive HIR conformation. We agree with the reviewer that such an integrative comparison aids the discussion and puts our structural interpretation into the perspective of other structurally characterized HIR aptamers.

5. The low-resolution overview (Fig. 5A) indicates two aptamers bound per receptor dimer. It would be useful to comment on whether both sites exhibit equivalent binding geometry or whether heterogeneity was observed during classification.

Response: During the 3D classification, the defined 3D classes of HIR-**HIR-6** complex have always two **HIR-6** aptamers bound per receptor dimer in a symmetrical fashion. However, the final HIR-**HIR-6** complex is not strictly C2 symmetric, and this is pronounced in the FnIII-2 and FnIII-3 regions of the complex, owing to the inherent flexibility of the receptor. Because of that we 3D classified and 3D refined the HIR-**HIR-6** in C1 symmetry with C2 relaxation (Figure S42, bottom). Nevertheless, in the final high-resolution HIR-**HIR-6** complex the aptamer interface and binding geometry in respect to the FnIII-1 and L2 domains are identical.

Revision: we clarified the sentence: conformation of the receptor with two equivalent molecules of **HIR-6** aptamers bound to the FnIII-1 and L2 domains

Reviewer #3 (Remarks to the Author):

The authors utilized base-modified monomers to construct chemically expanded random-sequence libraries, performed multiple parallel selections, and, through a single round of selection (SRS) supplemented with NGS sequencing, identified several chemically modified aptamers binding to the human insulin receptor (HIR). The best-performing aptamer exhibited binding affinity in the low nanomolar to hundred-picomolar range. The aptamer demonstrated binding specificity. Specific sequences and chemical modifications were also shown to be essential for the aptamer's binding ability. The aptamer was able to inhibit insulin-induced activation of HIR with an IC50 around 100 nM. The authors predicted the aptamer's secondary structure via covariation analysis. Finally, the authors determined the structure of the aptamer-HIR complex using cryo-EM, highlighting the importance of specific chemical modifications (phenyl and indole rings) in the interaction between the aptamer and HIR.

The manuscript presents a comprehensive and well-executed study. The authors clearly demonstrate the application of a novel SRS strategy using chemically expanded libraries to successfully identify high-affinity aptamers targeting HIR. This work is particularly impressive given the inherent challenges in selecting modified aptamers. The lead aptamer exhibits outstanding properties, including picomolar affinity, exquisite specificity, and potent functional antagonism, that rival or surpass those obtained through conventional multi-round SELEX. Furthermore, the study extends beyond typical aptamer characterization by providing deep mechanistic insights through covariation analysis and, most notably, a high-resolution cryo-EM structure of the aptamer-receptor complex, which elegantly elucidates the critical roles of the dual hydrophobic modifications.

I am supportive of publication in Nature Communications, pending satisfactory revision to address the specific points outlined below.
We thank the reviewer for the positive assessment of our work.

1. Potential aptamers "were screened for affinity to HIR using a fluorescent Ni-plate binding (FNBA) assay". It is recommended that the authors briefly explain the principle of this method in the main text to help readers understand the relationship between fluorescence values and aptamer affinity in figures like Fig. 1D.

Revision: we added the explanation:

fluorescent Ni-plate binding assay (FNBA) - in which the HIR was immobilized onto the plate surface and where the capability of the Cy5-labeled aptamers to remain bound to HIR after several washing steps is indicated by their fluorescence intensities (Fig. 1D).

2. In Fig. 1H, why aren't the data grouped by cluster? Presenting them by cluster would better illustrate the statement that "Sequences from cluster 1 ... exhibited the highest binding affinity compared to other clusters".

Revision: Aptamer sequences were grouped by clusters as suggested.

3. Are the 25 candidates in Fig. 1H all from the L2' library selection? The previous selections using L2 and L3 also yielded aptamers. Have the affinities of aptamers from these different libraries been compared under identical conditions? Are HIR-6 and HIR-8, chosen for follow-up studies, among the 25 candidates from the L2' selection?

Response: Yes, candidates from Fig. 1H were obtained from the L2' library selection. We added "from L2' selection" and "L2'" to the Fig. 1H and 1I. Affinities of all aptamer candidates within FNBA were compared under identical conditions. L1, L2 and L3 aptamer candidates were even measured within the same plate for proper fluorescence comparison. Yes, **HIR-6** and **HIR-8** were selected from the L2' selection.

Revision: we added "of L2' selection" to the main text.

4. In Fig. 2B, the authors tested unmodified and scrambled versions of HIR-6 and HIR-8. Does "unmodified" refer to molecules with the same sequence but with the modification groups on A and U removed? Similarly, does "scrambled" refer to molecules with the chemical modification groups and proportions kept but the sequence order randomized? Fig. 2B presents the unmodified and scrambled versions for HIR-6, but only the unmodified version for HIR-8, not the scrambled version. Why is that?

Response: Yes, unmodified **HIR-6_N** and **HIR-8_N** refer to the same aptamer sequences with canonical nucleobases instead of the modified ones. Yes, scrambled sequence (**HIR_SC)** contains the same proportion of modified nucleotides that **HIR_6** and **HIR_8** with a randomized order. Due to **HIR-6** and **HIR-8** contain the same proportion of modified and unmodified nucleotides, **HIR_SC** was suitable as a scrambled control for both sequences**.** Detailed information of all sequences can be found in supplementary information document.

Revision: We added "(**HIR_SC**, shared for both aptamers)". Additionally, one missing datapoint in Fig.2A for **HIR-8** and unrelated protein HIV-CP24 specificity signal was added.

5. In Fig. 3B, for the red curve, the x-axis concentration represents insulin concentration. However, for the blue and black curves, the insulin concentration is fixed, and the x-axis concentration represents aptamer concentration. This presentation could easily cause confusion or misunderstanding for readers.

Response: We fully agree with the reviewer.

Revision: Fig. 3B was modified and insulin curve was separated and transferred to supplementary information (Supplementary Fig. S40) document to avoid misunderstanding. Additionally, one missing datapoint in the insulin curve was added.

Reviewer #4 (Remarks to the Author):

The authors present a single-round selection strategy for discovering high-affinity DNA aptamers. Unlike conventional SELEX, which requires multiple rounds of enrichment, this method employs a short, chemically diverse library to isolate potent binders in a single round. Using this approach, the authors successfully identify HIR-6, a modified DNA aptamer that binds the human insulin receptor with picomolar affinity and high specificity. Cryo-EM analysis reveals that HIR-6 competes with insulin at binding site 2, stabilizes the receptor in its autoinhibited conformation, and functions as an insulin antagonist by blocking insulin-stimulated autophosphorylation. Overall, this is an interesting study, and the approach has potential to streamline therapeutic aptamer discovery. The cryo-EM work appears to be of high quality. However, several concerns should be addressed before the manuscript is suitable for publication in Nature Communications:

1. The authors report that HIR-6 is highly specific for the human insulin receptor and does not bind dog or cat insulin receptors or the human IGF-1 receptor, despite their high sequence similarity. To demonstrate that this newly developed platform is broadly applicable for aptamer discovery, the authors should apply the same pipeline to isolate aptamers against one of these related receptors (e.g., dog IR, cat IR, or human IGF-1 R). Although solving an additional cryo-EM structure is unnecessary, the authors should provide binding and functional assays showing that the method can generate specific aptamers for targets beyond the human insulin receptor.

Response: We have successfully used the SRS methodology for other aptamers targeting important protein biomarkers (including IGF-1 receptor) – however, these results cannot be

disclosed now because the aptamers will be further optimized and patented. This paper is about the SRS methodology and we have picked the HIR as target because there is little commercial interest in it and there are already other aptamers to be compared to.

However, to demonstrate the generality of the SRS approach and following the suggestion of this Reviewer, we have now performed selection against DIR and identified an aptamer with 19 nM affinity and 4-fold specificity over HIR. It should be noted that if we seriously wanted to develop a more specific aptamer against DIR, we could screen different libraries and use HIR as off-target component in the SRS – but that would be far beyond the scope of this paper.

Revision: We added the new experiments with the selection of DIR aptamer and paragraph titled "Scope of the SRS – selection of an aptamer for the related DIR target" to the main text and experiment-related figures (Supplementary Figs. S17, S28, S29, S34 and S36) to SI.

Also, we have rewritten the conclusion paragraph of the Discussion to further highlight the methodological aspects of the work and the potential to quickly screen different libraries containing different modifications.

2. It is somewhat disappointing that HIR-6 acts as an antagonist, as insulin receptor inhibitors have limited therapeutic value. The authors should explore whether HIR-6 can be converted into an agonist, for example by linking two HIR-6 molecules to generate a dimeric form, similar to the strategy described in PMID: 40603733. An insulin receptor agonist could potentially serve as an insulin alternative. Demonstrating agonist activity would substantially enhance the impact of the study.

Response: As we discuss above, the aim of this work was not to develop a therapeutic aptamer. The goal and selling point is the fast and efficient SRS methodology using overrepresented libraries and combinations of two modified nucleotides and we demonstrated it on the case study with development of HIR aptamer. The aptamers are selected for binding – not for agonist or antagonist activity so it would be difficult to aim for agonist activity. We assume that maybe if we tested other aptamer candidates (also from other libraries), some of them might be agonists – but such study would be out of the scope of this work. Also there are plenty of insulin derivatives and analogues that work very well and are used in the clinic – we do not see a potential for a therapeutic aptamer here.

Moreover, the cryoEM structure shows that binding of two molecules of **HIR-6** aptamers stabilizes the autoinhibited conformation (see Supplementary Figure S43), so it is very unlikely that the dimeric form would switch the activity.

3. The methods section lacks essential information on the expression and purification of the proteins used in this study (human, dog, and cat insulin receptors; human IGF-1 receptor; etc.). In addition, the manuscript provides no biochemical characterization of these proteins (e.g., size-exclusion chromatography profiles and SDS-PAGE analyses). This is concerning, as accurate binding affinity measurements depend strongly on protein quality and behavior. The authors should include complete protein production details in the method section and provide supporting protein biochemical data.

Response: Catalog numbers can be found in supplementary information and biochemical protein characterization can be found on the provider website. Additionally, **HIR-6** binding to HIR while **DIR-5** binding to DIR and HIR provides additional evidence that both proteins were of good condition. We have also included Capsid protein p24 (HIV) and CPA protein into SI.

Revision: we have added more details about the proteins:

His-Tagged Human Insulin Receptor (Cat. No. INR-H52Ha), His-Tagged Human IGF-1 R (Cat. No. IGR-H5229), His-Tagged Cat Insulin Receptor (Cat. No. INR-C52H3), His-Tagged Dog Insulin Receptor (Cat. No. INR-D52H3) and Capsid protein p24 (HIV) (Cat. No. CP4-H52H3) were commercially acquired from ACROBiosystems. CPA was prepared inhouse by Milan Kožíšek (Jan Konvalinka research group, IOCB) according to published protocol.
